# Sorafenib inhibits ossification of the posterior longitudinal ligament by blocking LOXL2-mediated vascularization

**DOI:** 10.1038/s41413-024-00327-7

**Published:** 2024-04-10

**Authors:** Longqing Wang, Wenhao Jiang, Siyuan Zhao, Dong Xie, Qing Chen, Qi Zhao, Hao Wu, Jian Luo, Lili Yang

**Affiliations:** 1 Spine Center, Department of Orthopaedics, Shanghai Changzheng Hospital, Second Affiliated Hospital of Naval Medical University, Shanghai, 200003 PR China; 2 Yangzhi Rehabilitation Hospital (Shanghai Sunshine Rehabilitation Center), Tongji University School of Medicine, Shanghai, PR China; 3 Shanghai Key Laboratory of Regulatory Biology, Institute of Biomedical Sciences and School of Life Sciences, East China Normal University, Shanghai, PR China; 4 Department of Orthopaedics, No. 905 Hospital of PLA Navy, Shanghai, PR China

**Keywords:** Pathogenesis, Bone

## Abstract

Ossification of the Posterior Longitudinal Ligament (OPLL) is a degenerative hyperostosis disease characterized by the transformation of the soft and elastic vertebral ligament into bone, resulting in limited spinal mobility and nerve compression. Employing both bulk and single-cell RNA sequencing, we elucidate the molecular characteristics, cellular components, and their evolution during the OPLL process at a single-cell resolution, and validate these findings in clinical samples. This study also uncovers the capability of ligament stem cells to exhibit endothelial cell-like phenotypes in vitro and in vivo. Notably, our study identifies LOXL2 as a key regulator in this process. Through gain-and loss-of-function studies, we elucidate the role of LOXL2 in the endothelial-like differentiation of ligament cells. It acts via the HIF1A pathway, promoting the secretion of downstream VEGFA and PDGF-BB. This function is not related to the enzymatic activity of LOXL2. Furthermore, we identify sorafenib, a broad-spectrum tyrosine kinase inhibitor, as an effective suppressor of LOXL2-mediated vascular morphogenesis. By disrupting the coupling between vascularization and osteogenesis, sorafenib demonstrates significant inhibition of OPLL progression in both BMP-induced and *enpp1* deficiency-induced animal models while having no discernible effect on normal bone mass. These findings underscore the potential of sorafenib as a therapeutic intervention for OPLL.

## Introduction

Ossification of the posterior longitudinal ligament (OPLL) can be defined as a degenerative hyperostosis disease of the spine. The posterior longitudinal ligament (PLL) starts from the occiput and terminates at the sacrum. The PLL runs along the posterior side of vertebral bodies and intervertebral disks and is located in the anterior of the dural sac and spinal cord.^1,2^ If the PLL is ossified, the ossification can protrude into the spinal canal, causing severe neurological symptoms similar to myelopathy and radiculopathies like paresthesia, disturbances of motility and autonomic disorders lowering the quality of life.^3,4^

OPLL is a unique form of heterotopic ossification that typically occurs in the cervical spine and predominantly affects males.^5^ Unlike other heterotopic ossification diseases, OPLL is not associated with trauma, systemic inflammatory diseases, burns, or long-term immobilization. Our previous study found that 18% of patients suffering from degenerative cervical myelopathy (DCM) had OPLL based on 3D-CT findings.^5^ Epidemiological studies have shown that OPLL is more prevalent in East Asia, with an incidence rate ranging from 1.9% to 4.3% in Japan. In contrast, the incidence of OPLL is lower in North America and continental Europe, ranging from 0.1% to 1.7%.^6^ During the initiation and progression of OPLL, the disorder is thought to develop through endochondral ossification at the histological level.^7,8^ The avascular ligamentous tissue is progressively transformed and replaced by highly vascularised bone tissue. Numerous genes^9^ and non-coding RNAs (ncRNAs)^10^ that regulate the osteogenic differentiation of ligament cells have been identified; however, the formation of ossification encompasses various other biological processes beyond osteogenic differentiation. The infiltration of blood vessels plays a crucial role in the disease process.

Blood vessel formation involves two primary processes: vasculogenesis and angiogenesis. Vasculogenesis refers to the de novo creation of blood vessels, during which progenitor or stem cells differentiate, migrate, and assemble into a rudimentary vascular network. On the other hand, angiogenesis involves the development of new blood vessels branching out from pre-existing ones.^11,12^ Mesenchymal stromal cells (MSCs) play a critical role in angiogenesis through paracrine signaling. They secrete pro-angiogenic factors like vascular endothelial growth factor (VEGF), platelet-derived growth factor (PDGF), and fibroblast growth factor (FGF). These factors encourage endothelial cell proliferation, migration, and tube formation, facilitating the generation of new blood vessels.^13^ Additionally, under certain stimulations, MSCs can differentiate into endothelial cells (VEGFA or TNF-α^14,15^) and myocytes (5-azacytidine^16^), participating in blood vessel formation. Despite these findings, the role of resident stem cells in blood vessel formation and the prospects for therapeutic intervention, particularly in bone formation, remain incompletely understood.

The Lysyl oxidase family consists of LOX, LOXL1-4, including LOXL2, which has a highly conserved catalytic domain in the C-terminal and scavenger receptor cysteine-rich (SRCR) domains in the N-terminal.^17,18^ LOXL2 regulates blood vessel formation through collagen scaffolding.^19,20^ It is also involved in hepatocellular carcinoma vasculogenic mimicry formation, tumor cell invasion, and metastasis.^21,22^ Moreover, LOXL2 significantly affects numerous fibrotic diseases and tumors.^23–25^ Recent studies have indicated that LOXL2 can epigenetically regulate angiogenesis by deaminating trimethylated lysine 4 of histone H3 (H3K4me3) and subsequently activating transcription.^26,27^

Our analysis of sequencing data obtained from ossified (OPLL) and para-ossified (non-OPLL) ligament samples revealed an upregulation of genes and signaling pathways associated with vasculogenesis and angiogenesis in ossified tissues. Furthermore, our study demonstrated that ligament cells derived from the posterior longitudinal ligament can acquire endothelial-like phenotype. To elucidate the dynamics of this differentiation process, we conducted single-cell sequencing on human posterior longitudinal ligament tissue and constructed a pseudotime trajectory illustrating the potential differentiation of ligament cells into blood vessels. Subsequent in vitro and in vivo experiments validated these findings, confirming that ligament cells can differentiate to form functional blood vessels in the OPLL scenario. Through our loss- and gain-of-function experiments, we provided evidence that LOXL2 regulates the differentiation of ligament cells into endothelial-like cells through the HIF1A signaling pathway. Additionally, we discovered that sorafenib, a small molecule inhibitor, can counteract the enhanced endothelial-like differentiation and osteogenesis resulting from LOXL2 upregulation. Importantly, our findings were further corroborated by demonstrating the inhibitory effect of sorafenib on ossification progression in various rodent models.

## Results

### OPLL is characterized by endochondral ossification

We examined the samples from OPLL patients. The lateral X-ray images show a high-density mass similar to bone attached to the posterior edge of the vertebral body (Fig. S1a). The sagittal and transverse view of Three-dimensional reconstruction CT (3D CT) and MRI from OPLL patients shows that the ossification protrudes into the spinal canal and causes local compression, resulting the neurological symptoms (Fig. S1b). The *en bloc* specimen resected from OPLL patients showed that ossified tissue causing spinal cord compression had an abundant blood supply. To better understand the internal microstructure of the OPLL, we scanned the ossified ligaments, and lamellar trabecular structures were seen in the ossification samples (Fig. S1c). These features were not seen in control patients without ossification (Fig. S1d–f). Two scales (the modified JOA and NDI scales) were employed to measure disease severity from multiple dimensions, such as symptoms, physical signs, and quality of life.^28,29^ The occupancy ratio of the spinal canal, which evaluates the space occupied by the ossification, is moderate to highly negatively (*r* = –0.66) correlated with the JOA score (higher scores indicate better moto and sensational function) and moderately positively (*r* = 0.56) associated with NDI scores (higher scores indicate worse living quality) (Fig. S1g, h). H&E, Safranin O, and fast green (SOFG) staining of the OPLL showed that the ossification had a mature bone, haversian canal formation, and trabecular and marrow structure. Ligament tissue revealed flat nuclei cells attached to fibrous tissue. Proteoglycan-enriched cartilage and hypertrophic chondrocytes were seen at the junctional areas between OPLL and non-OPLL (Fig. S2a, b). Additionally, immunostaining demonstrated that compared with the mature ossification zone, the ligament and ossification junctional areas’ cartilage and hypertrophic areas highly expressed COLII and COLX (Fig. S2c, d), which are markers of hyaline cartilage and hypertrophic cartilage. Immunostaining of osteogenesis markers SP7, BGLAP, and RUNX2 revealed that osteogenic markers increased in the ossification areas in OPLL. In contrast, ligaments without ossification or hypertrophic cartilage in junctional areas showed a low osteogenic biomarkers expression (Fig. S2e–i). MMP1, a member of matrix metalloproteinases (MMPs) involved in the breakdown of extracellular matrix, was high in both junctional and ossified areas. (Fig. S2j, k). Altogether, our results reveal that ossifications attached to the posterior of the vertebral body exhibit histological features of endochondral ossification, causing spinal cord compression in patients with OPLL.

### Neovascularization pathways are upregulated in OPLL

**Fig. 1:**
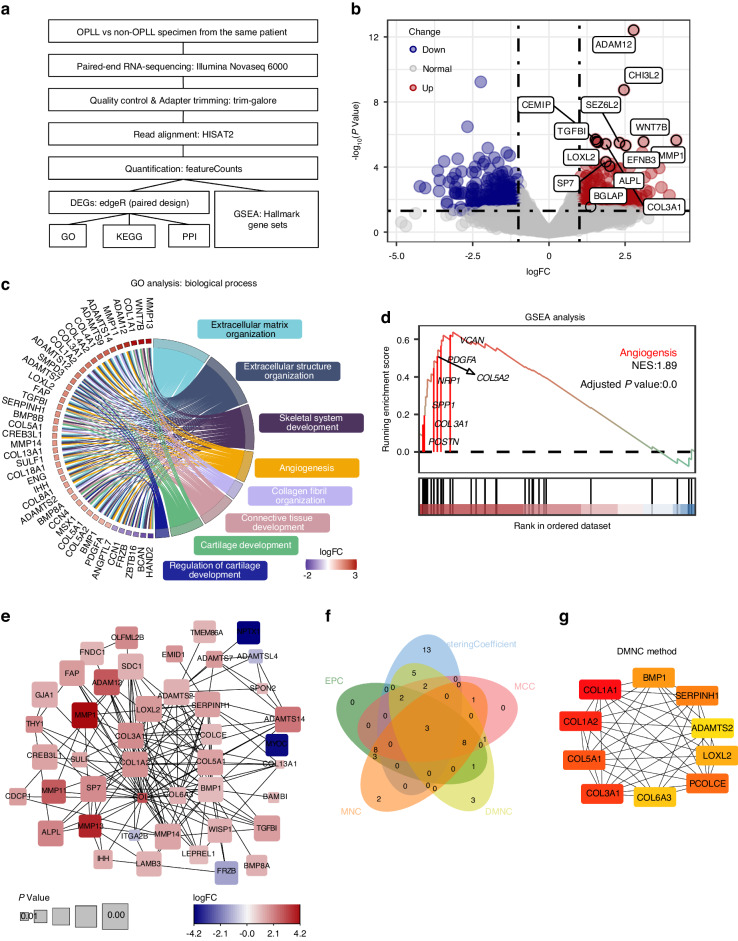
Paired samples transcriptomic sequencing reveals molecular features in OPLL samples. **a** The workflow shows the sequencing platform and the main analysis methods or software used in data processing and analysis. **b** Volcano plots exhibit differentially expressed genes (DEGs) in OPLL compared with non-OPLL samples. Red and blue dots represent significantly regulated genes (*P* < 0.05) with a fold-change higher than 2 or lower than 0.5. **c** Gene ontology (GO) analysis of DEGs. Part of significant (*P* < 0.05) GO terms (right) associated with biological processes are shown. Gene names (left) are DEGs, and the color boxes represent log_2_FC. Ribbons connect DEGs and enriched GO terms. **d** Representative enrichment plots of curated MSigDB Hallmark Gene Set Enrichment Analysis (GSEA). Adjusted *P* < 0.05 is considered statistically significant. Gene names are DEGs in the Angiogenesis pathway. **e** All protein-protein interactions (PPIs) of DEGs (*P* < 0.01) were extracted from the STRING database and generated a PPI network. The size and color of the rounded rectangular boxes indicate the *P* value and log_2_FC, respectively. **f** Venn diagram means five calculating methods for finding hub genes according to network centrality. The five methods are Maximal Clique Centrality (MCC), Density of Maximum Neighborhood Component (DMNC), Edge Percolated Component (EPC), Maximum Neighborhood Component (MNC), and ClusteringCoefficient. **g** The top 10 key genes were identified through the PPI network map using the DMNC method. Darker red indicates a higher ranking

We performed RNA-seq analysis on ligament samples from 4 OPLL patients, comparing the genome-wide changes in gene expression between ossified (OPLL) and non-ossified ligament (non-OPLL) from the same patient. The analysis flow is presented in Figure (Fig. 1a). The transcriptome analysis revealed profound changes in gene expression patterns of OPLL samples compared with non-OPLL (Fig. S3a, b). We identified 677 differentially expressed genes (DEGs), 371 upregulated genes, and 306 down-regulated genes in OPLL vs non-OPLL. We noted many statistically significant DEGs, such as *SP7*, *ALPL*, *BGLAP*, *ADAM12*, *WNT7B*, *CHI3L2*, *MMP1,* and *TGFBI* (Fig. 1b), involved in blood vessels and bone formation. MMPs are synthesized as secreted or transmembrane proenzymes and function as collagen hydrolases, causing ECM protein degradation in endochondral ossification.^30^ In addition to degrading collagen during cartilage hypertrophy, MMPs promote angiogenesis.^31^ We used the GO and KEGG pathway databases to investigate these DEGs from a functional perspective. The DEGs were significantly enriched for 113 GO terms (Table S1). Among them, the extracellular matrix organization, angiogenesis, skeletal system development, cartilage development signaling pathway in the biological process (BP) category, extracellular matrix, collagen-containing extracellular matrix in cell component (CC) category, and the enriched genes attracted our interest (Fig. 1c). An enrichment map revealed many intersections in genes enriched in ECM, bone formation and angiogenesis-related pathways (Fig. S3c), suggesting these pathways have a definite influence on the initiation and development of OPLL. The ECM-receptor interaction pathway was enriched in KEGG analysis (Fig. S3d). GSEA analysis strongly implicated angiogenesis as the primary biological process affected in ossified ligaments (GSEA normalized enrichment score = 1.89, adjusted *P* < 0.001; Fig. 1d, Table S2), and some upregulated genes enriched in this gene set were marked. The interaction network of the proteins encoded by the DEGs was constructed using the STRING database (Fig. 1e). *LOXL2*, *TGFBI,* and *PCOLCE* were in the forefront (top 25) of five analysis methods according to ranks (Fig. 1e–g, Table S3). Most of the top genes in the regulatory network are related to collagen or collagen metabolism. Moreover, *LOXL2* was enriched in pathways including ECM organization, fibril organizations, angiogenesis, skeletal system, and cartilage development (Fig. 1c). Collagen synthesis, decomposition, and assembly are closely related to osteogenesis and blood vessel formation.^32^

What is more, ligament hypertrophy is an early onset of ligament ossification.^33,34^ A microarray study (GSE113212) also showed elevated *LOXL2* levels in hypertrophy ligaments (Fig. S4a). In GSEA analysis, angiogenesis and hypoxia pathways are upregulated in hypertrophy ligaments (Fig. S4b, Table S4). The results above indicated that neovascular invasion plays a crucial role in both the early and maturation stages of OPLL. The sequencing results above indicate that the upregulation of vascular morphogenesis plays a supportive but indispensable role in OPLL, and LOXL2 may mediate it.

### Elevated LOXL2 and H-type vessels in OPLL

**Fig. 2:**
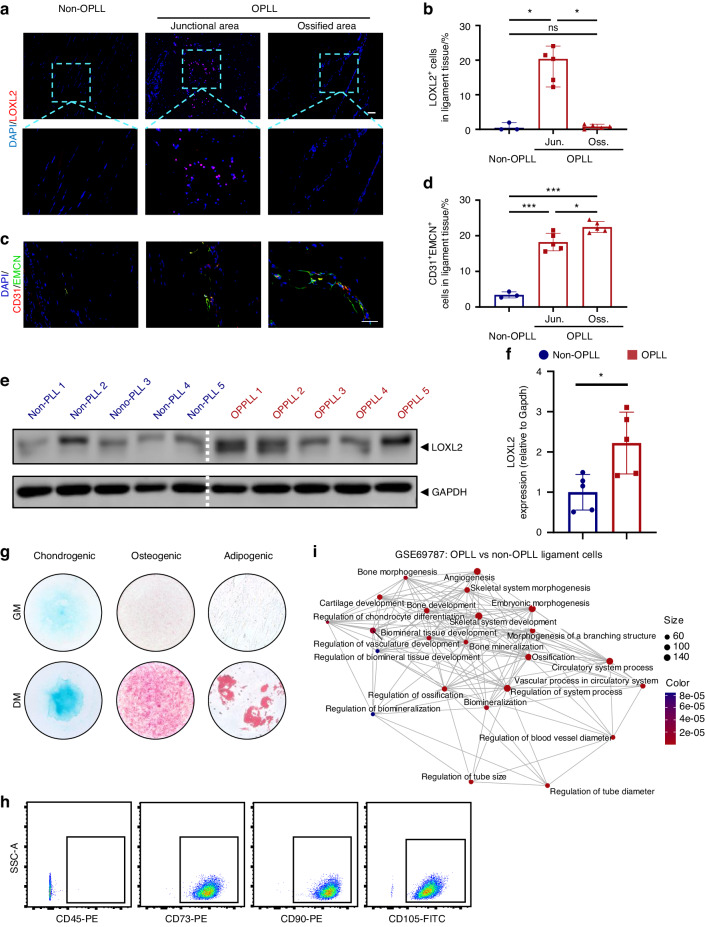
LOXL2 and blood vessels were elevated in OPLL and ligament cell characterization. **a**, **b** Representative images for IF staining (left) of LOXL2 (red) and quantification (right). Scale bar = 100 μm. Values are means ± SD. *n* = 3 for the non-OPLL group and *n* = 5 for the OPLL group, ***P < 0.001 by Student’s *t*-test. **c**, **d** Representative images for IF staining (left) of EMCN (green) and CD31 (red) of non-OPLL or OPLL samples and corresponding quantitative analysis (right). Scale bar = 100 μm. Values are means ± SD. *n* = 3 for the non-OPLL group and *n* = 5 for the OPLL group, *P < 0.05 by Student’s *t*-test. **e**, **f** Representative images of LOXL2 protein of primary culture cells derived from non-OPLL or OPLL by western blotting (left) and quantification (right). Values are means ± SD. *n* = 5, *P < 0.05 by Student’s *t*-test. **g** Representative images of trilineage differentiation of ligament cells. The left panels represent Alcian blue staining for chondrogenic differentiation; the middle panels represent ALP staining for osteogenic differentiation, and the right panels represent oil red staining for adipogenic differentiation. The upper parts represent ligament cells in the growth medium (GM), and the lower parts represent ligament cells in the differentiation medium (DM). **h** Ligament cells derived from OPLL expressed typical MSC surface markers, positive for CD73 (99.9%), CD90 (99.5%), CD105 (99.3%), and negative for CD45 (0%). *n* = 5. **i** High-throughput sequencing was performed on ligament cells derived from OPLL and non-OPLL samples (GSE69787). The GO pathway enrichment analysis unveiled significant differences in angiogenesis, bone metabolism, and signaling pathways governing vasculogenesis while identifying co-regulatory network presence. The experiments were performed in three biological replicates

**Fig. 3:**
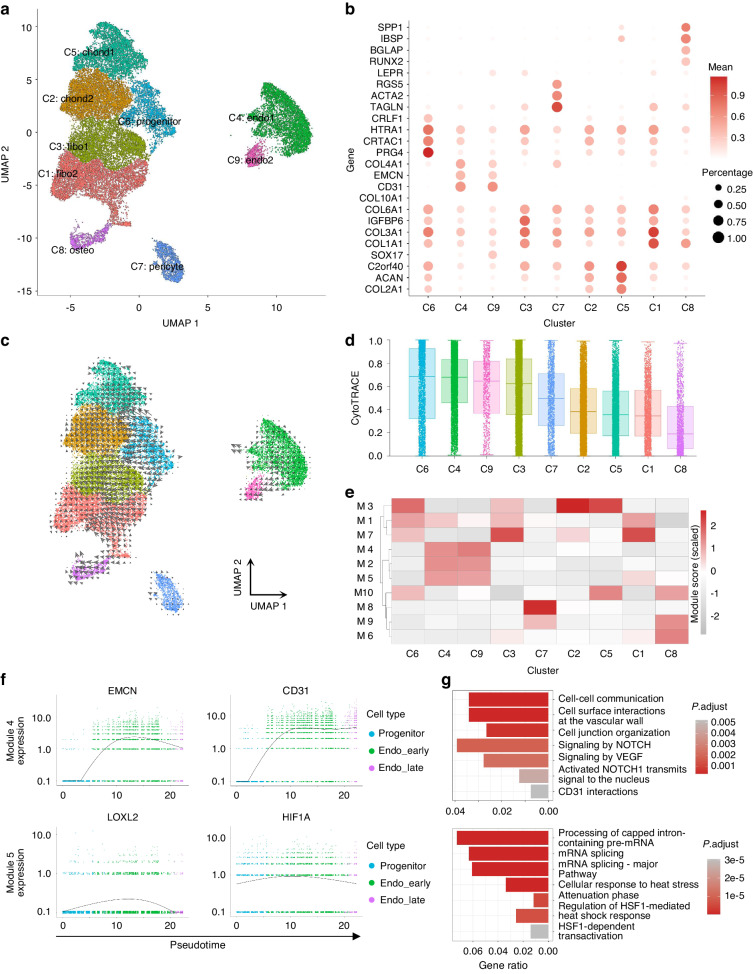
Reconstruction of multilineage differentiation trajectories in ligament cells. **a** UMAP visualization of ligament cells, endothelial cells, and pericyte subclusters. The cells were clustered into 9 distinct clusters, denoted based on their differentiation stage and cell type. **b** Dot plots illustrating marker genes specific to various cell clusters. The color bar represents the expression level of each gene, with darker colors indicating higher expression. The size of the dots corresponds to the percentage of cells expressing the respective gene. **c** UMAP embedding of RNA velocity of ligament cells indicates the transition from C6_progenitor to endothelial, cartilage, and osteoblast cells. **d** Plot displaying the cytoTRACE pseudotime order for each cluster, where the cytoTRACE score represents the differentiation stage. **e** Heatmap presenting the scaled mean expression of modules consisting of co-regulated genes, organized by Louvain community analysis across the subclusters. The color bar represents the expression level of each gene, with darker colors representing higher expression. **f** Pseudotime kinetics of the indicated genes in the modules from (**e**), showcasing their expression patterns along the trajectories towards endothelial differentiation. **g** Histogram demonstrating the enriched pathways, identified by ReactomePA, associated with each module indicated in (**e**)

In both tissues (Fig. 2a, b) and ligament cells (Fig. 2e, f) from OPLL compared to non-OPLL, LOXL2 displayed upregulation. Our study also revealed that LOXL2^+^ cells were predominantly localized in the junctional area between ossification and non-ossification (Fig. 2a). Additionally, there was an observed increase in CD31^+^EMCN^+^ type H vessel formation within the bone marrow cavity of the ossified and junctional regions (Fig. 3c, d), which was accompanied by a rise in SP7^+^ cells (Fig. S2e). Ligament cells, isolated using the explant method, have been established as playing a pivotal role in ligament ossification, a finding confirmed in prior studies.^7,10^ In another transcriptomic study (GSE69787) focusing on ligament cells isolated from OPLL and non-OPLL patients, we observed upregulation of *LOXL2* in OPLL-derived ligament cells (Fig. S4c). Furthermore, our investigations revealed that ligament cells from both OPLL and non-OPLL patients exhibit trilineage differentiation potential when cultured in osteogenic, adipogenic, and chondrogenic differentiation media (Fig. 2g). These ligament cells exhibited a characteristic spindle-shaped morphology (Fig. S4d) and displayed MSC surface markers, including negativity for CD45 and positivity for CD73, CD90, and CD105 (Fig. 2h), indicating the presence of MSC-like properties.^35^ Intriguingly, when visualizing gene sets as a network using Emapplot, we noted that many gene sets related to tube morphogenesis, tube size, and the regulation of blood vessel diameter clustered together with sets associated with blood vessel formation, ossification, bone morphogenesis, and chondrocyte differentiation (Fig. 2i, Table S5).

Considering the evidence suggesting MSCs’ potential to differentiate into blood vessels,^11,16^ we postulate that ligament cells possess the ability to differentiate into endothelial or endothelial-like cells, potentially contributing directly to vascular differentiation within the ligament. LOXL2 is suggested to play a regulatory role during the ossification process, influencing the direct differentiation of ligament cells into blood vessels.

### Single-cell sequencing reveals the endothelial differentiation trajectory of ligament cells

To determine the cellular composition of the posterior longitudinal ligament and the pseudotime trajectory of ligament cell differentiation, we performed scRNA-seq analysis on the posterior longitudinal ligament from three degenerative cervical spondylosis patients with ossified posterior longitudinal ligament (OPLL) and two patients without ossification(non-OPLL). We obtained single-cell transcriptomes from 51 245 single cells, including 30 628 cells from non-OPLL and 20 617 cells from OPLL. Unbiased clustering of the cells identified 13 clusters based on uniform manifold approximation and projection (UMAP) analyses (Fig. S5a). Each cluster was annotated based on the top principals, and the marker genes were calculated. In particular, they were as follows: 13 distinct cell clusters, including ligament cells (ligament_1–4), endothelial cells (EC_1–2), pericytes (pericyte), monocyte-macrophages (monocyte_1–2), T cells (T), B cells (B), and plasma cells (plasma_1–2). The profiles of the expression differences of the representative marker genes in the cell populations were demonstrated by statistical quantification to match the biological annotation (Fig. S5b). To study the characteristics of ligament cell vascularization in patients, we extracted ligament cells, pericytes, and endothelial cells. All these cells were clustered into nine groups (Fig. 3a). In addition to pericytes (*ACTA2*, *TAGLN*, *RGS5*) and endothelial cells (*CD31*, *EMCN*, *COL4A1*), ligament cells can be divided into 6 groups of cells, including 2 groups of ligament cells (C5: chond1 and C2: chond2) with high expression of cartilage-related markers (*COL2A1*, *ACAN*, *C2orf40*)^36^ and 2 groups of ligament cells (C3: fibo1 and C1: fibo2) with high expression of fibroblast-related markers (*COL3A1*, *COL6A1*, *IGFBP6*).^37^ There is also a cluster of ossified ligament cells (C8: osteo) with high expression of osteogenesis-related genes *(BGLAP*, *RUNX2*, *SP7*)^36^ and PRG4^+^ progenitor cells (C6: progenitor, Fig. 3b, Fig. S5c). PRG4^+^ cells are considered stem cells in heterotopic ossification.^7^ Furthermore, we conducted staining on patient tissue sections and identified PRG4^+^ cells. In non-ossified tissues, we observed regions with a high density of PRG4^+^ cells alongside areas lacking PRG4^+^ cells. Notably, nuclei in the PRG4-rich regions exhibited predominantly round shapes, which we have designated as the ‘stem cell area.’ In contrast, nuclei in areas without PRG4-positive cells displayed neatly arranged slender rods, and we have termed this region the ‘fibrous area.’ As ossification progressed, we observed a significant increase in PRG4 expression within the non-ossified junction area, while in the mature ossified region, PRG4 expression decreased (Fig. S5d, e). We also conducted fluorescence staining on the cells that migrated from the ligament tissue. We found that the cells that migrated out and were primarily cultured showed homogeneous PRG4 positivity while ACTA2 negativity (Fig. S5f, g). And in the flow cytometry analysis, ligament cells from explant migration are CD31-negative(99.6%), as well as PROCR^+^PDGFRA^+^ (98.2%, Fig. S5h), frequently mentioned signature genes associated with progenitor cells.^36^ Based on the previous flow cytometry analysis (Fig. S5h), fluorescent staining results (Fig. S5f, g), and the three-lineage differentiation ability of ligament cells (Fig. 2g), we believe that the ligament cells used represent the C6 progenitor, as shown by single-cell sequencing(Fig. S5c, Fig. 3a, b).

To better understand the characteristics and cellular hierarchy in ligament cells in OPLL, we initially examined scRNA-seq data using UMAP embedding of RNA velocity. The dynamic trajectories revealed a transition from C6 (progenitor cells) to C4, C9 (endothelial cells), C2, C5 (chondrocytes), and C8 (osteoblasts) (Fig. 3c). Besides, C6 cells were estimated as undifferentiated by CytoTRACE scores (Fig. 3d).

Therefore, we designated C6: progenitor as the starting point for differentiation trajectories and utilized Monocle3 to construct ligament-to-vascular differentiation trajectories. To explore gene expression dynamics along the trajectories, we grouped genes that varied between cell clusters into 10 modules using Louvain community analysis. A heatmap showed the aggregated expression in each module across cell clusters (Fig. 3e). We found that the expression of module 4 and module 5 gradually increased with the differentiation of ligament cells into endothelial cells. Endothelial cell-specific markers are located in module 4, such as *PECAM1* and *EMCN* (Fig. 3f), and the signal pathways enriched in module 4 are mainly related to vascular morphogenesis and VEGF signaling pathways (Fig. 3g). While *LOXL2* and *HIF1A* (Fig. 3f) are situated within module 5, the enriched signaling pathways primarily pertain to RNA splicing and the heat shock response (Fig. 3g). Interestingly, both *LOXL2* and *HIF1A* exhibited a pattern of initial increase followed by a subsequent decrease during the process of endothelial differentiation (Fig. 3f). We constructed the hypothetical trajectory of cell differentiation evolution within ligament tissue and constructed the trajectory of ligament cell differentiation into blood vessels in silico.

### Ligament cells can differentiate into endothelial-like cells

**Fig. 4:**
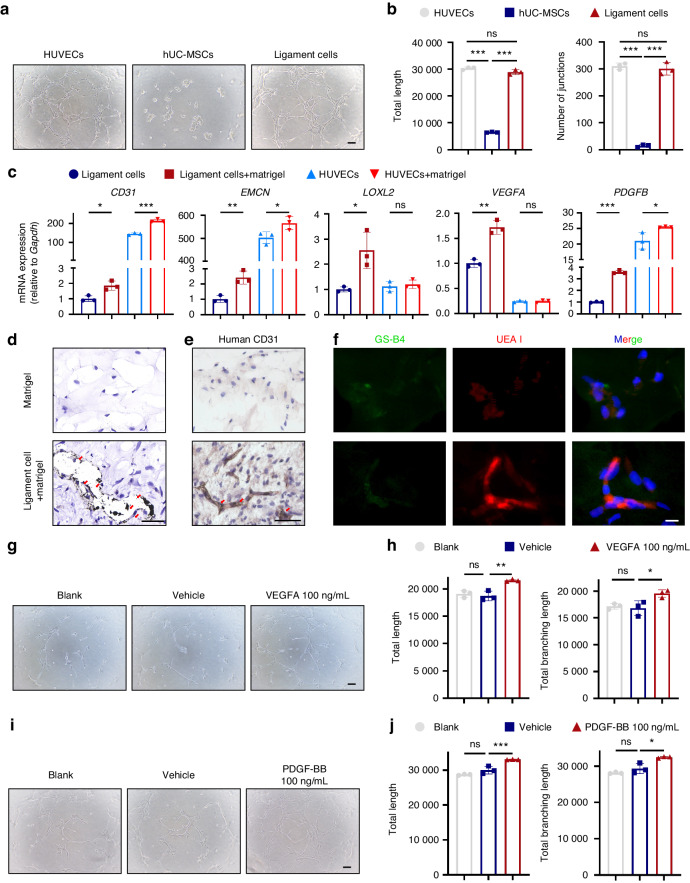
Ligament cells can acquire endothelial-like phenotype and are regulated by VEGFA and PDGF-BB. **a**, **b** Representative images of capillary-like structures of human umbilical vein endothelial cells (HUVECs), Human Umbilical Cord Mesenchymal Stem Cells (hUC-MSCs) and Ligament Cells on Matrigel (left) and quantification (right). Scale bar = 100 μm. Values are means ± SD (*n* = 3). ***P < 0.001, ns not significant by ANOVA. **c** Changes in the expression level of specified genes in both ligament cells and HUVECs after undergoing endothelial differentiation on Matrigel. Values are means ± SD *(n* = 3). *P < 0.05, **P < 0.01, ns not significant by Student’s *t*-test. **d** Sections of Matrigel spheres loaded or unloaded with ligament cells were stained with H&E. Due to prior ink perfusion through the left ventricle, the deposition of black particles indicated the formation of functional blood vessels. The red arrows indicate the deposition of ink particles (*n* = 6). Scale bar = 50 μm. **e** The CD31 immunohistochemistry results on sections of Matrigel spheres reveal black particle deposition on the inner side of the CD31-positive area. The CD31 antibody used in this experiment specifically reacts with humans. The red arrows indicate the deposition of ink particles *(n* = 6). Scale bar = 50 μm. **f** Representative images for UEA I (red) and GS-B4 (green) staining of Matrigel spheres loaded or unloaded with OPLL-derived ligament cells *(n* = 6). Scale bar = 10 μm. **g**, **h** Representative images depicting capillary-like structures formed by OPLL-derived ligament cells on Matrigel under various conditions (left): unstimulated (Blank), PBS-treated (Vehicle), and stimulated with VEGFA (100 ng/mL); accompanied by quantitative analysis (right). Scale bar = 100 μm. Values are means  ±  SD (*n* = 3). *P < 0.05, **P < 0.01, ns not significant by ANOVA. **i**, **j** Representative images display capillary-like structures formed by OPLL-derived ligament cells on Matrigel under distinct conditions (left): unstimulated (Blank), ddH_2_O (Vehicle), and PDGF-BB (100 ng/mL), including quantification (right). Scale bar = 100 μm. Values are means ± SD (*n* = 3). *P < 0.05, **P < 0.01, ns not significant by ANOVA. The experiments were performed in three biological replicates

To explore the potential role of ligament cells in vascularization during the initiation and progression of OPLL, we performed an in vitro capillary formation assessment using a Matrigel assay. This evaluation included three cell types: human umbilical vein endothelial cells (HUVECs) as a positive control, human umbilical cord mesenchymal stem cells (hUC-MSCs), and ligament cells. Additionally, our goal was to identify any differences in endothelial differentiation capacities between ligament cells and MSCs, given the previously observed MSC-like properties of ligament cells. HUVECs exhibit a cobblestone-like appearance, whereas hUC-MSCs and ligament cells display a fibroblast-like spindle shape (Fig. S6a). HUVEC and ligament cells formed capillary-like structures on the Matrigel without stimulation, displaying similar characteristics (Fig. 4a, b). In contrast, hUC-MSCs did not form capillary-like structures; instead, these cells exhibited a tendency to cluster together, generating small cellular nodules (Fig. 4a). Moreover, despite their shared classification, the distinct behaviors of ligament cells and hUC-MSCs highlight the heterogeneity within the MSC category. During the tube formation process, we noted an increased expression of endothelial markers. HUVECs showed a higher expression of these markers, about 100-fold greater than ligament cells. Simultaneously, ligament cells exhibited increased *LOXL2*, *VEGFA*, and *PDGFB* expression, not seen in HUVECs except for *PDGFB* (Fig. 4c). This differential expression reveals distinct molecular features of each cell type. Interestingly, ligament cells displayed upregulated osteogenic marker *SP7* during endothelial differentiation, a phenomenon absent in HUVECs. Also, the transcription factor *RUNX2*, associated with *SP7*, showed increased expression in both HUVECs and ligament cells (Fig. S6b). These observations may suggest a close relationship between vascularization and ossification in ligament cells, implying a high degree of coupling between these processes. We subsequently performed an in vivo vascular network formation assay to investigate whether ligament cells could adopt an endothelial-like phenotype and establish a vascular network structure in vivo.^38,39^ Briefly, we subcutaneously injected Matrigel, either loaded or unloaded with human ligament cells into nude mice, forming gel bulges. We additionally conducted flow cytometry analysis on CD31 (Fig. S5h) and performed immunofluorescence staining of ACTA2 (Fig. S5g) on subcutaneously injected ligament cells, aiming to exclude any endothelial or pericyte contamination. Before sample collection, ink was perfused through the left ventricle, and ink particles within the gel spheres indicated the successful formation of functional blood vessels.^40^ Notably, gel spheres containing a combination of ligament cells and Matrigel exhibited black ink particle deposition in the tissue sections. In contrast, the control group that received Matrigel alone showed no ink particle deposition (Fig. 4d, Fig. S6c). To further confirm the origin of the formed blood vessels, we conducted immunohistochemical staining for human CD31 on Matrigel sphere sections. The antibody used specifically reacts with human CD31 and the specificity was rigorously validated using murine kidney tissue as a negative control and human renal glomeruli from adjacent tissues of renal cell carcinoma as a positive control (Fig. S6d). The staining results indicated the deposition of black particles on the inner side of the CD31-positive area (Fig. 4e). These findings imply that ligament cells, derived from humans, not only exhibit the expression of endothelial markers within a murine model, but also demonstrate the capability to establish a functional vascular network structure. Also, staining was performed on the tissue sections using Ulex Europaeus Agglutinin I (UEA I) in red, which specifically targets human endothelium, and Griffonia Simplicifolia isolectin B4 (GS-B4) in green, which binds to mouse endothelium but does not react with human cells due to the absence of the α-linked galactose residue required for GS-B4 carbohydrate binding.^38,39^ In the gel spheres loaded with ligament cells, distinct UEA I-positive regions were observed. At the same time, GS-B4 staining showed negative results (Fig. 4f). This provides compelling evidence that the differentiated ligament cells were responsible for forming functional blood vessels. Adding 100 ng/mL VEGFA or PDGF-BB in ligament cells promoted capillary-like structure formation (Fig. 4g–j). In the absence of Matrigel, VEGFA influenced cell shape, causing ligament cells to adopt an endothelial-like phenotype and exhibit higher expression of *CD31* and *EMCN* (Fig. S6e, f). Adding the PDGFR inhibitor imatinib (Fig. S6g, h) or VEGFA monoclonal antibody, Bevacizumab inhibited the formation of capillary-like structures (Fig. S6i, j). Considering all results, it is evident that ligament cells can differentiate into endothelial-like cells and have the potential to participate in vascularization in OPLL. PDGF-BB and VEGFA may play crucial roles in the initiation and progression of OPLL during this process. Further investigation is warranted to elucidate the underlying mechanisms and the role of LOXL2.

### LOXL2 modulates ligament cell endothelial-like differentiation through the HIF1A pathway

**Fig. 5:**
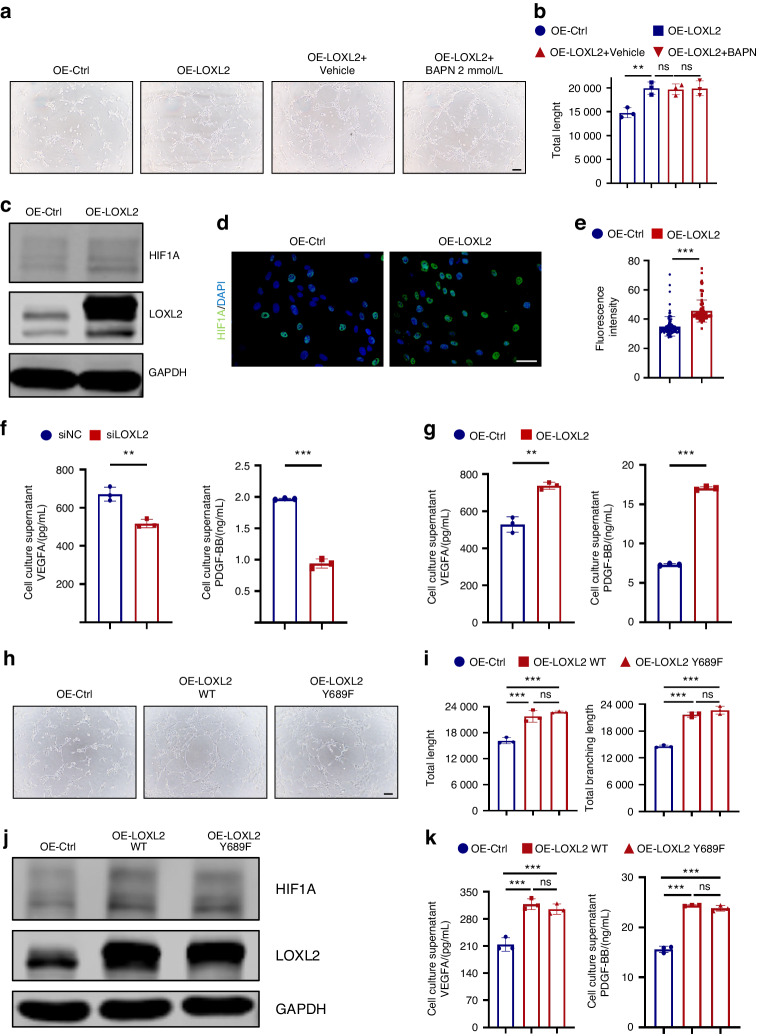
LOXL2 modulates ligament cell endothelial-like differentiation through HIF1A signaling pathway. **a**, **b** Increased expression of LOXL2 can enhance vasculogenesis, and BAPN did not inhibit the enhancement caused by overexpression of LOXL2. Ligament cells were transfected with LOXL2 plasmids (OE-LOXL2) or empty vector plasmids (OE-Ctrl), then treated with 2 mmol/L BAPN or PBS (Vehicle). Scale bars = 100 μm. Values are mean ± SD (*n* = 3) **P < 0.01, ns not significant by ANOVA. **c** Representative images of LOXL2 and HIF1A protein of ligament cells by western blotting. Ligament cells were treated with or without LOXL2 plasmids. **d**, **e** Representative immunofluorescence staining of HIF1A and quantification by fluorescence intensity. Ligament cells were transfected with or without LOXL2 plasmids. Scale bars = 50 μm. *n* = 3 and 100 cells were randomly selected. ***P < 0.001 by Student’s *t*-test. **f**, **g** Cell culture supernatant VEGFA and PDGF-BB production was measured via ELISA. After the knockdown or overexpression of LOXL2 for 24 h, medium supernatants of ligament cells were harvested to measure VEGFA and PDGF-BB concentrations. Values are mean ± SD (*n* = 3). **P < 0.01, ***P < 0.001 by Student’s *t*-test respectively. **h**, **i** Overexpression of wild-type LOXL2 (OE-LOXL2 WT) or a point-mutated LOXL2 variant (OE-LOXL2 Y689F), characterized by the absence of enzymatic activity, in ligament cells elicits enhancement in ligament cell vasculogenesis, as demonstrated through quantitative analysis. Scale bars = 100 μm. The presented data includes mean values ± SD (*n* = 3). Statistical significance was determined by ANOVA, with asterisks indicating significance (**P < 0.001) and “ns” denoting non-significance. **j** Protein expression levels of LOXL2 and HIF1A within ligament cells were assessed through western blotting. GAPDH served as a loading control. Ligament cells were subjected to treatment with either wild-type or point-mutated LOXL2 variants. **k** Quantification of VEGFA and PDGF-BB production in the cell culture supernatant was achieved via ELISA. Following a 24-h overexpression of wild-type LOXL2 or the point-mutated LOXL2 variant, medium supernatants from ligament cells were collected to measure VEGFA and PDGF-BB concentrations. Data is presented as mean values ± SD (*n* = 3). Statistical significance was determined by ANOVA, with triple asterisks indicating high significance (***P < 0.001) and “not significant” denoting the absence of significance. All experiments were conducted with three independent biological replicates

We observed an increase in the formation of capillary structures following the overexpression (OE) of LOXL2 (Fig. 5a, b). BAPN, known to inhibit pan-Lox(L)-catalyzed oxidation of lysine residues on collagen with an IC50 of ~6 μmol/L,^41^ was administered at a concentration of 2 mmol/L. However, we observed that this treatment with BAPN did not impact the formation of tube-like structures in ligament cells (Fig. 5a, b). The microarray results and enrichment analysis revealed an upregulation of hypoxia and angiogenesis pathways in hypertrophied and ossified ligament tissues (Fig. 1d, Fig. S4b). Notably, the transcription factor HIF1A, recognized as a master regulator of hypoxic signaling, exerts strong control over angiogenesis and metabolic reprogramming through nuclear translocation in various pathological and physiological contexts.^42,43^ We hypothesized that LOXL2 might exert its regulatory influence on ligament cells, facilitating blood vessel formation through the modulation of HIF1A expression. Indeed, we observed a significant upregulation of HIF1A in ligament cells following the overexpression of LOXL2 (OE-LOXL2) (Fig. 5c–e). Furthermore, the overexpression of LOXL2 led to increased expression of *HIF1A* and its downstream genes, *VEGFA-C*, and *PDGFB* (Fig. S7h).^12^ Conversely, the knockdown of LOXL2 reduced the formation of capillary structures, and the inhibition of LOXL2 by BAPN did not affect tube formation (Fig. S7a–c). Additionally, the knockdown of LOXL2 resulted in the downregulation of *HIF1A, VEGFA-C, and PDGFB* at the transcriptional level (Fig. S7d–g). We also quantified VEGFA and PDGFBB concentrations in the culture medium supernatants using ELISA. Notably, the medium supernatants from knockdown LOXL2 cells exhibited lower concentrations of VEGFA and PDGF-BB (Fig. 5f), whereas the medium supernatants from LOXL2 overexpression displayed higher concentrations (Fig. 5g). As previously observed, the enzymatic activity of LOXL2 does not affect LOXL2’s regulation of ligament cell vascularization. To further confirm this, we constructed a LOXL2 point mutation plasmid (OE-LOXL2 Y689F) to abolish its enzymatic activity.^44,45^ Importantly, this plasmid, despite lacking enzymatic activity, still promoted tube formation in ligament cells (Fig. 5h, i) and enhanced HIF1A expression, along with the secretion of VEGFA and PDGF-BB (Fig. 5j, k). Then, we constructed a co-culture system of Human Umbilical Vein Endothelial Cells (HUVECs) and the supernatant of ligament cells after the knockdown or overexpression of LOXL2 to perform transwell migration assays. The medium supernatant from the knockdown of LOXL2 can reduce HUVECs migration (Fig. S7i, j), and the medium supernatant overexpressing LOXL2 can promote HUVECs migration (Fig. S5k, l). Based on the results mentioned above, it can be deduced that LOXL2 orchestrates both the endothelial-like differentiation of ligament cells and the migration of endothelial cells. We also measured serum VEGFA concentrations of OPLL and non-OPLL patients. We compared the baseline characteristics of the two groups of patients. There are no statistical differences in gender, age, comorbidities, and menstrual status in female patients; the OPLL had a higher NDI score and lower JOA score, which means the OPLL patients’ symptoms are more severer than non-OPLL patients (Table S6). We found that the serum VEGFA level was higher in OPLL patients, and an ROC curve was generated. From the ROC curve, the area under the ROC curve is 0.715 (Fig. S7m, *P* = 0.01), and a sensitivity of 60% and specificity of 85.71% at a cut-off probability of 21.88 pg/mL, suggesting the serum VEGFA has a potential to be a biomarker for the diagnosis of OPLL. Most OPLL patients and controls had plasma LOXL2 concentrations below measurable thresholds (data not shown). Furthermore, it came to our attention that the use of a selective LOXL2 enzyme activity inhibitor, specifically (2-Chloropyridin-4-yl)methanamine hydrochloride at a concentration of 126 nmol/L, did not exhibit inhibitory effects on the osteogenic differentiation of ligament cells. This was determined through Alizarin Red staining (Fig. S8a, b) and the assessment of *RUNX2* and *BGLAP* mRNA levels (Fig. S8c). The results demonstrate that LOXL2 affects the ligament cells’ endothelial differentiation by regulating the HIF1A signaling pathway.

### Sorafenib attenuates the progression of BMP-induced ossification

**Fig. 6:**
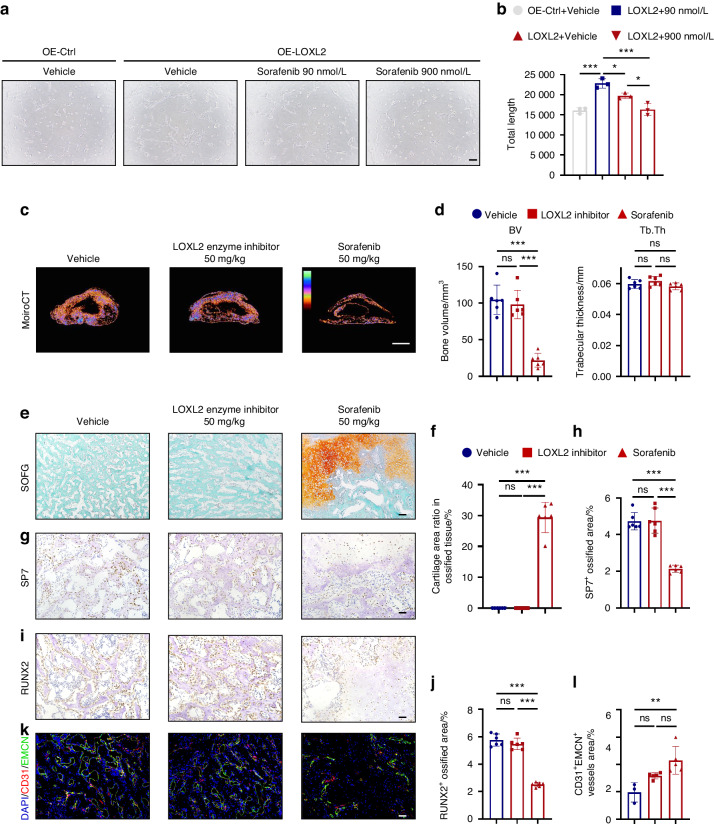
Sorafenib attenuates the progression of the BMP-induced In vivo Ossification (BIO) model. **a**, **b** Representative pictures of capillary-like structures formed on Matrigel (left) of ligament cells overexpressing empty vector plasmids (OE-Ctrl) and LOXL2 plasmids (OE-LOXL2) with the treatment of sorafenib (90 nmol/L and 900 nmol/L) or DMSO (Vehicle); alongside quantification (right). Scale bars = 100 μm. Values are means ± SD (*n* = 3). ***P < 0.001, *P < 0.005 by ANOVA. **c**, **d** Representative reconstructed 3D micro-CT transverse position images depict the ossification site following a 2-week treatment with 50 mg/kg Sorafenib, an LOXL2 enzyme inhibitor [(2-Chloropyridin-4-yl)methanamine hydrochloride] and a methylcellulose carrier alone (Vehicle). Colored bars within the images signify the density of ossified tissue, with higher density indicated at the top and lower density at the bottom. Quantitative analysis of structural parameters related to heterotopic bone formation, including Bone Volume (BV) and Trabecular Thickness (Tb.th), is presented. Scale bar = 2 mm. Data is reported as mean values ± SD (*n* = 6). Statistical analysis was performed using ANOVA, with triple asterisks indicating high significance (***P < 0.01), and “ns” denoting non-significance. **e**, **f** Safranin O-Fast Green (SOFG) staining of sections from the ossification site following a 2-week treatment period, accompanied by the quantification of the cartilage area ratio within the ossified tissue. The scale bar represents 100 μm. Data is presented as means ± SD (*n* = 6). Statistical analysis was conducted using ANOVA, with triple asterisks indicating high significance (***P < 0.001) and “ns” signifying non-significance. **g**–**j** The left panel displays representative images depicting the presence of SP7 and RUNX2 within the ossification site, while the right panel presents quantitative analysis. The scale bar corresponds to 50 μm. Error bars represent mean values ± SD (*n* = 6). Statistical significance was determined by ANOVA, with ***P < 0.001 and “ns” indicating non-significance. **k**, **l** Representative images demonstrate immunofluorescence (IF) staining for EMCN (green) and CD31 (red) within the ossification site, followed by the corresponding quantitative analysis (right). The scale bar represents 50 μm. Data is reported as means ± SD (*n* = 6). Statistical analysis was performed using ANOVA, with double asterisks indicating significance (**P < 0.01) and “ns” indicating non-significance. All experiments were conducted with three independent biological replicates

Based on our previous findings that LOXL2 regulates vascularization through the HIF1A/VEGFA pathway, we hypothesized that VEGFA monoclonal antibodies or VEGFR inhibitor might inhibit this phenomenon. Therefore, we conducted experiments to evaluate the VEGFA monoclonal antibody and the VEGFR inhibitor sorafenib on LOXL2-induced vascularization in ligament cells. Our results revealed that both Bevacizumab and sorafenib could markedly suppress the upregulation of endothelial-like differentiation mediated by LOXL2 (Fig. S9a, b, Fig. 6a, b). Sorafenib can simultaneously inhibit VEGFR and PDGFR as a multi-targeted tyrosine kinase inhibitor.^46^ Since both VEGFA and PDGF-BB can promote the endothelial-like differentiation of ligament cells, we intend to conduct experiments using sorafenib to study its potential effect on inhibiting vascular morphogenesis and subsequently inhibiting the progression of ossification. Scaffolds carrying rhBMP-2 were implanted subcutaneously on the back of C57 BL/6J mice to generate ossification (BMP-induced In vivo Ossification, BIO). We conducted oral administrations of the LOXL2 enzyme inhibitor [(2-Chloropyridin-4-yl)methanamine hydrochloride] or Sorafenib at a dose of 50 mg/kg to assess their effectiveness in hindering the progression of ossification. We used Micro-CT to detect bone parameters after 14 days of treatment. The sorafenib intervention group exhibited significant inhibition of ossification progression, whereas the inhibition of LOXL2 enzyme activity did not delay the ossification progression (Fig. 6c and Fig. S9c). When sorafenib was administered, there was a significant decrease in bone volume (BV), total volume (TV), and bone surface (BS). Moreover, all groups exhibited almost no change in trabecular number (Tb. N) and trabecular thickness (Tb. th) (Fig. 6d and Fig. S9d). SOFG staining revealed dense trabecular bone structures and mature bone tissue formation in the vehicle and LOXL2 enzyme inhibitor group samples. In contrast, the samples from the sorafenib treatment group exhibited sparse trabecular bone structures, accompanied by numerous cartilage structures and amorphous matrix deposition, indicating a delay in endochondral ossification (Fig. 6e, f). Immunohistochemical and immunofluorescent analyses demonstrated that sorafenib-treated samples exhibited significantly lower levels of SP7 and RUNX2 compared to the vehicle group (Fig. 6g–j). Additionally, sorafenib had a suppressive effect on CD31^+^EMCN^+^ type H vessels (Fig. 6k, l). We also evaluated the potential side effects associated with sorafenib and LOXL2-selective inhibitors. While sorafenib effectively inhibited the progression of ossification, it had no discernible impact on the spinal bone mass and structure of mice compared with the control group (Fig. S9e, f). Additionally, we employed Masson staining and Sirius red staining to assess the liver and kidney structures in mice from different treatment groups, and our findings indicated that neither sorafenib nor the LOXL2 selective inhibitor had any adverse effects on the liver and kidney structures (Fig. S9g). Consistent with our previous findings, decoupling of blood vessel formation and osteogenesis caused by sorafenib can hinder the progression of ossification.

### Sorafenib can inhibit LOXL2-induced ligament cell osteogenic differentiation

The involvement of osteogenic differentiation of ligament cells is also crucial in the progression of posterior longitudinal ligament ossification. Previous studies have indicated that ligament cells derived from patients with posterior longitudinal ligament ossification exhibit a higher capacity for osteogenic differentiation.^47^ To investigate whether sorafenib could inhibit the osteogenic differentiation of ligament cells, we treated the cells with 90 and 900 nmol/L sorafenib, which significantly inhibited osteogenic differentiation as shown by the reduced expression of osteogenesis-related genes and decreased ALP staining (Fig. S10a, b). We also conducted further experiments using lentivirus-mediated (LV) overexpression of LOXL2 (LV-LOXL2) or Vector (LV-Vector) in ligament cells in the presence or absence of sorafenib. Our results showed that overexpression of LOXL2 promoted osteogenic differentiation of ligament cells, which could be reversed by 900 nmol/L sorafenib (Fig. S10c, d). Osteogenesis-related genes exhibited similar results consistent with Alizarin Red staining (Fig. S10e). In summary, our findings suggest that upregulation of LOXL2 can enhance osteogenic differentiation and tube formation of ligament cells. At the same time, sorafenib can reverse the enhanced osteogenesis and tube formation caused by LOXL2 upregulation.

### Sorafenib can inhibit ossification and relieve neurological symptoms

**Fig. 7:**
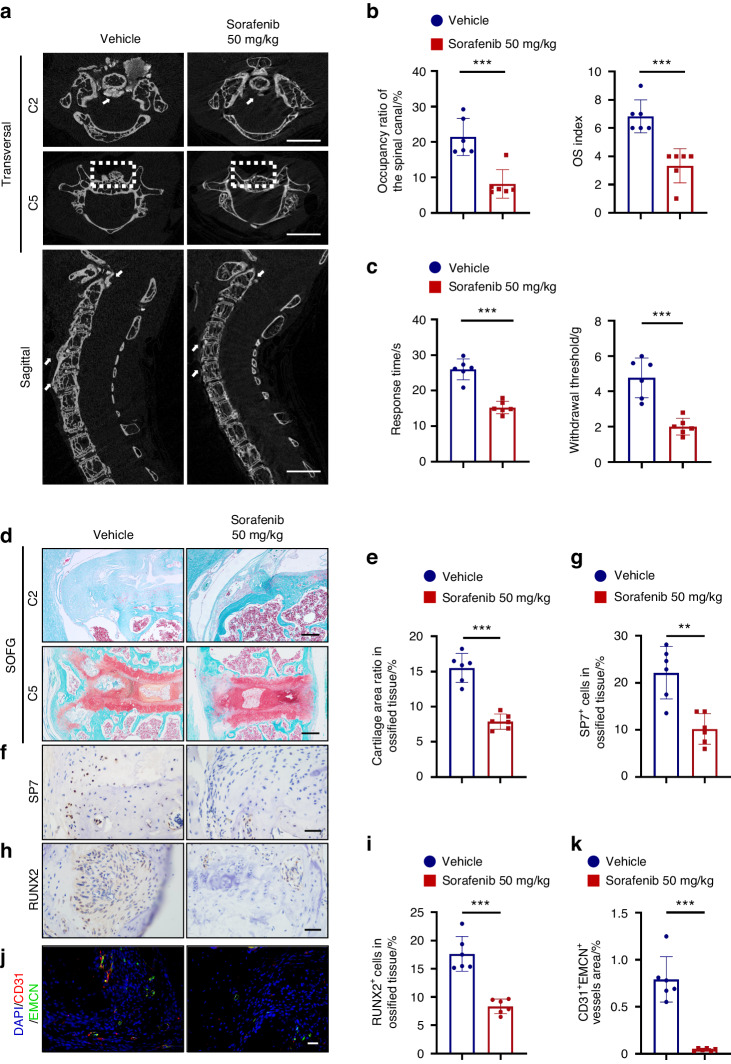
Sorafenib can inhibit ossification and improve neurological symptoms in Ligament Spontaneous Ossification (LSO) model. **a** Representative 3D reconstructed Micro-CT images of the cervical spine. Following 14 weeks of intragastric administration of sorafenib or vehicle, the cervical spine of the mice was scanned and reconstructed in transverse (upper) and sagittal (lower) positions. This evaluation aimed to assess the occupation of ossified ligament tissue within the spinal canal of the LSO model. Ossified ligaments are indicated by white arrows and dotted boxes. Scale bars = 2 mm. **b** Histograms displaying the occupancy ratio of the spinal canal and the Ossification (OS) index of the cervical spine. Data is presented as means ± SD (*n* = 6). Statistical significance was assessed using Student’s *t*-test (***P < 0.001). **c** Von Frey assay (left) and thermal hyperalgesia test (right) were conducted on the right hind paw of mice treated with vehicle or 50 mg/kg sorafenib at 18 weeks. Error bars represent mean values ± SD (*n* = 6). Statistical significance was determined using Student’s *t*-test (***P < 0.001). **d**, **e** Representative safranin O and Fast Green (SOFG) staining images of the C2 (upper) and C5 (lower) cervical spine sections treated with or without sorafenib, accompanied by the quantification of the cartilage area ratio within the ossified tissue. Scale bars =100 μm, *n* = 6. Statistical significance was determined using Student’s *t*-test (***P < 0.001). **f**–**i** Immunostaining of cervical spine sections from each group with indicated antibodies, along with quantitative analysis. Scale bar = 40 μm. Error bars represent mean values ± SD (*n* = 6). Statistical significance was assessed using Student’s *t*-test (**P < 0.01, ***P < 0.001). **j**, **k** Representative immunofluorescence images illustrating EMCN (green) and CD31 (red) staining within the ossified ligament of mice treated with vehicle or sorafenib, accompanied by corresponding quantitative analysis. Scale bar = 50 μm. Error bars represent mean values ± SD (*n* = 6). Statistical significance was determined using Student’s *t*-test (***P < 0.01). All experiments were conducted with three independent biological replicates

To study whether sorafenib can inhibit ossification progression in vivo, we took advantage of a widely used spontaneous ossification model of posterior longitudinal ligament caused by *enpp1* knockout (Fig. S11a, Ligament Spontaneous Ossification, LSO).^3,10,48^ Compared with their littermates, the LSO mice showed severe spinal ligament ossification (Fig. S11b) and the endochondral ossification process in SOFG staining (Fig. S11c). All the characteristics were similar to OPLL patients, and LOXL2 was highly expressed at the ossification sites (Fig. S11d). We performed intragastric administration of 50 mg/kg of sorafenib or vehicle starting at 4 weeks of age. Micro-CT was used to analyze the presence of ligament ossification and the occupancy ratio of the ossified mass in the spinal canal. From both transversal and sagittal view, we found that 50 mg/kg of sorafenib can reduce the osteophytes of the spinal ligament in multiple spinal segments (Fig. 7a). The ossified mass occupancy ratio of the spinal canal and OS index was less than the vehicle treatment (Fig. 7b). The two parameters both mean the ossification was inhibited. We also performed mechanical and thermal stimulation tests to assess the mice’s sensory and motor function. We found a shorter response time to thermal stimulation and less paw withdrawal threshold to mechanical stimulation when sorafenib was administrated (Fig. 7c). The result above suggested that the neurological symptoms could be relieved by reducing spinal ligament ossification when treated with 50 mg/kg of sorafenib. We observed that the cartilage and ossification area of mice’s spinal ligament decreased in SOFG staining (Fig. 7d, e). Histochemistry analysis showed sorafenib inhibited the expression of osteogenic marker RUNX2 and its downstream transcription factor SP7 (Fig. 7f–i). Also, the CD31^+^ and EMCN^+^ type H vessel formation in the ossified region was significantly suppressed by sorafenib (Fig. 7j, k). All these results demonstrate that 50 mg/kg of sorafenib can also significantly inhibit the spinal ligament ossification progression of the LSO model by breaking the neovascularization and osteogenesis coupling in vivo.

## Discussion

In conclusion, this investigation reveals the inherent vasculogenesis capability of ligament cells and their potential to form functional blood vessel-like structures in the OPLL microenvironment. We show that LOXL2 modulates the endothelial-like differentiation of ligament cells via HIF1A signaling, thus promoting the secretion of VEGFA and PDGF-BB. By blocking VEGFR and PDGFR, an FDA-approved sorafenib can significantly inhibit ligament cell endothelial-like differentiation induced by LOXL2 activation. Sorafenib also significantly suppressed ossification progression and improved function in various ossification animal models. Our findings underscore the potential of LOXL2 as a promising therapeutic target to disrupt the coupling of vascularization and osteogenesis. Furthermore, our study suggests that sorafenib holds clinical promise as a long-term inhibitor for OPLL.

OPLL can be divided into two categories according to its etiology: primary or secondary (syndromic) OPLL. The latter often results from genetic defects, such as hypophosphatemia, rickets/osteomalacia, hypoparathyroidism, and acromegaly/gigantism.^49^ Primary OPLL is a localized hyperostosis of the posterior longitudinal ligament. The early stage of OPLL is usually asymptomatic. When the ossified ligament compresses the spinal cord or nerves, it can cause motor and sensory symptoms. Although many genetic and environmental factors have been reported to be associated with the development of OPLL,^9^ the understanding of OPLL occurrence and progression mechanism is still insufficient. As a result, symptomatic OPLL can only be treated by surgery, and non-surgical treatment can only relieve symptoms.^50^ It is also noted that spinal cord injury patients with OPLL have more severe preoperative motor dysfunction and poorer prognosis.^51^ To our knowledge, surgical intervention rather than conservative treatment is the best treatment for severe OPLL patients to restore spinal canal volume and relieve compression.^52^ However, surgical treatment cannot wholly remove the ossification and has many complications.^53^ Furthermore, surgery cannot solve the problem of the continued growth of ossification. Worse, some posterior cervical surgery methods (Laminoplasty) may even accelerate the growth of ossification.^54^ It will be an optimal solution to treat OPLL precisely based on the pathogenesis.

Our study utilized bulk and single-cell transcriptomic sequencing to investigate the molecular features of OPLL. In addition to the osteogenesis-related process, we also observed activation in vascular morphogenesis pathways in ossified tissues.

PRG4 is widely recognized as a chondroprogenitor marker.^55^ In our investigation, we’ve made an intriguing discovery – PRG4^+^ ligament stem cells possess a remarkable differentiation potential, spanning across endothelial, osteogenic, and chondrogenic lineages. This multifaceted capability underscores their significance in the context of OPLL. Notably, previous studies have shed light on the crucial role of PRG4^+^ Tendon Stem/Progenitor Cells in the ectopic ossification of Achilles tendons in mice,^7^ reinforcing the relevance of our findings.

It’s important to note that our methodology for obtaining primary ligament cells diverges from the typical enzymatic digestion approach. Instead, we isolate ligament cells through tissue adhesion. This unique approach yields primary cultured cells that exhibit remarkable homogeneity, a characteristic we’ve rigorously assessed through flow cytometry and cell immunofluorescence (PRG4^+^). When combined with Matrigel and injected into nude mice, these ligament cells can form functional human blood vessels. The in vivo result, complemented by in vitro Matrigel experiments, underscores the capacity of ligament cells for blood vessel formation. Moreover, adding exogenous PDGF-BB and VEGFA can enhance this process, while inhibitors or monoclonal antibodies against PDGFR and VEGFR can inhibit it. Combining these findings with sequencing data on ligament cells, we hypothesize that ligament cells may contribute to the initiation and progression of OPLL by directly generating blood vessels. The osteogenesis and vascular morphogenesis processes are highly coupled for bone formation, suggesting that inhibiting blood vessel formation may achieve the goal of inhibiting ossification.

LOXL2 plays a positive role in regulating ligament endothelial-like differentiation in OPLL, and both loss- and gain-of-function proved that LOXL2 regulates HIF1A at the transcription level. We saw a corresponding change in VEGFA and PDGFB levels along with LOXL2 knockdown and overexpression. Interestingly, our study found that inhibiting the enzyme activity did not affect the capillary-like structure formation, consistent with previous findings.^20^ In research on the hepatocellular carcinoma cell line, overexpression of wild-type LOXL2 can increase HIF1A expression level. However, overexpression of the point-mutated form of LOXL2, which lacks enzymatic activity, could also upregulate HIF1A and angiogenesis. Also, the LOXL2 antibody showed no effect on Snail, an upstream transcription factor of HIF1A.^20,45^ All the findings above may explain why LOXL2 is highly expressed in many fibrotic diseases, but LOXL2 monoclonal antibody simtuzumab has widely failed in clinical intervention studies of many fibrotic diseases.^17^

Considering all existing findings, we resorted to the downstream signaling pathways regulated by LOXL2. We have shown that in ligament cells, LOXL2 affects ligament cell endothelial-like differentiation by upregulating the expression of HIF1A, thus elevating VEGFA and PDGF-BB levels. This suggests that broad-spectrum inhibitors on all these targets should effectively inhibit LOXL2-elevated vascular morphogenesis. We observed that an FDA-approved TKI sorafenib could inhibit the progression of ossification in various animal models by breaking neovascularization and osteogenesis coupling. As a chronic degenerative disease, ossification of the posterior longitudinal ligament requires long-term drug maintenance, and the side effects of sorafenib are also one of our concerns. In our toxicological evaluation experiments involving sorafenib, we observed that sorafenib exhibited no impact on normal spinal bone mass. Furthermore, it did not produce any adverse effects on the structural integrity of liver and kidney. As a widely used tumor suppressor, sorafenib was reported to have no severe toxicities (grade 1 or 2) at doses lower than 100 mg bid from phase I clinical trials.^56,57^

Additionally, we observed differential expression patterns of several genes involved in vascular differentiation between HUVECs and ligament cells. Specifically, we found that LOXL2, PDGFB and VEGFA expression was upregulated during endothelial-like differentiation in ligament cells, but not in HUVECs. We also observed an upregulation of osteogenic differentiation regulators, RUNX2 and SP7, during endothelial-like differentiation of ligament cells. Our findings may partially explain the emergence of a new capillary type, H-type blood vessels, characterized by high expression of CD31 and EMCN and elevated levels of RUNX2^+^ and SP7^+^ osteoprogenitors.^58^ This subtype of vessels is closely linked to osteogenesis. Beyond promoting bone development, H-type vessels also play a key role in diseases like Osteoarthritis, Post-Menopausal Osteoporosis and Achilles tendon heterotopic ossification.^59^

In OPLL research, the lack of appropriate animal models has hindered understanding the disease’s pathogenesis. The *ttw* mouse is the most commonly used animal model for OPLL.^3,10,48^ The *ttw* mice exhibit increased angiogenesis^40,60^ and spontaneous ossification of soft tissues due to the loss of *enpp1* inhibition of calcium and phosphorus deposition, which includes the posterior longitudinal ligament.^48^ While some studies have suggested that *enpp1* gene polymorphism may be related to the pathogenesis of OPLL,^61,62^ the phenotype of OPLL patients is not completely identical to that of *ttw* mice resulting from *enpp1* gene deficiency. Some research has proposed using ligament cells derived from OPLL to create scaffolds by placing them on the surface of a demineralized bone matrix, which is then implanted subcutaneously in nude mice to induce heterotopic ossification.^10,63^ However, this animal model using nude mice cannot fully reflect the ossification’s pathological process, as inflammation is a significant factor in physiological and pathological ossification, as evidenced by our sequencing data and previous research.^64^ To overcome these limitations, we used commercially available rhBMP-2 biomaterials subcutaneously implanted into C57BL/6 mice to establish an ossification model (BIO), which we used to evaluate sorafenib’s effectiveness in inhibiting ossification in both BIO and LSO models. Our research provides a reference for the treatment of OPLL and new ideas for other osteogenesis-enhancing diseases such as FOP.

We acknowledge the recent article by Cao et al.^65^ that highlighted methodological shortcomings in previous studies supporting the Endothelial-to-mesenchymal transition (EndoMT) theory. They offered contrasting evidence using inducible *Cdh5-rtTA-tetO-Cre* and *Tek-CreERT2* endothelial lineage tracing models, coupled with single-cell sequencing. They discovered that CDH5^+^BMSCs and TEK^+^BMSCs are indeed two distinct BMSC subpopulations in bone marrow, and it is highly unlikely for these BMSCs to originate from CDH5^+^ and TEK^+^ endothelial cells. Our findings lend further support to their conclusion: ligament cells within the ossification microenvironment of the PLL represent a type of mesenchymal stem cells capable of acquiring an endothelial-like phenotype in vivo and in vitro, and have the potential to form functional blood vessels. This could potentially elucidate the origin of cells expressing both endothelial and mesenchymal markers. Moreover, they did not identify Prrx1 and Lepr-labeled cells expressing endothelial markers in the femurs and tibias of Prrx1-Cre; R26T and Lepr-Cre; R26T mice.^65^ We postulate that this may be attributed to the OPLL microenvironment and heterogeneity of the mesenchymal stem cells. Our study acknowledges several limitations that warrant consideration. Firstly, although our in vivo experiments demonstrated that sorafenib could impede the ossification progression instigated by LOXL2 activation, we were unable to ascertain whether the drug’s impact on neovascularization was attributable to the direct generation of blood vessels by ligament cells or the modulation of pre-existing blood vessels. Secondly, the potential involvement of other factors in regulating neovascularization, such as FGFs, could not be explored within the scope of this article.

## Materials and methods

### Sample preparation

The patients were diagnosed with either ossification of the posterior longitudinal ligament (OPLL) or non-OPLL conditions such as cervical spondylotic myelopathy or radiculopathy without ossification. The diagnosis was based on symptoms, radiological findings from CT, MRI, and X-ray, as well as intraoperative exploration. A standard Anterior Cervical Discectomy and Fusion (ACDF) technique was applied during the surgery. The tissue samples were excised with a Kerrison rongeur, washed twice with saline by a scrub nurse, and transferred to our researcher immediately in wet sterile dressings. After resection, the samples were immersed in sterile saline and transported at 4 °C. The ligaments were rinsed in phosphate buffer saline (PBS) twice and cut into small pieces measuring about 1 mm × 1 mm. These small pieces were evenly spread on the surface of a 6 cm dish and left for 10 min to dry before they were firmly attached to the bottom of the dish. Subsequently, a medium (Mesenchymal Stem Cell Basal Medium, Dakewe, Cat# 6114021) supplemented with 5% serum substitute (EliteGro™-Adv, EliteCell, Cat# EPA-500) and 1% penicillin/streptomycin was carefully placed on the dish to avoid washing away the tissue. The dish was then incubated at 37 °C under 5% CO_2_. The ligament cells were passaged using trypsin and digested for 1 min when they reached 70%–80% confluency. Only cells at passages 2–5 were used for subsequent experiments. To mimic the hypoxic environment in ligaments, an additional 100 μmol/L of cobalt chloride was added to the culture medium of ligament cells during plasmid transfection and tube formation assays. Fasting morning serum samples were obtained after admission using procoagulant tubes, and the tubes were centrifuged at 3 000 r/min for 5 min to separate the serum. The serum samples were aliquoted and stored at −80 °C. Overall, we collected five OPLL patient tissue samples and five non-OPLL patient samples for cell culture and subsequent in vitro experiments; five ossified and three non-ossified patients’ samples were used for staining (Table S7). and 30 OPLL and 14 non-OPLL patients’ serum samples were used for VEGFA Elisa assay(Table S6). The study was approved by the 905th Hospital of PLA Navy Ethics Committee.

### Quantification of ossification and disease severity

Plain X-ray (DRX-Nova, Carestream Health), 1.5 Tesla magnetic resonance (MAGNETOM Aera, Siemens Healthineers), and 64-detector row CT (Sensation 64, Siemens) images were obtained from the 905th Hospital of PLA Navy. To quantify the ossification of patients and mice, we used the ossification index (OS index) and the Occupancy Ratio of the spinal canal. In short, the OS index was used to determine the total segments of disks and vertebral bodies involved in ossification. The Occupancy Ratio of the spinal canal was used to assess the maximal ossification thickness to the diameter of the spinal canal on the sagittal section. This study used a modified Japanese Orthopaedic Association (mJOA) scale to evaluate the disease severity of OPLL patients from 3 dimensions: motor dysfunction, sensory impairment, and bladder dysfunction symptoms. The neck disability index (NDI) was used to evaluate disease severity from the perspective of daily living disability resulting from cervical symptoms, primarily pain.

### Ligament cell transfection

Ligament cells were seeded in 6-well plates, and the siRNAs or plasmids were transfected at 50% confluence. Briefly, 2 μg of plasmid or siRNA (50 nmol/L) was mixed with 200 μL of jetPRIME buffer for 10 s. Then, 4 μL of jetPRIME transfection reagent (Polyplus, Cat# 0000001105) was added according to the manufacturer’s recommendation. After vortex and incubation, the mixture was added to antibiotics-free medium, and then the transfection medium was replaced by a growth medium 5 h later. We checked the transfection efficiency or conducted the following experiments after 36 h. The siRNAs sequences in this study are listed in Table S8.

Empty control plasmids (OE-Ctrl), wild-type LOXL2 overexpression plasmids (OE-LOXL2 WT), and point mutation LOXL2 plasmids (OE-LOXL2 Y689F) were procured from OriGENE Technologies, Inc.

### Tube formation assay with ligament cells

After thawing at 4 °C overnight, we plated Matrigel (Corning, Cat# 356231) in 96-well culture plates and incubated at 37 °C to polymerize for at least 30 min. Transfected siRNA or plasmid ligament cells were digested by trypsin and resuspended in endothelial cell medium (ECM, 1001, ScienCell) supplemented with 5% FBS, 1% endothelial cell growth factors, and 1% penicillin/streptomycin. We then seeded (1 × 10^4^ cells/well) on polymerized Matrigel. Sorafenib Tosylate (Cat# HY-10201A), Imatinib (Cat# HY-50946), recombinant human PDGF-BB (Cat# HY-P7055), VEGFA (Cat# HY-P7110A), VEGFA monoclonal antibody Bevacizumab (Cat# HY-P9906), and isotype control antibody (Cat# HY-P99001) were obtained from MedChemExpress (MCE). We observed and imaged the tube-like structures by microscopy after incubation at 37 °C for 4–6 h. The total length of tube-like structures was calculated with the help of ImageJ software (ImageJ; National Institutes of Health).

### Trilineage differentiation of ligament cells

We seeded ligament cells at 5 × 10^5^ cells per well for osteogenic differentiation in the fibronectin-coated plate. The osteogenic differentiation medium (Cyagen Biosciences, Cat# HUXXC-90021) was changed every 2 days for differentiation. (2-Chloropyridin-4-yl)methanamine hydrochloride (Cat# HY-101771A) was acquired from MedChemExpress as a selective inhibitor of LOXL2 enzyme activity. ALP and Alizarin Red staining were used to testify to differentiation. The Alizarin Red was eluted by 10% cetylpyridinium chloride (*wt/vol*, Sigma-Aldrich, Cat# C9002). The absorbance of the extracted dye was measured at 570 nm. For adipogenesis differentiation, 8 × 10^5^ cells were cultured in an adipogenesis differentiation medium (Cyagen Biosciences, Cat# HUXXC-90031) according to the manufacturer’s protocol for 14 days. Oil red staining was used to testify to differentiation. For chondrogenic differentiation, ligament cells were cultured in a chondrogenic differentiation medium (Sciencell, Cat# SC-7551) supplemented with 10 ng/mL recombinant human TGF-β3 (PeproTech, Cat# 100–36E) for 10 days, and the medium was changed every 2 days. Alcican blue staining was used to testify to differentiation.

### Histochemical and Immunofluorescence analysis

To make paraffin-embedded tissue sections, we take the steps mentioned below. Firstly, fix the dissected tissue in 4% paraformaldehyde for 48 h. After rinsing in PBS, dissected specimens were decalcified in 0.5 mol/L EDTA pH 8.0 regent. Then the samples were dehydrated with ethanol from a concentration of 50%–100%. After soaking in xylene, the samples were transparent. Finally, paraffin was used to embed the tissue. The thickness of the sections was 6μm and prepared with a microtome (Leica RM2235) and mounted on slides. After baking at 62 °C for 1 h, slides were de-paraffined in xylene for 10 min twice. Then, a gradient of ethanol to water was used to rehydrate histological slides. Safranin O/Fast Green (SOFG)and Hematoxylin & Eosin (H&E) staining were conducted for histomorphometric analysis. Masson’s trichrome staining (Servicebio, Cat# G1006) and Sirius red staining (Solarbio, Cat# G1472) were performed according to the manufacturer’s instructions. The citrate antigen retrieval solution was heated to 95–97 °C for 10 min to perform antigen retrieval, and endogenous peroxidase activity was quenched by incubating samples with 3% hydrogen peroxide. Slides were permeabilized with 0.1% Triton X-100. The next steps followed the instructions of an immunohistochemistry Application Solutions Kit (CST, Cat#13079). A standard protocol was followed for immunofluorescence staining. Slides are de-paraffined and rehydrated following the same protocol as mentioned before. Citrate-EDTA antigen retrieval buffer was used 10–12 min after the temperature reached 95–100 °C. After being treated with 0.1% Triton X-100 and blocking, the primary antibody was incubated at 4 °C overnight. Primary antibodies were, anti-COLII (Abcam, Cat# ab34712, 1:100 dilution), anti-SP7 (Abcam, Cat# ab209484, 1:1 000 dilution), anti-BGLAP (SANTA CRUZ, Cat# sc-365797, 1:100 dilution), anti-MMP1(Abcam, Cat# ab52631, 1:100 dilution), anti-COLX (eBioscience, Cat# 14-9771-82, 1:100 dilution), anti-RUNX2 (SANTA CRUZ, Cat# sc-390351, 1:100 dilution), anti-CD31(Servicebio, Cat# GB11063-2-100, 1:200 dilution), specific anti-human CD31 antibody (Abcam, Cat# ab76533, 1:200 dilution), mouse anti-EMCN (Santa Cruz, Cat# sc-65495, 1:50 dilution), human anti-EMCN (Abcam, Cat# ab106100, 1 μg/mL), anti-LOXL2 (Abcam, Cat# ab96233, 1:500 dilution), anti-HIF-1 alpha (Abcam Cat# ab179483,1:500 dilution), anti-PRG4 (Abcam, Cat# ab28484, 1:200 dilution), anti-ACTA2(Abclonal,Cat# A17910, 1:100 dilution). We used Vector® TrueVIEW® Autofluorescence Quenching Kit (Vector Labs, Cat#: SP-8400-15) to minimize non-specific signals. After rinsing in PBS three times, the sections were incubated with secondary antibody for 1 h at room temperature. Slides were sealed with nail polish, and images were taken on the Olympus BX53 microscope or Leica SP8 confocal laser scanning microscope. Quantification was performed using ImageJ software (ImageJ; National Institutes of Health).

### Western blotting

The cell lysates were prepared with RIPA buffer supplemented with protease/phosphatase inhibitor cocktail (MedChemExpress, Cat# HY-K0010) and 100 mol/L PMSF on ice. The relative protein concentration was measured using the BCA protein assay kit (Thermo-PIERCE, Cat# 23225). The protein lysates were separated using SDS-PAGE on 10% polyacrylamide gels, and molecular weight standards were determined with a prestained protein marker. The separated proteins were electro-transferred onto nitrocellulose filter (NC) membranes at ice-cold temperatures, followed by blocking in 5% bovine serum albumin for 1 h at room temperature. The appropriate primary antibodies, including anti-LOXL2 (Abcam Cat# ab96233, 1:1 000 dilution), anti-HIF-1 alpha (Abcam Cat# ab179483, 1:500 dilution), and anti-GAPDH (Abcam Cat# ab9485, 1:10 000 dilution), were rotated with the membrane at 4 °C overnight. Fluorescent secondary antibodies (LI-COR Biosciences, Cat# 926-32211, 1:10 000 dilution) were added for 2 h. Images were obtained using the LI-COR Odyssey CLx system, and densitometric analysis of protein bands was conducted using ImageJ.

### Quantitative RT-PCR

RNA was extracted with Trizol (Takara) to measure the relative mRNA levels. The absorbance ratio and concentration were determined by a NanoDrop 2000 (Thermo Fisher), followed by cDNA synthesis using RT reverse transcriptase kit (Hifair® II 1st Strand cDNA Synthesis SuperMix for qPCR (gDNA digester plus), Yeasen, Cat# 11123ES60) according to protocols. A QuantStudio 3 PCR system (Applied Biosystems/Thermo Fisher Scientific) was used to perform real-time quantitative analysis. The PCR program was 95 °C for 5 min, 40 cycles of following steps: 95 °C for 30 s, 60 °C for 30 s, 72 °C for 30 s, and a total of 20 μL of reaction volume, including 10 μL of 2 × SYBR Master Mix(Hieff® qPCR SYBR Green Master Mix(Low Rox Plus), Yeasen, Cat# 11202ES08), 0.4 μL of each primer and 2 μL of diluted cDNA were used. For normalization, *GAPDH* (housekeeping gene) was used. Primers for amplification were listed in Table S9.

### Transwell cell migration assays

To evaluate the HUVEC (EA. hy926 cell line, Cat# GNHu39, Type Culture Collection of the Chinese Academy of Sciences) migration, we used medium supernatant of transfected siRNA or plasmid ligament cells to generate a conditioned medium. After 30 h of transfection, 4 mL medium supernatant was collected. The supernatant medium was centrifuged using a 3 kDa Ultra-4 Centrifugal Filter Unit (Millipore Amicon™, Cat# UFC800324) at 4 °C and 3 500 r/min for about 30 min to obtain 500 μL of concentrated medium supernatant and added to ECM medium at 1:8 dilution serving as lower chamber medium. HUVECs (2 × 10^4^ cells/well) were seeded into the upper chamber (Falcon Cell Culture Inserts Cat# 353097, Corning) and inserted into the transwell. After 18 h, the upper chamber was fixed with 4% paraformaldehyde (wt/vol) and stained with crystal violet. We wiped off the non-migrated cells with a cotton swab, and the migrated cells were counted with the help of ImageJ (ImageJ; National Institutes of Health).

### Flow cytometry analysis

Primary culture cells from non-OPLL or OPLL were digested with trypsin and washed in 2% FBS. We incubated cells for 30 min at 4 °C with primary antibody obtained from BioLegend at 1:200 dilution, PE anti-human CD45(Cat# 368509), PE anti-human CD73 (Cat# 344003), PE anti-human CD90 (Cat# 328109), FITC anti-human CD105 (Cat# 323203), ABflo® 594 anti-PDGFRA (Abclonal, Cat# A24332), ABflo® 488 anti-CD31/PECAM1 (Abclonal, Cat# A22508), ABflo® 647 anti-PROCR (Abclonal, Cat# A24227). After washing twice with 2% FBS, Fluorescence-activated cell sorting (FACS) was performed via flow cytometry (LSRFortessa™, BD Biosciences) and FACSDiva software (BD Biosciences). Flow cytometry data were analyzed using FlowJo v.10 software (FlowJo).

### Lentiviral construction and transfection

For LOXL2 overexpression, the LOXL2 coding sequence (CDS) was synthesized and cloned into HBLV-EF1-ZsGreen-PURO vectors. All lentiviral expression vectors were synthesized, packaged, and verified by HanBio Biotechnology (HanBio, Shanghai, China). Ligament cells were infected with different lentiviruses at a multiplicity of infection (MOI) of 30 in the presence of 5 µg/mL polybrene for 16–18 h.

### Serum and medium supernatant VEGFA, PDGF-BB analysis

We used a VEGFA (R&D Systems, Cat# DVE00) and PDGF-BB Elisa kit (R&D Systems, Cat# DBB00) to determine the serum and medium supernatant VEGFA or PDGF-BB concentration, following the manufacturer’s instructions.

### Micro-CT analysis

Cervical spines or ossified samples were scanned using x-ray microtomography (SKYSCAN 1272, Bruker Micro-CT, Belgium). The samples were scanned at a set of parameters (60.0 kV, 166.0 μA, and a resolution of 9-μm pixels). Two- and Three-dimensional images were reconstructed by Skyscan NRecon software (Bruker) and CTVox (Bruker Micro-CT), respectively. Bone parameters of the mice heterotopic ossification were calculated using CTan software (Bruker Micro-CT).

### scRNA-seq using 10x genomics

Obtaining ossified and non-ossified ligament tissue followed the steps described above. After thoroughly flushing the blood with saline, the collected samples were stored in MACS Tissue Storage Solution (Miltenyi, Cat# 130-100-008) at 4 °C. Subsequently, the harvested tissues were digested using RPMI 1640 medium (Gibco, Cat#11875093) supplemented with 0.3% type 1 collagenase (Gibco, Cat# 17100017) and 0.4% dispase II (Sigma-Aldrich, Cat# D4693) at 37 °C for 60 min under constant agitation. The digested tissues were then filtered through a series of strainers with pore sizes of 100 μm, 70 μm, and 40 μm. Following the tissue dissociation, the resulting cell suspension underwent treatment with red blood cell lysis buffer to eliminate red blood cells. Subsequently, the cell suspension was subjected to centrifugation, and the cell count was determined. The cells were resuspended at a concentration of 1 000 cells/μL. Due to current technological limitations, achieving a complete digestion of bone tissue to isolate its constituent cells was not feasible. Consequently, single-cell sequencing was conducted to examine cells from the PLL that displayed impending ossification and those from the non-ossified portion. Single-cell libraries were prepared using the Chromium Controller from 10x Genomics (Pleasanton, CA), following the protocol outlined in the Chromium Next GEM Single Cell 3’ Reagent Kits (v3.1, 10x Genomics). Libraries were prepared using the Chromium Controller from 10x Genomics. The final library pool was subjected to sequencing on the Illumina Novaseq6000 instrument, generating 150 base-pair paired-end reads. Alignment to the GRCh38 reference genome, quantification of gene expression, and aggregation of sample count matrices were performed using the cellranger pipeline version 6.1.1.

### Bioinformatics analysis of single-cell sequencing data

Data set integration, dimensionality reduction, and clustering were conducted with Seurat version 4.3.0. Cells meeting the criteria of nFeature_RNA > 200, nFeature_RNA < 5 000, and <15% mitochondrial genes were subjected to further processing. Sctransform package version 0.3.5 and harmony package version 0.1.1 were employed to address batch effects. UMAP (Uniform Manifold Approximation and Projection) dimensionality reduction was applied using the top 50 components. For initial clustering, 40 dimensions of reduction were used as input, and the Louvain algorithm with a resolution of 0.2 was employed through the Findcluster function. Among the clusters, two displayed high hemoglobin gene expression, indicating potential red blood cell contamination. Consequently, these cells were removed. To perform sub-clustering of ligament cells, pericytes, and endothelial cells, dimensionality reduction and clustering were carried out using the Monocle3 package version 1.3.1. Eight subclusters were identified using a resolution of 3e-5. Single-cell trajectory analysis was performed on the cell clusters using the Monocle3 package. UMAP was used for dimensionality reduction, and the learn_graph function in Monocle3 was employed for pseudotime trajectory construction, with the C6_progenitor designated as the root node. The identification of the root node was facilitated by the CytoTrace package version 0.3.3 based on cytoTrace scores and velocyto (version 0.2.5) based on RNA velocity. Furthermore, the find_gene_module function was utilized to group co-regulated genes into modules using Louvain community analysis. Module scores were calculated by aggregating the expression of all genes within each module across all clusters and were visualized using heatmaps. Functional enrichment analysis of each module was performed using the ReactomePA package version 1.42.0.

### Transcriptome sequencing

The total RNA was extracted with TRIzol reagent (Invitrogen). The RNA amount and purity quantification, RNA integrity number (RIN) assessment, library preparations, and paired-end sequencing were performed by Origin-gene Bio-Pharm on a novaseq6000 platform (Illumina).

### Protein-protein interaction (PPI) network construction

All the DEGs identified in the tissue transcriptomic sequencing were uploaded to an online database - STRING, and analyzed with default parameters. The tsv format files were downloaded and imported into Cytoscape software (Version: 3.8.2). Cytoscape plugin cytoHubba for network centrality analysis was used to identify a network’s critical nodes (genes). Our study used 5 specific centrality measures to identify critical genes (hub genes) by calculated scores. The methods were listed: Maximal Clique Centrality (MCC), Density of Maximum Neighborhood Component (DMNC), Edge Percolated Component (EPC), Maximum Neighborhood Component (MNC), and ClusteringCoefficient.

### Bioinformatics analysis

Firstly, adapter contamination, low-quality bases, and undetermined bases were removed by trim_galore version 0.6.6, and sequence quality was verified using FastQC v0.11.9. We used HISAT2 version 2.2.1 and samtools version 1.7 to map filtered reads to the human genome GRCh38.p13 in Ensemble104. After the final transcriptome was generated, featureCounts version 2.0.1 was used to obtain strand-specific read counts. The Differentially expressed genes (DEGs) were selected with |log_2_ (fold change)| > 1 and with statistical significance (*P* < 0.05) by R package edgeR. Then clusterProfiler was used for Gene Ontology (GO) functional annotation analysis and Kyoto Encyclopedia of Genes and Genomes (KEGG) pathway enrichment analysis, and Gene set enrichment analysis (GSEA).

### Animals

For the BMP-induced in vivo ossification (BIO) model, male C57BL/6J mice (6 weeks old) were obtained from Shanghai Leigen Biotechnology Co., Ltd. After being anaesthetized with an intraperitoneal injection of Avertin, a longitudinal incision of ~1 cm was made on the back of each mouse. Then, an rhBMP-2 biomaterial (Hangzhou Jiuyuan Gene Engineering Co., Ltd., China) containing gelatin, soy lecithin, and hydroxyapatite was implanted under the skin and secured to the back muscles with a 4–0 absorbable collagen thread. The surgical incisions were closed with 5–0 nylon sutures. The mice were housed in a laminar airflow cabinet with specific pathogen-free conditions, a room temperature of 22 °C, and a 12-h light/dark cycle. On the second postoperative day, sorafenib was resuspended in 0.5% sodium carboxymethylcellulose at a concentration of 50 mg/kg body weight. The intervention mice were administered sorafenib by gavage once a day for 14 days, while the vehicle group was given the same volume of sodium carboxymethylcellulose by intragastric administration. The mice were sacrificed by excessive anesthesia, and the heterotopic ossification on the back was excised for subsequent analysis.

For Ligament Spontaneous Ossification (LSO), *enpp1* KO mice (strain NO.T013898) were obtained from GemPharmatech (Nanjing, China). Four-week-old *enpp1* KO mice were used in this study, and sorafenib was administered orally to the mice daily. The cervical spines were collected at 18 weeks. The raising, administration, and sample collection strategies were the same as those described in the BIO model. The genotypes of transgenic mice were determined by PCR (Absin, Cat# ABS60036) using genomic DNA isolated from mice tails. The primer pairs are listed in Table S10.

### In vivo Matrigel plug assay

Nude mice aged 6–8 weeks were anaesthetized with Avertin. The back skin of the nude mice was gently lifted, and 0.2 mL of cold Matrigel, with or without 2 × 10^6^ ligament cells, was injected into the subcutaneous right and left dorsolateral sides to create hillock-like and pushable bulges. After 7 days, the mice were anaesthetized and perfused sequentially with heparinized saline, 4% PFA, and ink (Kuretake, Cat# CE100-6) through the left ventricle. Subsequently, the previously injected Matrigel was removed once the nude mice had utterly darkened. The ink particle deposits in lumen-like structures on sections indicated the formation of functional blood vessels. The dyes Ulex Europaeus Agglutinin I (UEA I, Cat#DL-1067) and Griffonia Simplicifolia Lectin I Isolectin B4 (GS-B4, Cat#DL-1208) used for fluorescence staining were purchased from Vector Laboratories.

### Evaluation of the sensory and motor function of mice

To evaluate the effects of sorafenib on mice’s sensory and motor function, mechanical and thermal nociception hyperalgesia tests were adopted. Research blinded to the study groups used electronic von Fray filaments (BIO-EVF4, Bioseb, France) to evaluate mechanical sensitivity and plantar test apparatus (Ugo-Basile 37370, Comerio, Italy) to perform thermal hyperalgesia measurement. Following our previous protocol, mice were placed in cages or on the glass plate for 30 min before the test started. Three experimental data were recorded as manufacturer’s instructions when the hind paws began to buckle for the mechanical nociception hyperalgesia test. For the thermal hyperalgesia test, we recorded the time from irradiation to mouse hind paw withdrawal, at least a 30-min interval between trials. Three experimental data of each mouse were recorded as response times.

### Statistical analysis

Data were presented as mean value ± SD (standard deviation) except for some baseline characteristics, which were reported as counts and percentages for categorical variables or median and interquartile range (IQR) when normality assumptions were not met. Student’s *t*-test or One-way ANOVA with Tukey’s multiple comparisons was performed as appropriate, while the Mann-Whitney U test or Kruskal-Wallis test was used for non-normally distributed data. Pearson correlation was used to assess the correlation between two variables. The diagnostic performance of serum VEGFA for OPLL was determined using a ROC curve and the area under the ROC curve (AUC). Baseline characteristics were presented as mean ± SD for continuous variables, counts, and percentages for categorical variables. Student’s *t*-test and chi-squared tests or Fisher’s Exact Test were used to compare continuous and categorical characteristics, respectively, between non-OPLL and OPLL. Duration of symptoms was tested using covariance analysis with a univariate general linear model, adjusting for age. All statistical analyses were performed using SPSS 23.0 (IBM, Chicago) and GraphPad Prism 8.0 (GraphPad, San Diego). Statistical significance was considered at *P* < 0.05 (*P < 0.05, **P < 0.01, ***P < 0.001), while ns indicated non-significance. At least three independent experimental groups were analyzed.

### Supplementary information

supplementary materials

## Data Availability

All data of this study are present in the paper, and the sequencing data will be available at NCBI BioProject ID: PRJNA902554 and PRJNA1067846.

## Materials availability

All materials of this study are present in the paper.
