# Supplementary material for: Sorafenib inhibits ossification of the posterior longitudinal ligament by blocking LOXL2-mediated vascularization
Source: Bone Res. 2024 Apr 10;12:24. doi: 10.1038/s41413-024-00327-7 (PMC11004159; doi:10.1038/s41413-024-00327-7)
Supplement: Supplementary file 1 — supplementary materials [file 41413_2024_327_MOESM1_ESM.docx]

**supplementary materials**

**
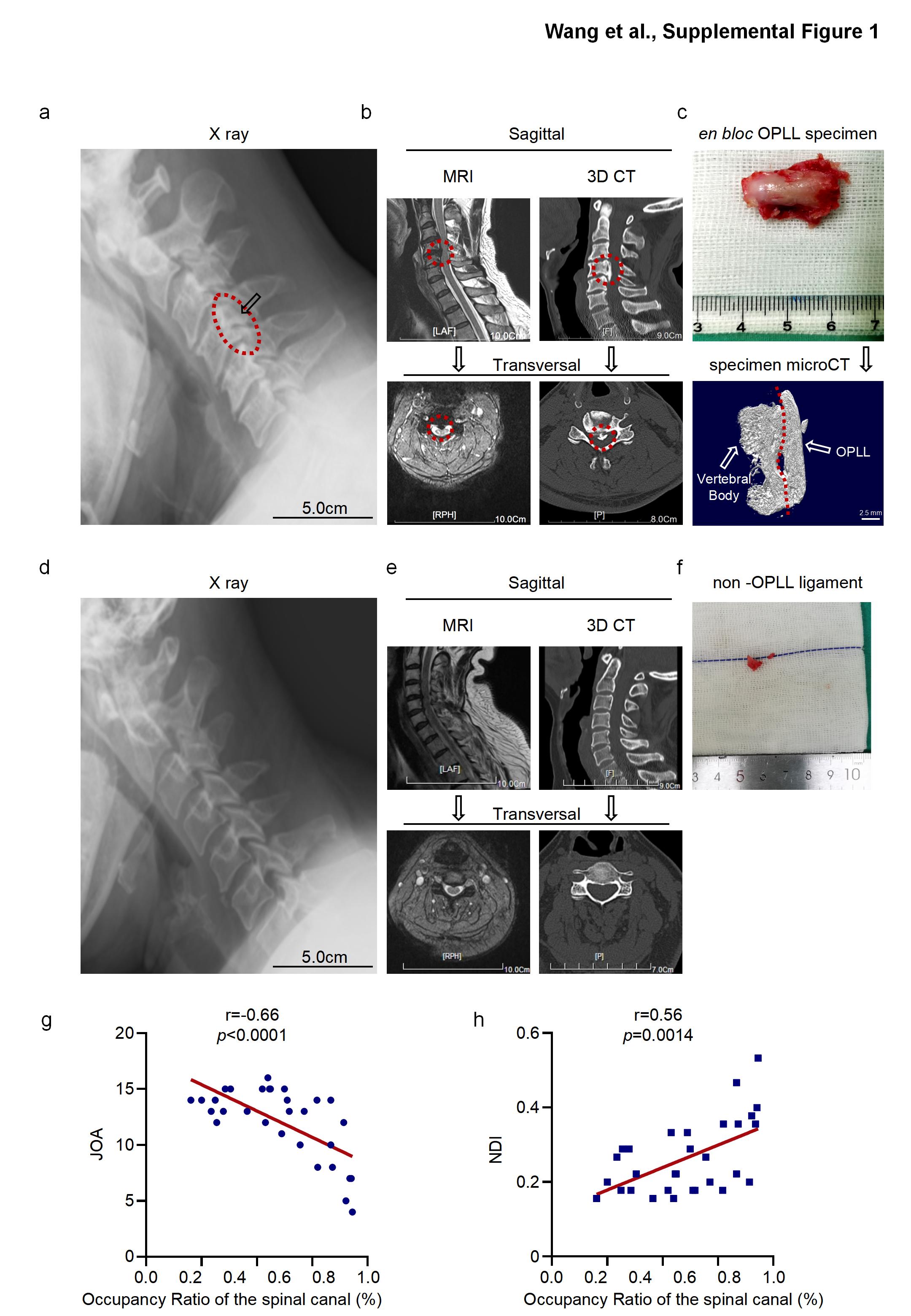
**

**Supplemental Figure 1. Characterization of Non-OPLL and OPLL.**

**(a)** Representative lateral radiograph of the cervical spine in an Ossification of the Posterior Longitudinal Ligament (OPLL) patient. The red dashed circle and black arrow highlight ossifications protruding into the spinal canal. Scale bars = 5 cm. **(b)** Sagittal and transverse images of Magnetic Resonance Imaging (MRI, left) and three-dimensional Computed Tomography (3D-CT, right) in a typical OPLL patient. The red dashed circles indicate ossifications protruding into the spinal canal, leading to spinal cord compression. MRI: Scale bars = 5 cm; 3D-CT: Scale bars = 1 cm. **(c)** Representative image of an en bloc OPLL specimen (upper) along with a microCT image (lower). En bloc OPLL specimen: Scale bars = 1 cm; microCT: Scale bars = 2.5 cm. **(d)** Representative lateral radiograph of a non-OPLL patient (with cervical spondylotic myelopathy or radiculopathy without ossification). Scale bars = 5 cm. **(e)** Sagittal and transverse images of Magnetic Resonance Imaging (MRI, left) and three-dimensional Computed Tomography (3D-CT, right) in a typical non-OPLL patient. MRI: Scale bars = 5 cm; 3D-CT: Scale bars = 1 cm. **(f)** Representative image of an *en bloc* non-OPLL specimen. Scale bars = 1 cm. (**g and h**) Correlations between the occupancy ratio of the spinal canal and the Japanese Orthopaedic Association (JOA) score (r = -0.66) and Neck Disability Index (NDI) score (r = 0.56) were assessed using the Pearson correlation test.**
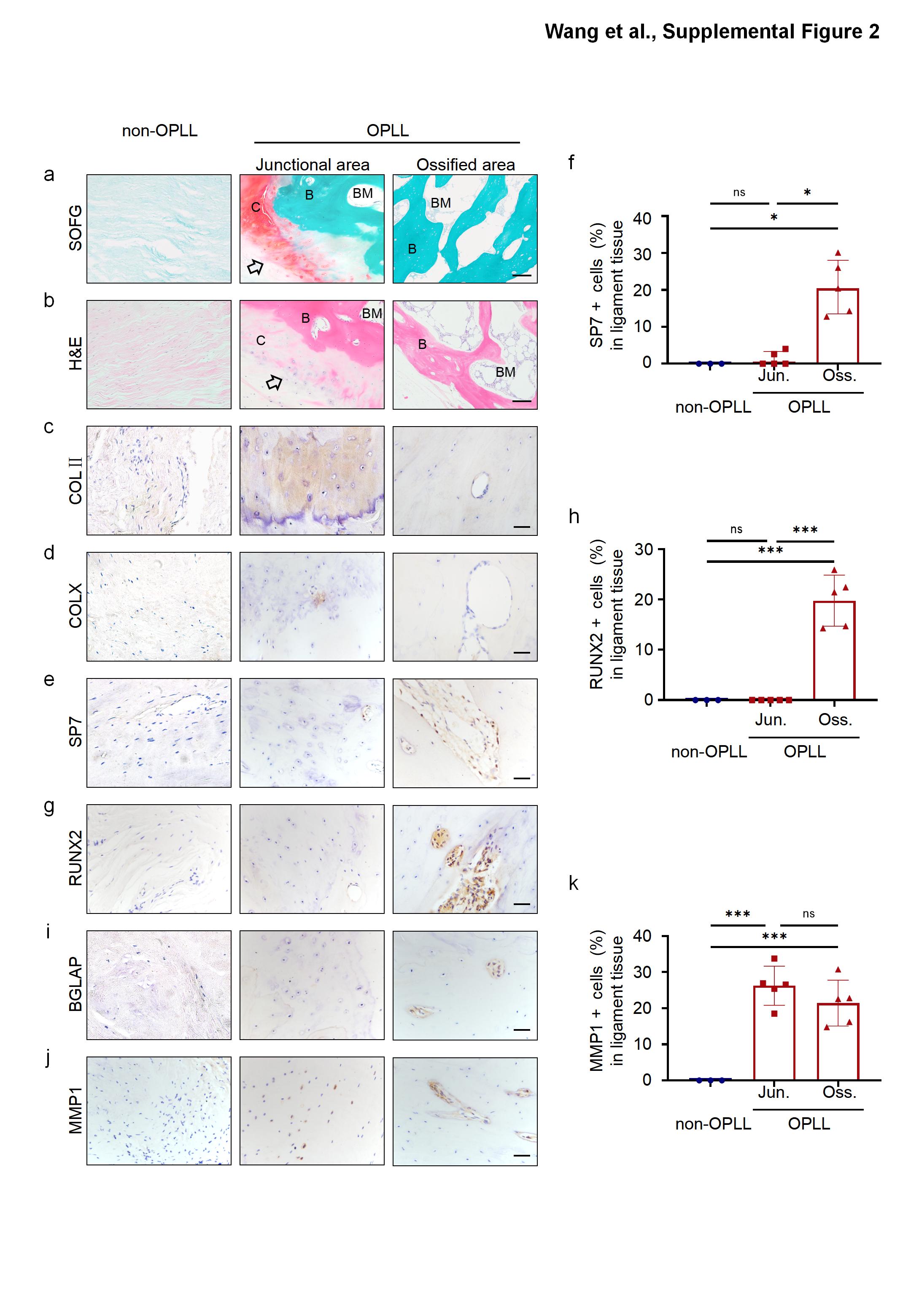
**

**Supplemental Figure 2. OPLL is characterized by endochondral ossification according to the radiological and histological features.**

**(a)** Safranin O and Fast Green (SOFG) staining of the posterior longitudinal ligament without ossification (non-OPLL, left) and ossified posterior longitudinal ligament (OPLL) of junctional area (middle) and ossified area (right). Proteoglycan (red) and bone (green). Scale bar = 200 μm. **(b)** H&E staining of non-OPLL (left) and OPLL. Scale bar = 200 μm. Black arrows indicate the junctional area of non-OPLL and OPLL. C: cartilage, B: bone, BM: bone marrow. **(c and d)** Immunohistochemistry (IHC) staining of COLⅡ and COLX. **(e-h)** SP7^+^ and RUNX2^+^ cells (left) and quantitative analysis of the percentage of SP7^+^ or RUNX2^+^ cells (right). *p < 0.05, ***p < 0.001, ns not significant by ANOVA. **(i)** Representative IHC staining image of BGLAP. **(j and k)** MMP1^+^cells and quantitative analysis of the percentage of MMP1^+^ cells. ***p < 0.001, ns not significant (ANOVA), Scale bar = 40 μm. All data are shown as the mean ± s.d. n = 3 for the non-OPLL group and n = 5 for the OPLL group.

**
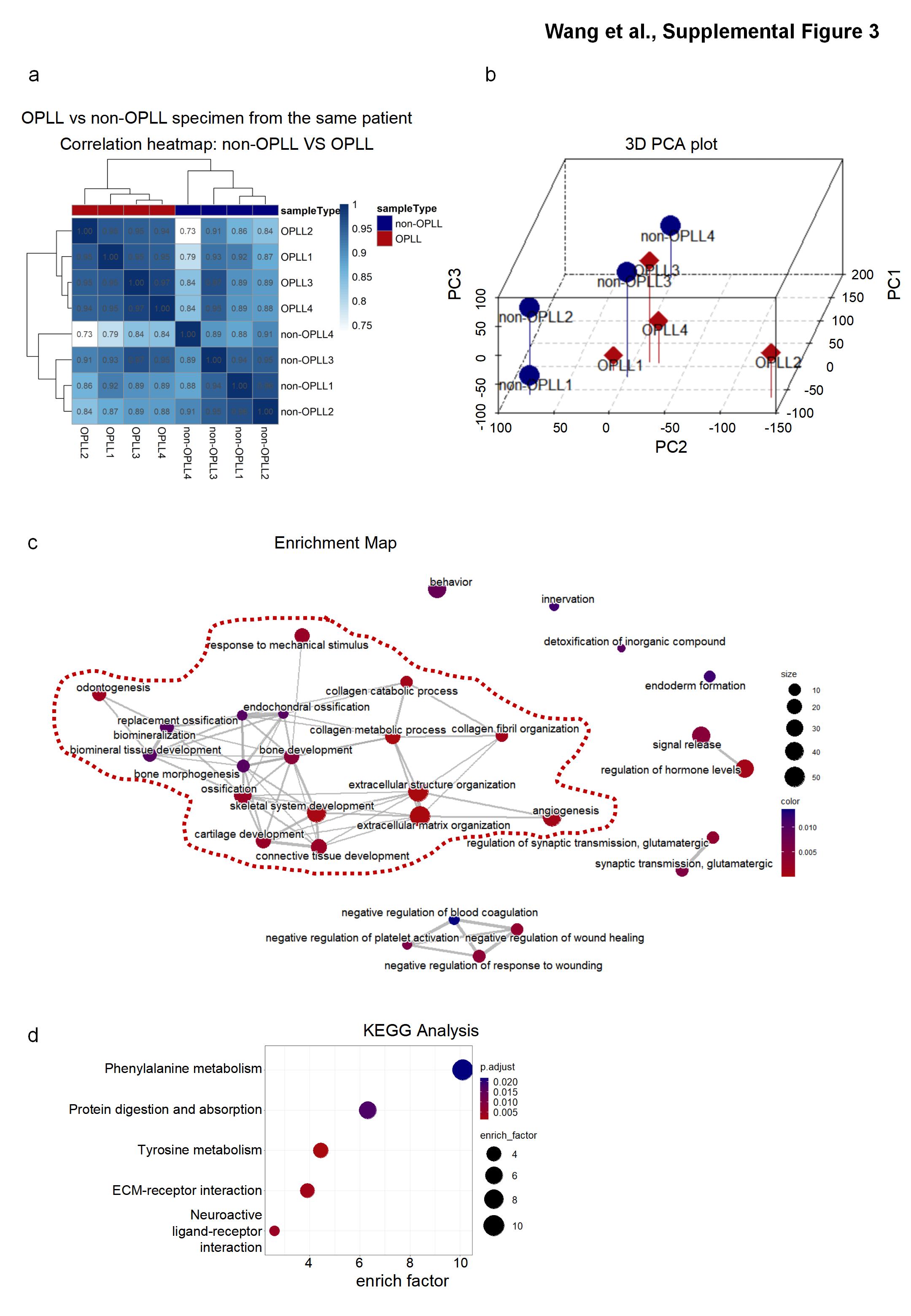
**

**Supplemental Figure 3. High-throughput sequencing analysis of OPLL and non-OPLL specimens.**

**(a)** Correlation Heatmap displaying pairwise relationships between non-OPLL and OPLL. Positive correlations are shown in shades of blue, with darker shades indicating a stronger relationship. **(b)** 3D PCA (Principal Component Analysis) Plot illustrating the distribution of all non-OPLL and OPLL sequencing samples in the reduced-dimensional space; the axes are labeled as PC1, PC2 and PC3. **(c)** Enrichment Map displaying the functional enrichment of differential GO signalling pathways in OPLL. Nodes represent differential GO signaling pathways, while edges connect nodes with significant overlap in their gene content. Node size is proportional to the number of genes in the pathway, and color intensity indicates the adjusted P value (red represents a higher adjusted P value). **(d)** KEGG analysis. Each node represents an individual signaling pathway, while node size represents enrich factor and color-coded boxes distinguish adjusted P value (red represents higher adjusted P value).

**
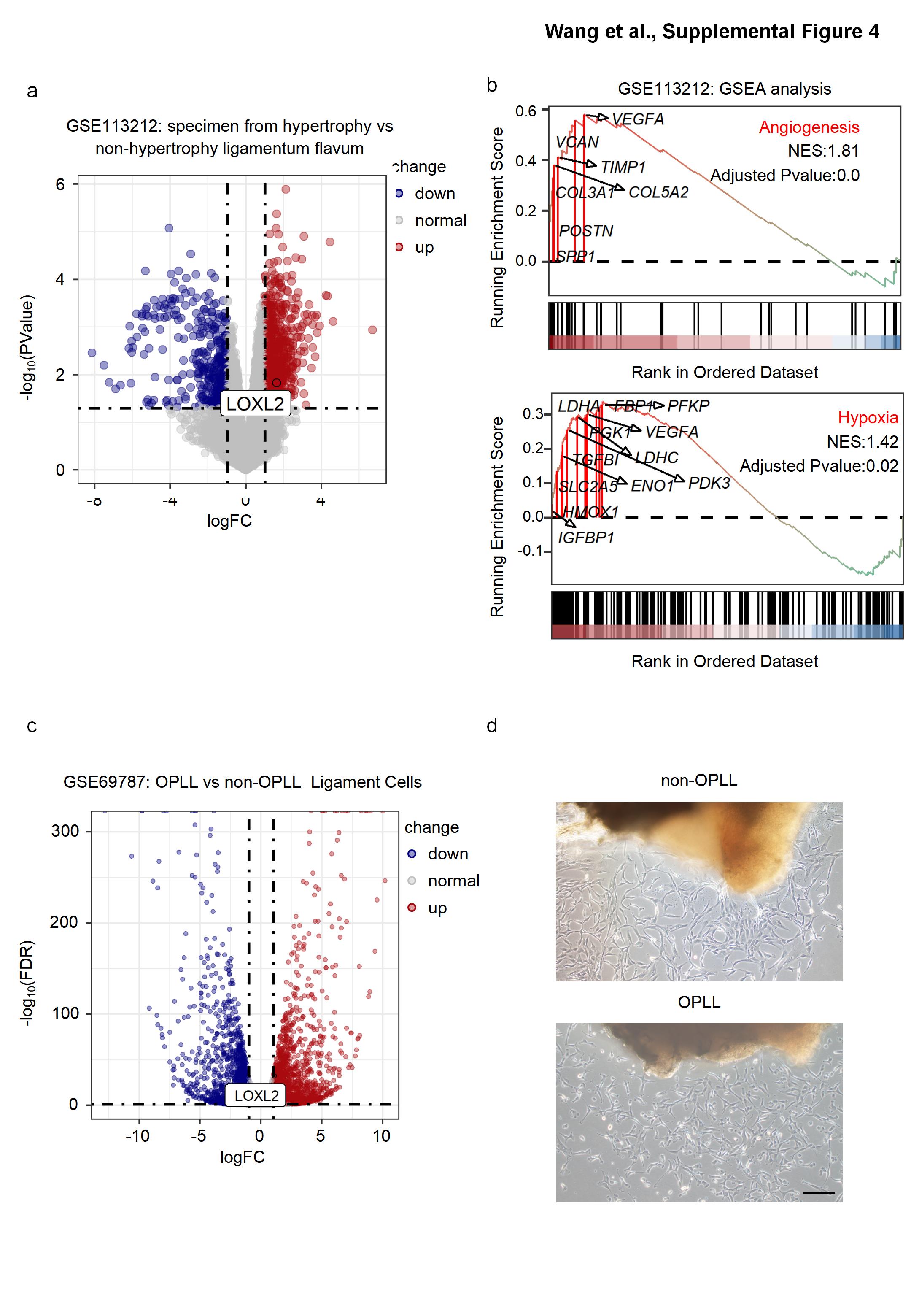
**

**Supplemental Figure 4. Characteristics of hypertrophic ligaments and ligament cells.**

**(a)** Microarray analysis of specimen from hypertrophy vs non-hypertrophy ligament. Volcano Plot depicting the differential gene expression analysis results. Each point represents an individual gene, with the x-axis indicating the log2 fold change (log2FC) and the y-axis displaying the negative log10 of the P value (-log10(P value)). Red and blue dots represent significantly regulated genes (P < 0.05) with a fold-change higher than 2 or lower than 0.5. **(b)** Representative enrichment plots of curated MSigDB Hallmark Gene Set Enrichment Analysis (GSEA). The normalized enrichment score (NES) and adjusted P value are displayed in the plot to assess the statistical significance of the enrichment. Gene names are DEGs in Angiogenesis and Hypoxia pathway. The GSEA plot helps identify functionally related gene sets or pathways significantly enriched in hypertrophic ligaments. **(c)** Volcano Plot depicting the differential gene expression analysis of OPLL and non-OPLL primary culture ligament cells. The x-axis shows log2 fold change (log2FC), and the y-axis displays -log10(FDR). Upregulated genes (red) are on the right, and downregulated genes (blue) are on the left. The dashed lines represent the significance and fold change cutoffs. **(d)**Migration of ligament cells from non-OPLL and OPLL samples, demonstrating spindle-shaped morphology. Scale bar = 200 μm. n = 3.

**
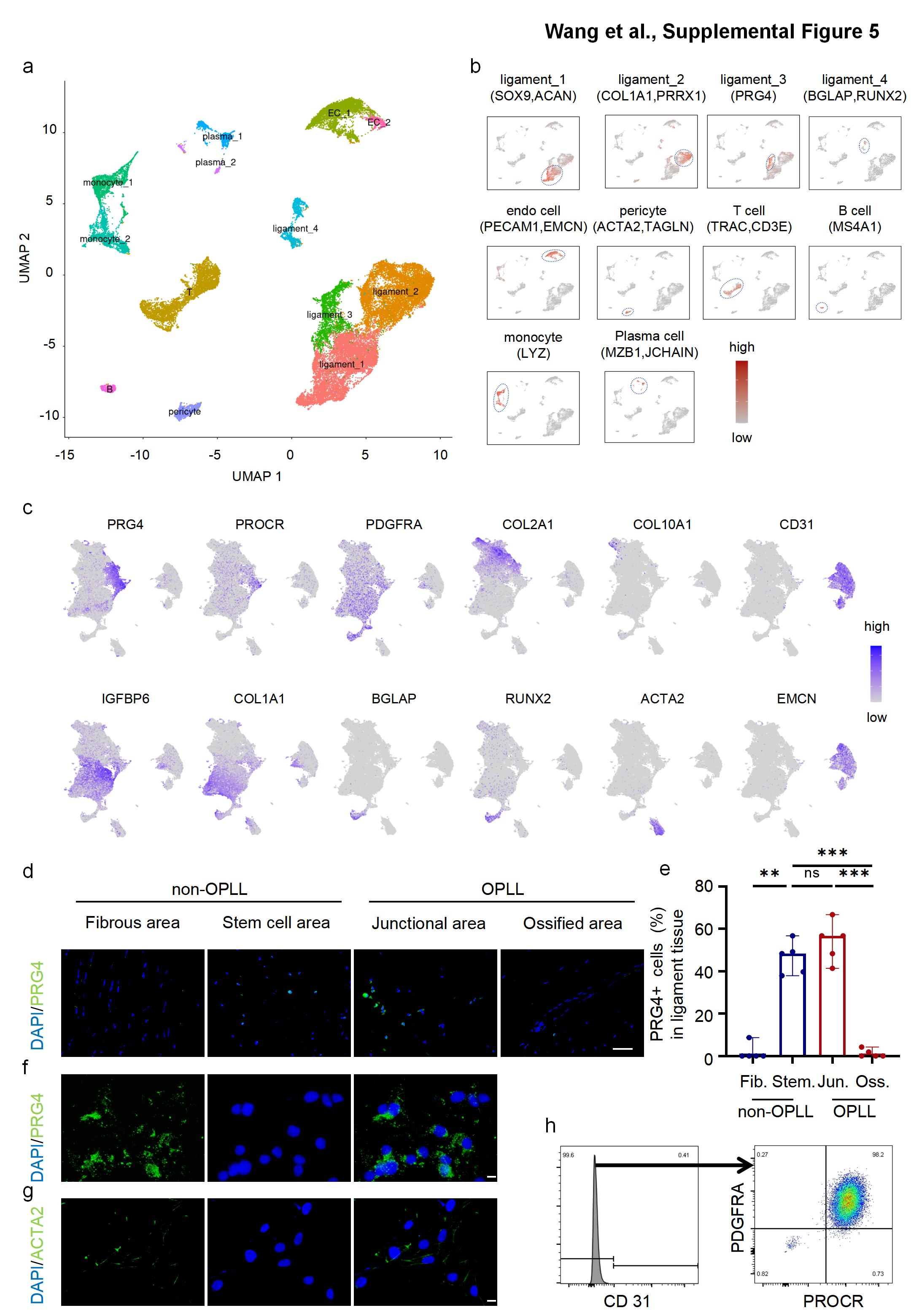
**

**Supplemental Figure 5. Single-cell transcriptomic landscape of human posterior longitudinal ligament cells.**

**(a)** Distribution of 42,698 cells derived from the human posterior longitudinal ligament. Among them, 15,048 cells were obtained from the posterior longitudinal ligament of patients with Ossification of the Posterior Longitudinal Ligament (OPLL), while 27,650 cells were derived from the posterior longitudinal ligament of patients with cervical spondylosis without ossification (non-OPLL). A UMAP plot visualizes 13 distinct cell clusters, including ligament cells (ligament_1~4), endothelial cells (EC_1~2), pericytes (pericyte), monocyte-macrophages (monocyte_1~2), T cells (T), B cells (B), and plasma cells (plasma_1~2). UMAP stands for Uniform Manifold Approximation and Projection. **(b)** UMAP plot displaying the average expression of curated feature genes for the cell clusters defined in **(a)**. The color bar represents the expression level of each gene, with darker colors indicating higher expression. The virtual coil represents cell clusters marked by curated feature genes. **(c)** Dot plots of marker genes for various cell types in ligament cells, endothelial cells and pericytes, with darker colors of the color bar indicating higher gene expression. (**d** **and e**) Representative images for IF staining (left) of PRG4 (green) and quantification (right). Scale bar=50 μm. Values are means ± s.d. n = 3 for the non-OPLL group and n = 5 for the OPLL group, ***P < 0.001 by Student’s t-test. **(f and g)** Representative immunofluorescence staining of PRG4 and ACTA2 as indicated in ligament cells. Scale bar=10 μm.n=5. (**h**) Representative FACS plots of primary cultured ligament cells, showing CD31^-^ and PDGFRA^+^PROCR^+^ phenotypes.

**
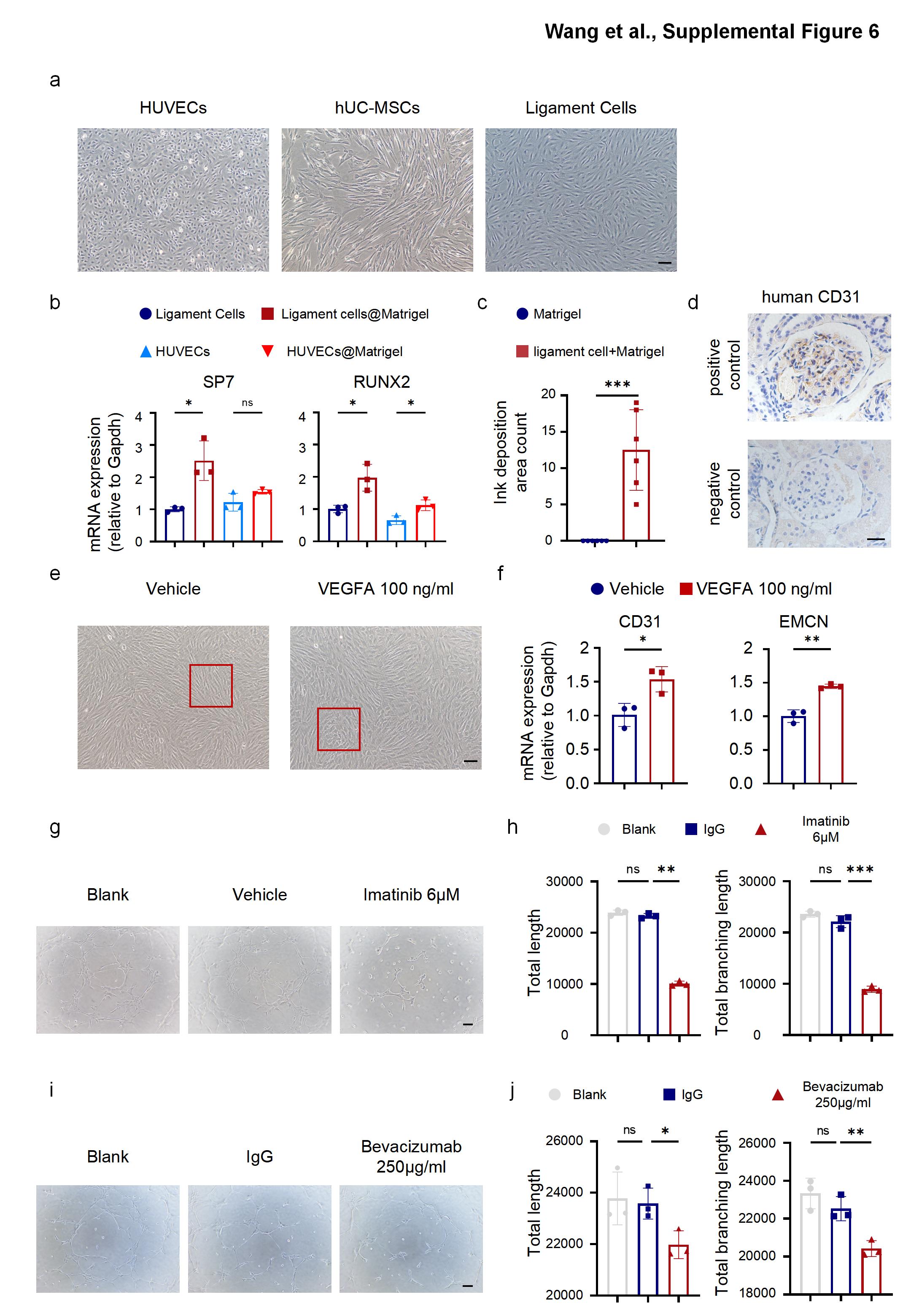
**

**Supplemental Figure 6. Characteristics of ligament cells endothelial-like differentiation.**

**(a)** Representative images of human umbilical vein endothelial cells (HUVECs), human umbilical cord mesenchymal stem cells (hUC-MSCs), and ligament cells, highlighting the distinct morphologies of each cell type in culture. HUVECs exhibit a cobblestone-like appearance, whereas hUC-MSCs and ligament cells display a fibroblast-like spindle shape. Scale bar = 100 μm. n = 3. **(b)** Quantitative PCR (qPCR) results showing gene expression levels of *SP7* and *RUNX2*, normalized to the housekeeping gene *GAPDH*, in ligament cells and HUVECs cultured with or without Matrigel. Values represent means ± s.d., n = 3. *P < 0.05, ns (not significant) by Student's t-test. **(c)** Quantification of ink deposition on sections of Matrigel spheres loaded or unloaded with ligament cells. **(d)** Representative images of negative control (mouse glomerulus) and positive control (human glomerulus) of CD31 antibody. Scale bar = 100 μm. **(e)** Representative images of ligament cells treated with PBS (Vehicle) or 100 ng/ml VEGFA. Red boxes emphasize the morphological changes in ligament cells, transitioning from a spindle shape to a more rounded appearance upon VEGF stimulation. Scale bar = 100 μm. n = 3. **(f)** Quantitative PCR (qPCR) results illustrating gene expression levels of CD31 and EMCN, normalized to housekeeping gene GAPDH, in ligament cells treated with PBS (Vehicle) or 100 ng/ml VEGFA. Values indicate means ± s.d., n = 3. *P < 0.05, **P < 0.01 by Student's t-test. **(g and h)** Representative images display capillary-like structures formed by OPLL-derived ligament cells on Matrigel under distinct conditions (left): unstimulated (Blank), ddH_2_O (Vehicle), and Imatinib (6 μM), including quantification (right). Scale bar = 100 μm. Values are means ± s.d. n = 3. **P < 0.01, ***P < 0.001, ns not significant by ANOVA. **(i and j)** Representative images display capillary-like structures formed by OPLL-derived ligament cells on Matrigel under distinct conditions (left): unstimulated (Blank), IgG isotype control (IgG), and Bevacizumab (250 μg/ml), including quantification (right). Scale bar = 100 μm. Values are means ± s.d. n = 3. *P < 0.05, **P < 0.01, ns not significant by ANOVA. The experiments were performed in three biological replicates.

**
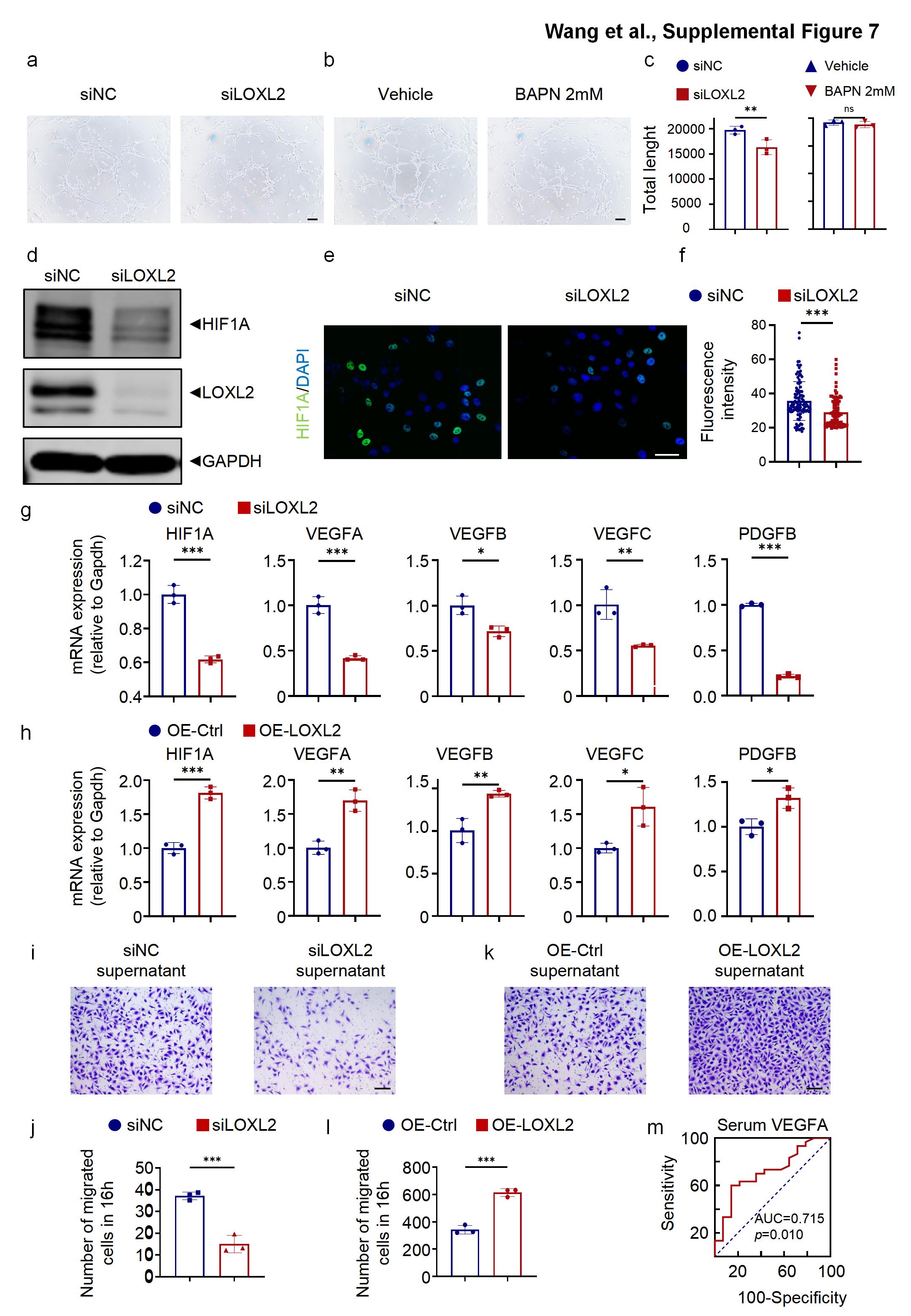
**

**Supplemental Figure 7. LOXL2 modulates ligament cell endothelial-like differentiation through the HIF1A signalling pathway.**

**(a)** Representative images of ligament cells vasculogenesis *in vitro*. The vasculogenesis ability of ligament cells was determined by tube formation assay. Ligament cells were transfected with scramble control siRNAs (siNC) and siLOXL2. Tube formation ability is inhibited when knocking down LOXL2. Scale bars = 100 μm. **(b)** Representative images of capillary-like structures. Ligament cells were treated by PBS (Vehicle) or 2mM BAPN. Scale bars = 100 μm. **(c)** Quantification of tube formation of ligament cells. Histogram shows the relative total length of tubes (pixels). Values are mean ± s.d. n = 3. **p < 0.01, ns not significant by Student’s t-test respectively. **(d)** Representative images of LOXL2 and HIF1A protein of ligament cells by western blotting. Ligament cells were treated with scramble control siRNAs (siNC) and siLOXL2. **(e and f)** Representative immunofluorescence staining of HIF1A and quantification by fluorescence intensity. Ligament cells were transfected with scramble control siRNA (siNC) and siLOXL2. Scale bars = 50 μm. n = 3 and 100 cells were randomly selected. ***p < 0.001 by Student’s t-test. **(g)** Expression levels of *HIF1A*, *VEGFA*, *VEGFB*, *VEGFC,* and *PDGFB* relative to GAPDH of ligament cells transfected with scramble control siRNAs (siNC) and siLOXL2. Values are means ± s.d. n = 3. ***P < 0.001, **P < 0.01, *P < 0.05, ns not significant by Student’s t-test. **(h)** Expression levels of *HIF1A*, *VEGFA*, *VEGFB*, *VEGFC,* and *PDGFB* relative to GAPDH of ligament cells transfected with over-expression (OE) LOXL2 plasmids (OE-LOXL2) and empty vector plasmids (OE-Ctrl). Values are means ± s.d. n = 3. ***P < 0.001, **P < 0.01, *P < 0.05, ns not significant by Student’s t-test. **(i and j)** Transwell assay results illustrating HUVECs migration under ECM medium supplemented supernatant derived from ligament cells transfected with scramble control siRNAs (siNC) and siLOXL2. Representative images of the transwell membrane (left) and quantitative analysis (right). Scale bar = 100 μm. Values are means ± s.d. n = 3. ***P < 0.001 by Student's t-test. **(k and l)** Transwell assay results illustrating HUVECs migration under ECM medium supplemented supernatant derived from ligament cells transfected with LOXL2 plasmids (OE-LOXL2) and empty vector plasmids (OE-Ctrl). Representative images of the transwell membrane (left) and quantitative analysis (right). Scale bar = 100 μm. Values are means ± s.d. n = 3. ***P < 0.001 by Student's t-test. **(m)** ROC curves for diagnosis. ROC shows the performance of serum VEGFA to diagnose OPLL, and the area under the curve (AUC) was 0.715, P = 0.01. n = 14 for non-OPLL group and n = 30 for OPLL group. The experiments were performed in three biological replicates.

**
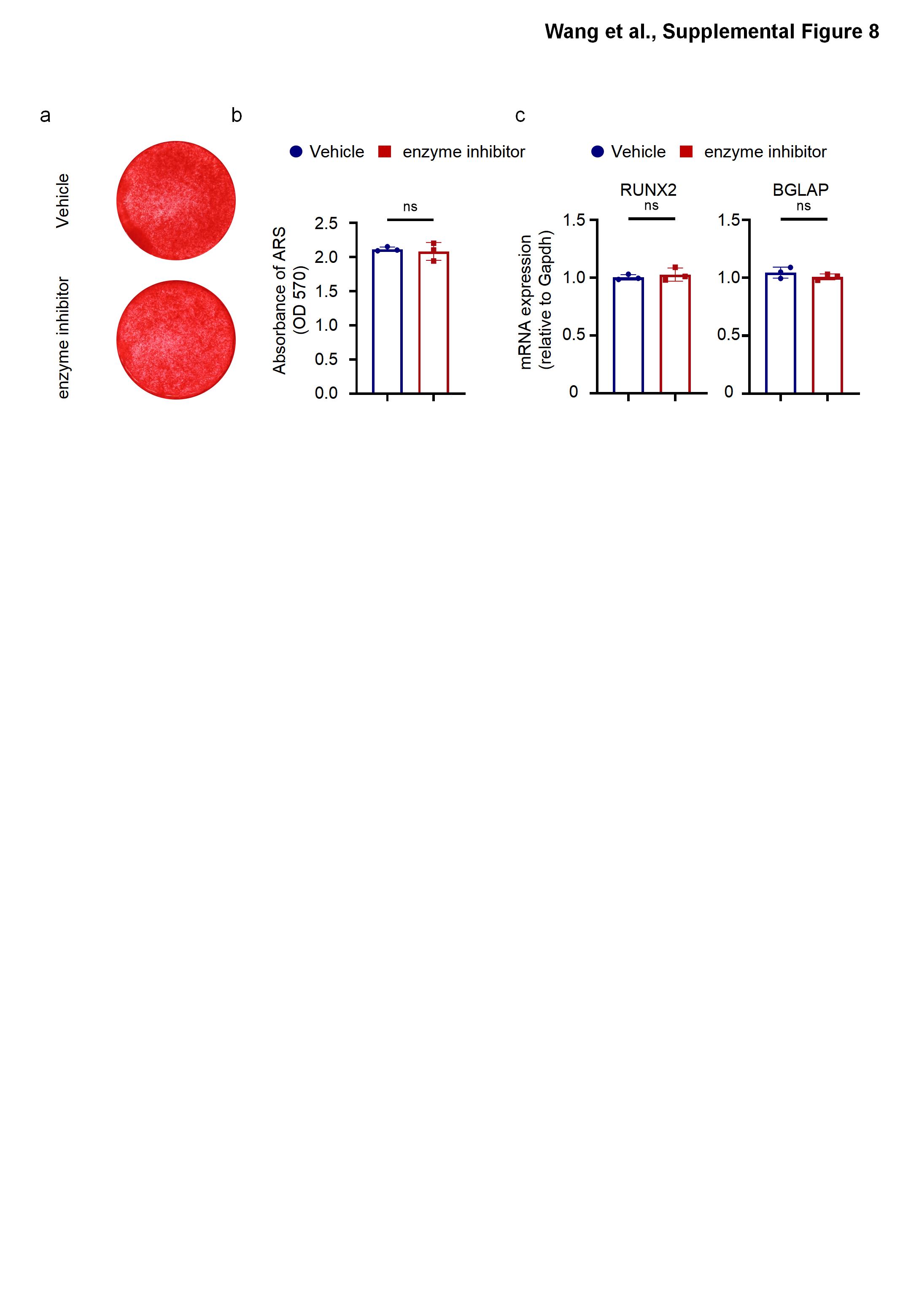
**

**Supplemental Figure 8. Effect of LOXL2 enzyme activity on osteogenic differentiation of ligament cells**

**(a and b)** Representative images of Alizarin Red S staining (left) and quantitative analysis (right) of ligament cells treated with PBS (Vehicle) and 126 nM selective LOXL2 enzyme inhibitor-(2-Chloropyridin-4-yl)methanamine hydrochloride. **(c)** Quantitative PCR (qPCR) results illustrate the gene expression levels of *RUNX2* and *BGLAP* relative to housekeeping gene GAPDH generated from ligament cells stimulated with PBS (Vehicle) and 126nM LOXL2 enzyme inhibitor. Values are means ± s.d. n = 3. ***P < 0.001, **P < 0.01 by Student’s t-test.

**
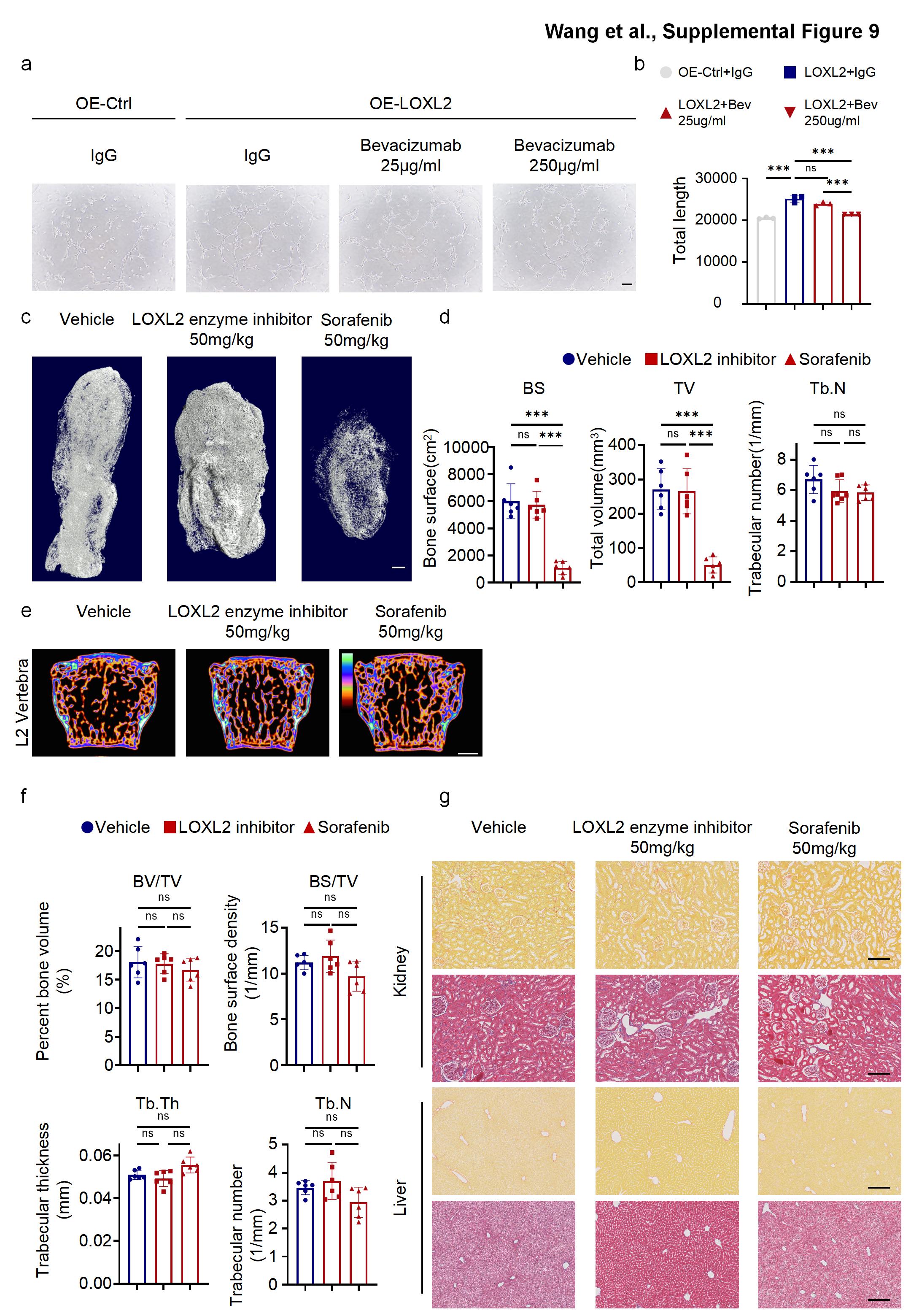
**

**Supplemental Figure 9. Sorafenib attenuates the progression of the BMP-induced In vivo Ossification (BIO) model.**

**(a and b)** Representative pictures of capillary-like structures formed on Matrigel (left) of ligament cells overexpressing empty vector plasmids (OE-Ctrl) and LOXL2 plasmids (OE-LOXL2) with the treatment of Bevacizumab (250μg/ml) or IgG isotype control (IgG); alongside quantification (right). Scale bar = 100 μm. Values are means ± s.d. n = 3. ***P < 0.001, ns not significant by ANOVA. (**c and d**) Representative reconstructed 3D micro-CT coronal images depict the ossification site following a 2-week treatment with 50 mg/kg Sorafenib, an LOXL2 enzyme inhibitor [(2-Chloropyridin-4-yl)methanamine hydrochloride] and a methylcellulose carrier alone (Vehicle). Quantitative analysis of structural parameters related to heterotopic bone formation, includingbone surface (BS), total volume (TV), and Trabecular number (Tb. N), is presented. Scale bar = 1 mm. Data is reported as mean values ± standard deviation (n = 6). Statistical analysis was performed using ANOVA, with triple asterisks indicating high significance (***p < 0.01), and "ns" denoting non-significance. (**e and f**) Representative 3D micro-CT transverse position images depicting the second lumbar (L2) vertebra following a 2-week treatment with 50 mg/kg Sorafenib, an LOXL2 enzyme inhibitor [(2-Chloropyridin-4-yl)methanamine hydrochloride], and a methylcellulose carrier (Vehicle). Within the images, color-coded bars illustrate the density of ossified tissue, with higher density represented at the top and lower density at the bottom. Quantitative analysis of structural parameters pertaining to heterotopic bone formation, encompassing BV/TV, BS/TV, Trabecular number (Tb. N) and Trabecular Thickness (Tb.th), is presented. The scale bar corresponds to 0.5 mm. Data is expressed as mean values ± standard deviation (n = 6). Statistical analysis was conducted via ANOVA, with triple asterisks indicating a high level of significance (***p < 0.01), while "ns" signifies non-significance. (**g**) Representative images display Masson's and Sirius Red staining of murine liver and kidney tissues after interventions in distinct treatment groups. These interventions included the administration of 50 mg/kg Sorafenib, an LOXL2 enzyme inhibitor [(2-Chloropyridin-4-yl)methanamine hydrochloride], and a methylcellulose carrier as a control (Vehicle). The scale bar for representative images of kidney tissue is 100 μm, and the scale bar for representative images of liver tissue is 200 μm.

**
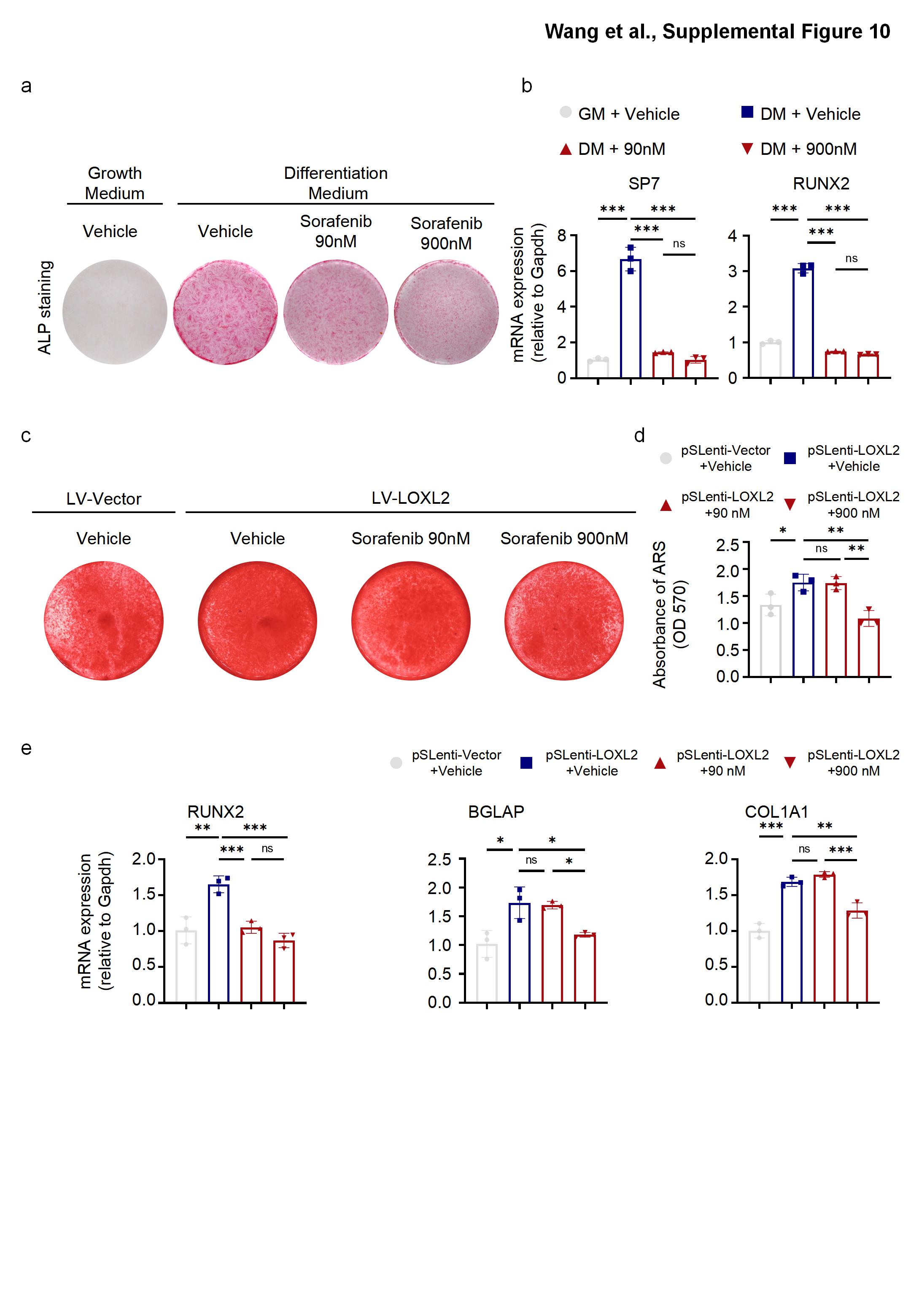
**

**Supplemental Figure 10. The effect Sorafenib on ligament cell osteogenic differentiation.**

**(a)** Alkaline Phosphatase (ALP) staining at 7 days, demonstrating the osteogenic differentiation potential of ligament cells under DMSO (Vehicle) and Sorafenib (90 nM and 900 nM). **(b)** Expression levels of *SP7* and *RUNX2* relative to *GAPDH* of ligament cells. Values are means ± s.d. n = 3. ***P < 0.001, ns not significant by Student's t-test. **(c and d)** Representative Alizarin Red S staining images (left) and quantification (right) for ligament cells infected with lentiviral vectors (LV-Vector) and LOXL2 virus (LV-LOXL2) treated with Sorafenib (90 nM, 900 nM) or DMSO (Vehicle). Values are means ± s.d. n = 3. **P < 0.01, *P < 0.05, ns not significant by Student's t-test. **(e)** Expression levels of *RUNX2*, *BGLAP* and *COL1A1* relative to *GAPDH* of ligament cells. Values are means ± s.d. n = 3. ***P < 0.001, **P < 0.01, *P < 0.05, ns not significant by Student’s t-test. The experiments were performed in three biological replicates.

**
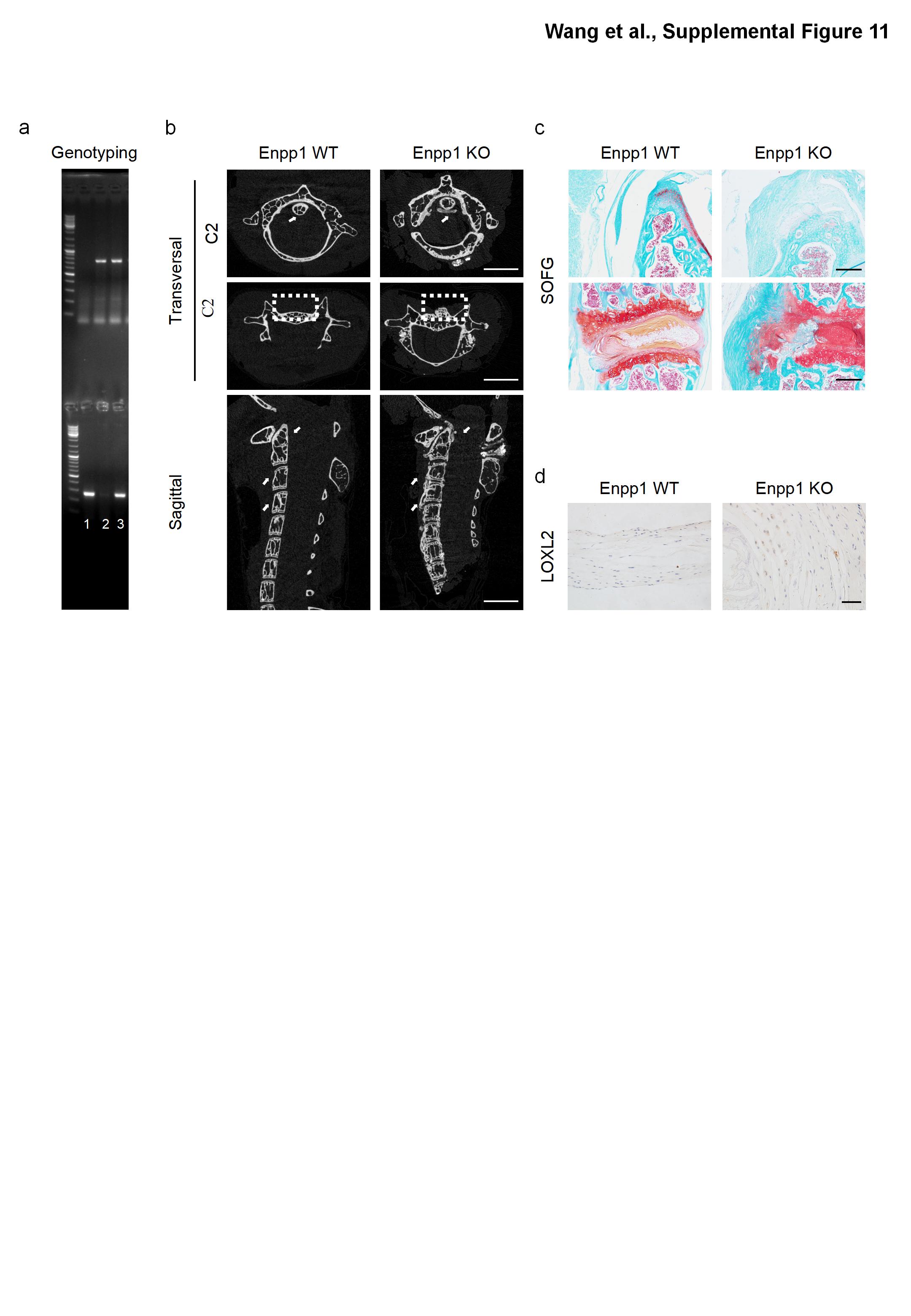
Supplemental Figure 11. Enpp1 knockout can induce spontaneous ligament ossification.**

**(a)** Genotyping of Enpp1^+/+^ (WT, 1), Enpp1^-/-^(KO, 2), and Enpp1^+/-^ (Heterozygote, 3) mice. **(b)** Representative microcomputed tomography (microCT) images of C2 and C5 cervical vertebrae in transverse and sagittal views for both wild-type (Enpp1 WT, left) and Enpp1 knockout (Enpp1 KO, right) mice. White arrows and white dotted boxes indicate ossification sites. Scale bar = 2 mm. n = 3. **(c)** Safranin O and Fast Green (SOFG) staining of spinal ligaments in C2 and C5 cervical vertebrae from Enpp1 WT (left) and Enpp1 KO (right) mice. Scale bar = 100 μm. n = 3. **(d)** Representative images of LOXL2 expression in the posterior longitudinal ligament of Enpp1 WT (left) and Enpp1 KO (right) mice. Scale bar = 50 μm. n = 3. Experiments were performed with three biological replicates.

**
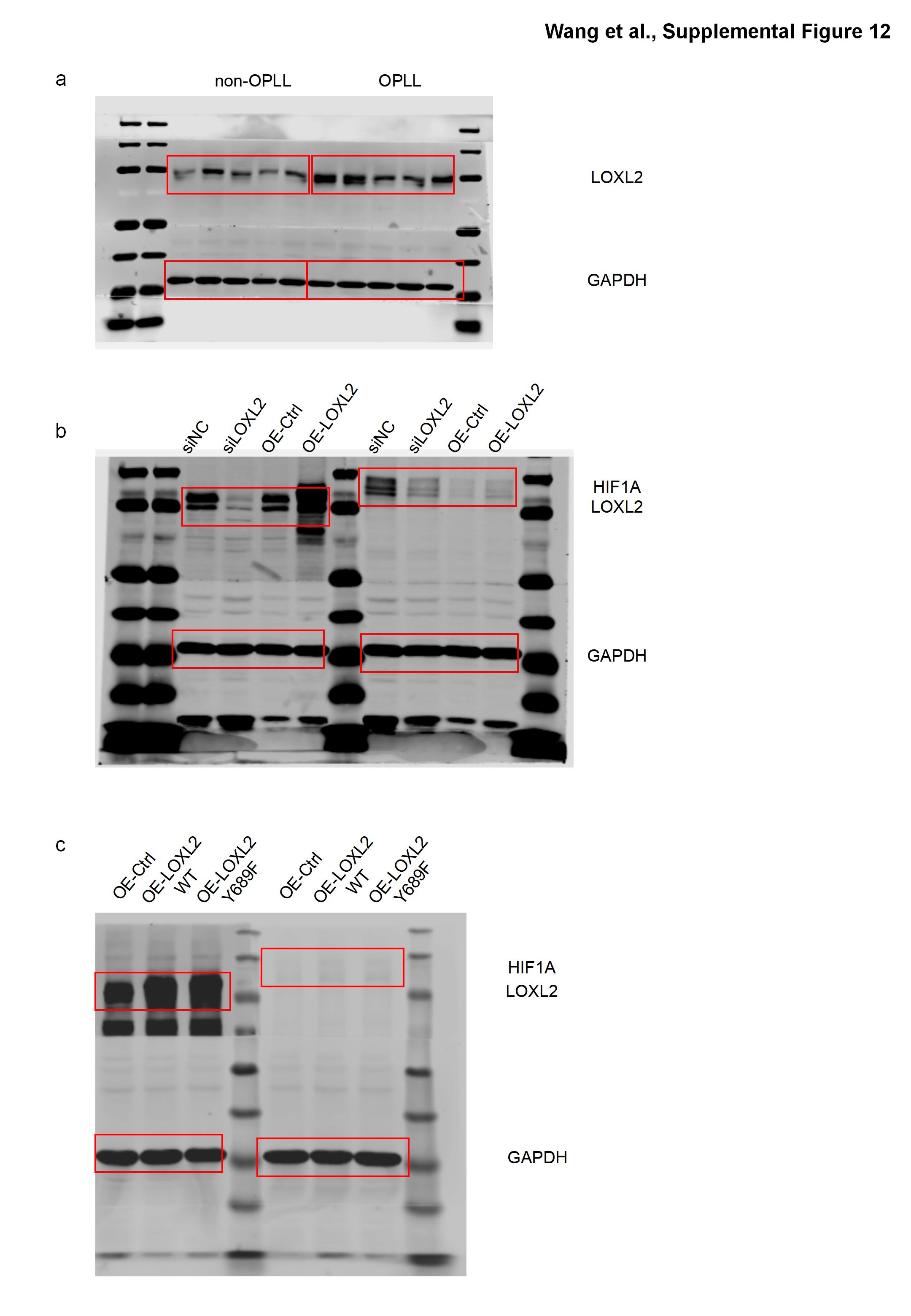
Supplemental Figure 12. Whole gel images for western blots.**

**(a)** Full unedited gel for Figure 2e. **(b)** Full unedited gel for Figure 5c and Supplemental Figure7d.**(c)** Full unedited gel for Figure 5j.

Table S1. OPLL vs non-OPLL GO signalling pathway enrichment analysis results

| ID | Description | GeneRatio | BgRatio | enrich factor | pvalue | qvalue | ontology |
| --- | --- | --- | --- | --- | --- | --- | --- |
| GO:0030198 | extracellular matrix organization | 51/521 | 358/14375 | 3.93 | 3.12E-17 | 6.93E-14 | BP |
| GO:0043062 | extracellular structure organization | 51/521 | 359/14375 | 3.92 | 3.52E-17 | 6.93E-14 | BP |
| GO:0032963 | collagen metabolic process | 21/521 | 95/14375 | 6.1 | 1.87E-11 | 2.46E-08 | BP |
| GO:0001501 | skeletal system development | 41/521 | 471/14375 | 2.4 | 1.89E-07 | 0.000186056 | BP |
| GO:0010817 | regulation of hormone levels | 36/521 | 413/14375 | 2.41 | 1.04E-06 | 0.000748449 | BP |
| GO:0030574 | collagen catabolic process | 10/521 | 40/14375 | 6.9 | 1.14E-06 | 0.000748449 | BP |
| GO:0001525 | angiogenesis | 39/521 | 472/14375 | 2.28 | 1.41E-06 | 0.000792624 | BP |
| GO:0030199 | collagen fibril organization | 11/521 | 52/14375 | 5.84 | 2.00E-06 | 0.000984241 | BP |
| GO:0042476 | odontogenesis | 16/521 | 114/14375 | 3.87 | 3.41E-06 | 0.001491915 | BP |
| GO:0061448 | connective tissue development | 24/521 | 237/14375 | 2.79 | 5.58E-06 | 0.0020266 | BP |
| GO:0009612 | response to mechanical stimulus | 21/521 | 191/14375 | 3.03 | 6.00E-06 | 0.0020266 | BP |
| GO:0001503 | ossification | 32/521 | 374/14375 | 2.36 | 6.18E-06 | 0.0020266 | BP |
| GO:0051216 | cartilage development | 20/521 | 178/14375 | 3.1 | 7.18E-06 | 0.002173797 | BP |
| GO:0061045 | negative regulation of wound healing | 11/521 | 61/14375 | 4.98 | 1.03E-05 | 0.002898099 | BP |
| GO:0051966 | regulation of synaptic transmission, glutamatergic | 11/521 | 62/14375 | 4.9 | 1.21E-05 | 0.003182352 | BP |
| GO:1903035 | negative regulation of response to wounding | 12/521 | 75/14375 | 4.41 | 1.48E-05 | 0.00362892 | BP |
| GO:0060348 | bone development | 20/521 | 190/14375 | 2.9 | 1.90E-05 | 0.004235781 | BP |
| GO:0023061 | signal release | 36/521 | 470/14375 | 2.11 | 1.94E-05 | 0.004235781 | BP |
| GO:0035249 | synaptic transmission, glutamatergic | 12/521 | 79/14375 | 4.19 | 2.54E-05 | 0.005251942 | BP |
| GO:0010544 | negative regulation of platelet activation | 6/521 | 18/14375 | 9.2 | 2.82E-05 | 0.005544998 | BP |
| GO:0007610 | behavior | 36/521 | 487/14375 | 2.04 | 4.11E-05 | 0.007706817 | BP |
| GO:0031214 | biomineral tissue development | 16/521 | 142/14375 | 3.11 | 5.65E-05 | 0.009038553 | BP |
| GO:0110148 | biomineralization | 16/521 | 142/14375 | 3.11 | 5.65E-05 | 0.009038553 | BP |
| GO:0060349 | bone morphogenesis | 12/521 | 86/14375 | 3.85 | 6.01E-05 | 0.009038553 | BP |
| GO:0001958 | endochondral ossification | 7/521 | 29/14375 | 6.66 | 6.15E-05 | 0.009038553 | BP |
| GO:0036075 | replacement ossification | 7/521 | 29/14375 | 6.66 | 6.15E-05 | 0.009038553 | BP |
| GO:0061687 | detoxification of inorganic compound | 5/521 | 13/14375 | 10.61 | 6.20E-05 | 0.009038553 | BP |
| GO:0060384 | innervation | 6/521 | 21/14375 | 7.88 | 7.51E-05 | 0.010551045 | BP |
| GO:0001706 | endoderm formation | 9/521 | 51/14375 | 4.87 | 7.87E-05 | 0.010686386 | BP |
| GO:0030195 | negative regulation of blood coagulation | 8/521 | 41/14375 | 5.38 | 9.38E-05 | 0.012299976 | BP |
| GO:1900047 | negative regulation of hemostasis | 8/521 | 42/14375 | 5.26 | 0.000112186 | 0.013816272 | BP |
| GO:0009914 | hormone transport | 23/521 | 267/14375 | 2.38 | 0.000112333 | 0.013816272 | BP |
| GO:0060350 | endochondral bone morphogenesis | 9/521 | 54/14375 | 4.6 | 0.000124979 | 0.014905846 | BP |
| GO:0031960 | response to corticosteroid | 15/521 | 138/14375 | 3 | 0.000143941 | 0.016662441 | BP |
| GO:0050819 | negative regulation of coagulation | 8/521 | 44/14375 | 5.02 | 0.000158071 | 0.017775315 | BP |
| GO:0007492 | endoderm development | 10/521 | 70/14375 | 3.94 | 0.000201395 | 0.022018056 | BP |
| GO:0001655 | urogenital system development | 24/521 | 297/14375 | 2.23 | 0.00021356 | 0.022237074 | BP |
| GO:0046879 | hormone secretion | 22/521 | 261/14375 | 2.33 | 0.000214699 | 0.022237074 | BP |
| GO:0071772 | response to BMP | 15/521 | 144/14375 | 2.87 | 0.000231156 | 0.022744561 | BP |
| GO:0071773 | cellular response to BMP stimulus | 15/521 | 144/14375 | 2.87 | 0.000231156 | 0.022744561 | BP |
| GO:0007600 | sensory perception | 29/521 | 395/14375 | 2.03 | 0.000256507 | 0.024623337 | BP |
| GO:0072507 | divalent inorganic cation homeostasis | 30/521 | 423/14375 | 1.96 | 0.000363669 | 0.034079131 | BP |
| GO:0006875 | cellular metal ion homeostasis | 33/521 | 485/14375 | 1.88 | 0.00039623 | 0.034910738 | BP |
| GO:0033273 | response to vitamin | 10/521 | 76/14375 | 3.63 | 0.000399153 | 0.034910738 | BP |
| GO:0042475 | odontogenesis of dentin-containing tooth | 10/521 | 76/14375 | 3.63 | 0.000399153 | 0.034910738 | BP |
| GO:0021675 | nerve development | 9/521 | 63/14375 | 3.94 | 0.000416717 | 0.035166575 | BP |
| GO:0071295 | cellular response to vitamin | 6/521 | 28/14375 | 5.91 | 0.000419949 | 0.035166575 | BP |
| GO:1903034 | regulation of response to wounding | 15/521 | 153/14375 | 2.71 | 0.000446123 | 0.036158331 | BP |
| GO:0010273 | detoxification of copper ion | 4/521 | 11/14375 | 10.03 | 0.000459353 | 0.036158331 | BP |
| GO:1990169 | stress response to copper ion | 4/521 | 11/14375 | 10.03 | 0.000459353 | 0.036158331 | BP |
| GO:1903532 | positive regulation of secretion by cell | 21/521 | 259/14375 | 2.24 | 0.000498741 | 0.038273876 | BP |
| GO:0032355 | response to estradiol | 13/521 | 123/14375 | 2.92 | 0.000516437 | 0.038273876 | BP |
| GO:0051384 | response to glucocorticoid | 13/521 | 123/14375 | 2.92 | 0.000516437 | 0.038273876 | BP |
| GO:0048545 | response to steroid hormone | 23/521 | 297/14375 | 2.14 | 0.000525127 | 0.038273876 | BP |
| GO:0061035 | regulation of cartilage development | 9/521 | 66/14375 | 3.76 | 0.000592034 | 0.042365815 | BP |
| GO:0061041 | regulation of wound healing | 13/521 | 125/14375 | 2.87 | 0.000603 | 0.042380046 | BP |
| GO:0050808 | synapse organization | 27/521 | 381/14375 | 1.96 | 0.000714911 | 0.048446679 | BP |
| GO:1901654 | response to ketone | 16/521 | 177/14375 | 2.49 | 0.000717448 | 0.048446679 | BP |
| GO:0045926 | negative regulation of growth | 19/521 | 230/14375 | 2.28 | 0.000726247 | 0.048446679 | BP |
| GO:0003203 | endocardial cushion morphogenesis | 6/521 | 31/14375 | 5.34 | 0.000748442 | 0.049095172 | BP |
| GO:0031012 | extracellular matrix | 72/542 | 491/14976 | 4.05 | 8.27E-25 | 2.97E-22 | CC |
| GO:0062023 | collagen-containing extracellular matrix | 50/542 | 381/14976 | 3.63 | 1.96E-15 | 3.52E-13 | CC |
| GO:0005788 | endoplasmic reticulum lumen | 40/542 | 273/14976 | 4.05 | 3.67E-14 | 4.39E-12 | CC |
| GO:0005581 | collagen trimer | 17/542 | 78/14976 | 6.02 | 1.98E-09 | 1.77E-07 | CC |
| GO:0005604 | basement membrane | 18/542 | 97/14976 | 5.13 | 1.01E-08 | 7.25E-07 | CC |
| GO:0098644 | complex of collagen trimers | 8/542 | 20/14976 | 11.05 | 2.40E-07 | 1.43E-05 | CC |
| GO:0009897 | external side of plasma membrane | 32/542 | 345/14976 | 2.56 | 1.09E-06 | 5.59E-05 | CC |
| GO:0005583 | fibrillar collagen trimer | 5/542 | 12/14976 | 11.51 | 3.91E-05 | 0.001558702 | CC |
| GO:0098643 | banded collagen fibril | 5/542 | 12/14976 | 11.51 | 3.91E-05 | 0.001558702 | CC |
| GO:0005796 | Golgi lumen | 11/542 | 76/14976 | 4 | 8.56E-05 | 0.003072132 | CC |
| GO:0031225 | anchored component of membrane | 14/542 | 128/14976 | 3.02 | 0.000220177 | 0.007184729 | CC |
| GO:0099572 | postsynaptic specialization | 24/542 | 322/14976 | 2.06 | 0.000682179 | 0.020405536 | CC |
| GO:0045211 | postsynaptic membrane | 18/542 | 214/14976 | 2.32 | 0.000795776 | 0.021972431 | CC |
| GO:0099634 | postsynaptic specialization membrane | 10/542 | 87/14976 | 3.18 | 0.001162908 | 0.029806054 | CC |
| GO:0045177 | apical part of cell | 25/542 | 356/14976 | 1.94 | 0.001245561 | 0.029806054 | CC |
| GO:0014069 | postsynaptic density | 22/542 | 308/14976 | 1.97 | 0.001932403 | 0.043351944 | CC |
| GO:0032279 | asymmetric synapse | 22/542 | 312/14976 | 1.95 | 0.002268461 | 0.047897535 | CC |
| GO:0016324 | apical plasma membrane | 21/542 | 294/14976 | 1.97 | 0.002423567 | 0.048329609 | CC |
| GO:0005201 | extracellular matrix structural constituent | 26/510 | 148/14651 | 5.05 | 7.98E-12 | 4.78E-09 | MF |
| GO:0030020 | extracellular matrix structural constituent conferring tensile strength | 12/510 | 40/14651 | 8.62 | 6.39E-09 | 1.91E-06 | MF |
| GO:0005539 | glycosaminoglycan binding | 23/510 | 194/14651 | 3.41 | 2.87E-07 | 5.73E-05 | MF |
| GO:0004222 | metalloendopeptidase activity | 15/510 | 89/14651 | 4.84 | 3.87E-07 | 5.79E-05 | MF |
| GO:0019838 | growth factor binding | 18/510 | 129/14651 | 4.01 | 5.13E-07 | 6.15E-05 | MF |
| GO:0048407 | platelet-derived growth factor binding | 6/510 | 11/14651 | 15.67 | 6.88E-07 | 6.87E-05 | MF |
| GO:0005518 | collagen binding | 11/510 | 66/14651 | 4.79 | 1.55E-05 | 0.001324643 | MF |
| GO:0004930 | G protein-coupled receptor activity | 25/510 | 306/14651 | 2.35 | 7.09E-05 | 0.004379553 | MF |
| GO:0015267 | channel activity | 27/510 | 344/14651 | 2.25 | 7.31E-05 | 0.004379553 | MF |
| GO:0022803 | passive transmembrane transporter activity | 27/510 | 344/14651 | 2.25 | 7.31E-05 | 0.004379553 | MF |
| GO:0005178 | integrin binding | 15/510 | 138/14651 | 3.12 | 9.24E-05 | 0.00497287 | MF |
| GO:0005216 | ion channel activity | 25/510 | 314/14651 | 2.29 | 0.0001072 | 0.00497287 | MF |
| GO:0030545 | receptor regulator activity | 26/510 | 333/14651 | 2.24 | 0.000107935 | 0.00497287 | MF |
| GO:0008237 | metallopeptidase activity | 16/510 | 158/14651 | 2.91 | 0.000126915 | 0.005429653 | MF |
| GO:0008201 | heparin binding | 15/510 | 145/14651 | 2.97 | 0.000161529 | 0.006449826 | MF |
| GO:0022836 | gated channel activity | 20/510 | 236/14651 | 2.43 | 0.00022769 | 0.008523377 | MF |
| GO:0030546 | signaling receptor activator activity | 24/510 | 312/14651 | 2.21 | 0.000246107 | 0.008670881 | MF |
| GO:0048018 | receptor ligand activity | 23/510 | 307/14651 | 2.15 | 0.000478318 | 0.015915969 | MF |
| GO:0001228 | DNA-binding transcription activator activity, RNA polymerase II-specific | 28/510 | 410/14651 | 1.96 | 0.0005471 | 0.017246519 | MF |
| GO:0001216 | DNA-binding transcription activator activity | 28/510 | 413/14651 | 1.95 | 0.000613244 | 0.018365057 | MF |
| GO:0008528 | G protein-coupled peptide receptor activity | 11/510 | 102/14651 | 3.1 | 0.000833244 | 0.023765198 | MF |
| GO:0004175 | endopeptidase activity | 23/510 | 322/14651 | 2.05 | 0.000920607 | 0.025063412 | MF |
| GO:0008188 | neuropeptide receptor activity | 5/510 | 23/14651 | 6.25 | 0.001002156 | 0.026064139 | MF |
| GO:0001653 | peptide receptor activity | 11/510 | 105/14651 | 3.01 | 0.001061489 | 0.026064139 | MF |
| GO:0042923 | neuropeptide binding | 4/510 | 14/14651 | 8.21 | 0.001099573 | 0.026064139 | MF |
| GO:0033218 | amide binding | 23/510 | 327/14651 | 2.02 | 0.001131431 | 0.026064139 | MF |
| GO:1901681 | sulfur compound binding | 18/510 | 231/14651 | 2.24 | 0.001228937 | 0.027261798 | MF |
| GO:0008191 | metalloendopeptidase inhibitor activity | 4/510 | 15/14651 | 7.66 | 0.00145846 | 0.030122086 | MF |
| GO:0016722 | oxidoreductase activity, oxidizing metal ions | 4/510 | 15/14651 | 7.66 | 0.00145846 | 0.030122086 | MF |
| GO:0042277 | peptide binding | 19/510 | 260/14651 | 2.1 | 0.001924019 | 0.038412877 | MF |
| GO:0005261 | cation channel activity | 18/510 | 242/14651 | 2.14 | 0.002064269 | 0.039883509 | MF |
| GO:0005254 | chloride channel activity | 7/510 | 53/14651 | 3.79 | 0.002288135 | 0.042827257 | MF |
| GO:0071855 | neuropeptide receptor binding | 4/510 | 17/14651 | 6.76 | 0.002406173 | 0.043671854 | MF |
| GO:0042562 | hormone binding | 8/510 | 70/14651 | 3.28 | 0.002896765 | 0.049790127 | MF |
| GO:0019955 | cytokine binding | 11/510 | 119/14651 | 2.66 | 0.002909529 | 0.049790127 | MF |

Table S2. OPLL vs non-OPLL GSEA enrichment analysis results

| Description | setSize | enrichmentScore | NES | p.adjust | rank |
| --- | --- | --- | --- | --- | --- |
| HALLMARK_ALLOGRAFT_REJECTION | 186 | 0.61599068 | 2.321571065 | 2.50E-09 | 2910 |
| HALLMARK_EPITHELIAL_MESENCHYMAL_TRANSITION | 198 | 0.554325803 | 2.095051442 | 2.50E-09 | 3473 |
| HALLMARK_COMPLEMENT | 192 | 0.5104621 | 1.924158888 | 3.40E-07 | 4003 |
| HALLMARK_INFLAMMATORY_RESPONSE | 185 | 0.5101943 | 1.922921754 | 4.96E-07 | 3222 |
| HALLMARK_KRAS_SIGNALING_UP | 184 | 0.509468099 | 1.916908608 | 8.66E-07 | 2774 |
| HALLMARK_IL2_STAT5_SIGNALING | 197 | 0.44831947 | 1.694906559 | 0.000238236 | 3802 |
| HALLMARK_COAGULATION | 118 | 0.485700325 | 1.734076155 | 0.001009527 | 3010 |
| HALLMARK_G2M_CHECKPOINT | 199 | 0.427894026 | 1.619410299 | 0.001138691 | 4291 |
| HALLMARK_INTERFERON_GAMMA_RESPONSE | 197 | 0.428624632 | 1.620448695 | 0.001138691 | 4370 |
| HALLMARK_E2F_TARGETS | 199 | 0.422915199 | 1.600567398 | 0.001503351 | 5183 |
| HALLMARK_ANGIOGENESIS | 35 | 0.638475843 | 1.886826921 | 0.001569135 | 2148 |
| HALLMARK_APICAL_JUNCTION | 188 | 0.414882396 | 1.564193241 | 0.003145089 | 3648 |

Table S3. PPI network hub gene scores of different computational method

| Rank | Computational Method | | | | |
| --- | --- | --- | --- | --- | --- |
|  | MCC | DMNC | MNC | EPC | ClusteringCoefficient |
| 1 | COL1A1 | LOXL2 | COL1A1 | COL1A1 | CDCP1 |
| 2 | COL1A2 | PCOLCE | COL1A2 | COL1A2 | CCNA2 |
| 3 | COL3A1 | COL13A1 | COL3A1 | COL3A1 | LOXL2 |
| 4 | COL5A1 | LEPREL1 | COL5A1 | COL5A1 | F2RL2 |
| 5 | PCOLCE | TGFBI | MMP14 | MMP14 | LEPREL1 |
| 6 | SERPINH1 | F2RL2 | CDH2 | SERPINH1 | NEGR1 |
| 7 | BMP1 | SERPINH1 | COL6A3 | BMP1 | PRND |
| 8 | LOXL2 | BMP1 | SERPINH1 | COL6A3 | CREB3L1 |
| 9 | COL6A3 | ADAM12 | BMP1 | SDC1 | OLFML2B |
| 10 | ADAMTS2 | WISP1 | ADAMTS2 | PCOLCE | CCL19 |
| 11 | COL13A1 | SPON2 | SDC1 | MMP13 | HCAR1 |
| 12 | MMP14 | ADAMTS7 | MMP13 | SP7 | ADAMTSL4 |
| 13 | SDC1 | SULF1 | THY1 | ADAMTS2 | ZFP36 |
| 14 | ADAMTS14 | ADAMTSL4 | PCOLCE | CDH2 | NFKBIZ |
| 15 | LEPREL1 | COL5A1 | SP7 | THY1 | DHRS3 |
| 16 | TGFBI | COL6A3 | ADAMTS14 | COL13A1 | COL13A1 |
| 17 | PIK3R1 | ADAMTS2 | ALPL | MMP1 | TGFBI |
| 18 | F2 | BDKRB2 | AR | LOXL2 | PCOLCE |
| 19 | GPR68 | P2RY6 | MMP1 | ADAMTS14 | FAP |
| 20 | BDKRB2 | FAP | GPR68 | ALPL | P2RY6 |
| 21 | P2RY6 | ADAMTS14 | LOXL2 | F2 | TF |
| 22 | F2RL2 | COL3A1 | COL13A1 | ADAM12 | PGF |
| 23 | MMP13 | SDC1 | PIK3R1 | LEPREL1 | SPON2 |
| 24 | SP7 | DLX3 | F2 | TGFBI | SULF1 |
| 25 | MMP1 | AMTN | MET | PIK3R1 | ADAMTS7 |

Table S4. Hypertrophy vs non-hypertrophy ligamentum flavum GSEA enrichment analysis results

| Description | setSize | enrichmentScore | NES | p.adjust | rank |
| --- | --- | --- | --- | --- | --- |
| HALLMARK_EPITHELIAL_MESENCHYMAL_TRANSITION | 194 | 0.645424626 | 2.708675756 | 2.50E-09 | 2198 |
| HALLMARK_MYOGENESIS | 197 | -0.612210518 | -2.5693266 | 2.50E-09 | 1287 |
| HALLMARK_INFLAMMATORY_RESPONSE | 196 | 0.471346707 | 1.97988382 | 2.25E-07 | 3191 |
| HALLMARK_COAGULATION | 135 | 0.471302611 | 1.893188892 | 0.000171921 | 2714 |
| HALLMARK_ADIPOGENESIS | 187 | -0.430696117 | -1.782003423 | 0.000195013 | 3132 |
| HALLMARK_GLYCOLYSIS | 193 | 0.407628135 | 1.711616676 | 0.000701841 | 3550 |
| HALLMARK_ALLOGRAFT_REJECTION | 193 | 0.402662582 | 1.690766487 | 0.000793639 | 3477 |
| HALLMARK_KRAS_SIGNALING_UP | 192 | 0.397379907 | 1.667009815 | 0.000793639 | 2125 |
| HALLMARK_KRAS_SIGNALING_DN | 184 | -0.407355586 | -1.678625784 | 0.000828681 | 2202 |
| HALLMARK_P53_PATHWAY | 191 | 0.389313257 | 1.632210055 | 0.003121692 | 3648 |
| HALLMARK_IL2_STAT5_SIGNALING | 193 | 0.380884295 | 1.599320202 | 0.003417058 | 4218 |
| HALLMARK_ANGIOGENESIS | 35 | 0.579770213 | 1.810612099 | 0.004669696 | 1813 |
| HALLMARK_UNFOLDED_PROTEIN_RESPONSE | 105 | 0.420443049 | 1.613059618 | 0.009383931 | 4415 |
| HALLMARK_TGF_BETA_SIGNALING | 54 | 0.491364973 | 1.668916991 | 0.012671819 | 2936 |
| HALLMARK_FATTY_ACID_METABOLISM | 150 | -0.379783443 | -1.522811332 | 0.012774161 | 4057 |
| HALLMARK_TNFA_SIGNALING_VIA_NFKB | 197 | 0.341591882 | 1.437164972 | 0.015989716 | 3646 |
| HALLMARK_IL6_JAK_STAT3_SIGNALING | 87 | 0.41716226 | 1.541379001 | 0.018670228 | 2329 |
| HALLMARK_COMPLEMENT | 193 | 0.346410059 | 1.454564056 | 0.022139996 | 3528 |
| HALLMARK_HYPOXIA | 189 | 0.337688694 | 1.416731159 | 0.023484385 | 2610 |
| HALLMARK_MTORC1_SIGNALING | 186 | 0.347556563 | 1.458611086 | 0.039606259 | 4719 |
| HALLMARK_APOPTOSIS | 152 | 0.342533525 | 1.397071628 | 0.045608069 | 3201 |

Table S5. OPLL vs non-OPLL derived cells GO signalling pathway enrichment analysis results

| ID | Description | GeneRatio | BgRatio | enrich_factor | pvalue | qvalue | ontology |
| --- | --- | --- | --- | --- | --- | --- | --- |
| GO:0030198 | extracellular matrix organization | 149/2872 | 319/13036 | 2.12 | 4.65E-23 | 9.64E-20 | BP |
| GO:0043062 | extracellular structure organization | 149/2872 | 319/13036 | 2.12 | 4.65E-23 | 9.64E-20 | BP |
| GO:0001501 | skeletal system development | 174/2872 | 419/13036 | 1.88 | 7.21E-20 | 9.96E-17 | BP |
| GO:0044057 | regulation of system process | 158/2872 | 396/13036 | 1.81 | 3.14E-16 | 3.25E-13 | BP |
| GO:0008015 | blood circulation | 146/2872 | 366/13036 | 1.81 | 4.46E-15 | 3.70E-12 | BP |
| GO:0003013 | circulatory system process | 150/2872 | 383/13036 | 1.78 | 1.16E-14 | 8.03E-12 | BP |
| GO:0006935 | chemotaxis | 171/2872 | 467/13036 | 1.66 | 1.97E-13 | 1.16E-10 | BP |
| GO:0042330 | taxis | 171/2872 | 469/13036 | 1.65 | 3.02E-13 | 1.57E-10 | BP |
| GO:0003012 | muscle system process | 125/2872 | 314/13036 | 1.81 | 5.15E-13 | 2.37E-10 | BP |
| GO:0019932 | second-messenger-mediated signaling | 118/2872 | 292/13036 | 1.83 | 7.02E-13 | 2.69E-10 | BP |
| GO:0048598 | embryonic morphogenesis | 167/2872 | 459/13036 | 1.65 | 7.13E-13 | 2.69E-10 | BP |
| GO:0072001 | renal system development | 100/2872 | 235/13036 | 1.93 | 1.03E-12 | 3.57E-10 | BP |
| GO:0003018 | vascular process in circulatory system | 66/2872 | 132/13036 | 2.27 | 1.23E-12 | 3.94E-10 | BP |
| GO:0001503 | ossification | 128/2872 | 336/13036 | 1.73 | 1.04E-11 | 3.09E-09 | BP |
| GO:0098742 | cell-cell adhesion via plasma-membrane adhesion molecules | 82/2872 | 186/13036 | 2 | 1.28E-11 | 3.53E-09 | BP |
| GO:0030855 | epithelial cell differentiation | 165/2872 | 467/13036 | 1.6 | 1.55E-11 | 3.96E-09 | BP |
| GO:0001822 | kidney development | 95/2872 | 228/13036 | 1.89 | 1.63E-11 | 3.96E-09 | BP |
| GO:0055074 | calcium ion homeostasis | 118/2872 | 305/13036 | 1.76 | 2.13E-11 | 4.78E-09 | BP |
| GO:0006936 | muscle contraction | 99/2872 | 242/13036 | 1.86 | 2.19E-11 | 4.78E-09 | BP |
| GO:0019935 | cyclic-nucleotide-mediated signaling | 64/2872 | 133/13036 | 2.18 | 2.34E-11 | 4.84E-09 | BP |
| GO:0055065 | metal ion homeostasis | 158/2872 | 446/13036 | 1.61 | 3.36E-11 | 6.63E-09 | BP |
| GO:0072507 | divalent inorganic cation homeostasis | 126/2872 | 335/13036 | 1.71 | 4.12E-11 | 7.77E-09 | BP |
| GO:0001655 | urogenital system development | 105/2872 | 265/13036 | 1.8 | 5.30E-11 | 8.58E-09 | BP |
| GO:0035150 | regulation of tube size | 49/2872 | 92/13036 | 2.42 | 5.38E-11 | 8.58E-09 | BP |
| GO:0035296 | regulation of tube diameter | 49/2872 | 92/13036 | 2.42 | 5.38E-11 | 8.58E-09 | BP |
| GO:0097746 | regulation of blood vessel diameter | 49/2872 | 92/13036 | 2.42 | 5.38E-11 | 8.58E-09 | BP |
| GO:0001763 | morphogenesis of a branching structure | 72/2872 | 160/13036 | 2.04 | 7.12E-11 | 1.09E-08 | BP |
| GO:0006874 | cellular calcium ion homeostasis | 115/2872 | 300/13036 | 1.74 | 7.54E-11 | 1.12E-08 | BP |
| GO:0001525 | angiogenesis | 152/2872 | 429/13036 | 1.61 | 7.91E-11 | 1.13E-08 | BP |
| GO:0072503 | cellular divalent inorganic cation homeostasis | 122/2872 | 325/13036 | 1.7 | 9.76E-11 | 1.35E-08 | BP |
| GO:0048705 | skeletal system morphogenesis | 79/2872 | 183/13036 | 1.96 | 1.07E-10 | 1.43E-08 | BP |
| GO:0051480 | regulation of cytosolic calcium ion concentration | 92/2872 | 226/13036 | 1.85 | 1.53E-10 | 1.98E-08 | BP |
| GO:0060326 | cell chemotaxis | 85/2872 | 204/13036 | 1.89 | 1.90E-10 | 2.38E-08 | BP |
| GO:0060348 | bone development | 77/2872 | 179/13036 | 1.95 | 2.25E-10 | 2.74E-08 | BP |
| GO:0050878 | regulation of body fluid levels | 133/2872 | 368/13036 | 1.64 | 2.77E-10 | 3.28E-08 | BP |
| GO:0097756 | negative regulation of blood vessel diameter | 33/2872 | 53/13036 | 2.83 | 3.16E-10 | 3.64E-08 | BP |
| GO:0001823 | mesonephros development | 44/2872 | 82/13036 | 2.44 | 3.74E-10 | 4.13E-08 | BP |
| GO:0051216 | cartilage development | 73/2872 | 168/13036 | 1.97 | 3.79E-10 | 4.13E-08 | BP |
| GO:0099537 | trans-synaptic signaling | 167/2872 | 492/13036 | 1.54 | 4.17E-10 | 4.44E-08 | BP |
| GO:0007423 | sensory organ development | 145/2872 | 413/13036 | 1.59 | 4.53E-10 | 4.70E-08 | BP |
| GO:0006939 | smooth muscle contraction | 42/2872 | 77/13036 | 2.48 | 4.75E-10 | 4.71E-08 | BP |
| GO:0006875 | cellular metal ion homeostasis | 141/2872 | 399/13036 | 1.6 | 4.77E-10 | 4.71E-08 | BP |
| GO:0048562 | embryonic organ morphogenesis | 87/2872 | 214/13036 | 1.85 | 5.21E-10 | 4.82E-08 | BP |
| GO:0007268 | chemical synaptic transmission | 165/2872 | 486/13036 | 1.54 | 5.23E-10 | 4.82E-08 | BP |
| GO:0098916 | anterograde trans-synaptic signaling | 165/2872 | 486/13036 | 1.54 | 5.23E-10 | 4.82E-08 | BP |
| GO:0043269 | regulation of ion transport | 157/2872 | 458/13036 | 1.56 | 6.33E-10 | 5.71E-08 | BP |
| GO:0090596 | sensory organ morphogenesis | 82/2872 | 199/13036 | 1.87 | 7.55E-10 | 6.53E-08 | BP |
| GO:0031589 | cell-substrate adhesion | 113/2872 | 303/13036 | 1.69 | 7.56E-10 | 6.53E-08 | BP |
| GO:0007204 | positive regulation of cytosolic calcium ion concentration | 83/2872 | 203/13036 | 1.86 | 9.35E-10 | 7.91E-08 | BP |
| GO:0034341 | response to interferon-gamma | 66/2872 | 149/13036 | 2.01 | 9.90E-10 | 7.91E-08 | BP |
| GO:0061138 | morphogenesis of a branching epithelium | 66/2872 | 149/13036 | 2.01 | 9.90E-10 | 7.91E-08 | BP |
| GO:0033002 | muscle cell proliferation | 73/2872 | 171/13036 | 1.94 | 9.93E-10 | 7.91E-08 | BP |
| GO:0061448 | connective tissue development | 89/2872 | 223/13036 | 1.81 | 1.01E-09 | 7.91E-08 | BP |
| GO:0045165 | cell fate commitment | 71/2872 | 165/13036 | 1.95 | 1.11E-09 | 8.49E-08 | BP |
| GO:0042310 | vasoconstriction | 31/2872 | 50/13036 | 2.81 | 1.27E-09 | 9.59E-08 | BP |
| GO:0048568 | embryonic organ development | 122/2872 | 337/13036 | 1.64 | 1.36E-09 | 1.00E-07 | BP |
| GO:0016125 | sterol metabolic process | 60/2872 | 132/13036 | 2.06 | 1.67E-09 | 1.21E-07 | BP |
| GO:0030335 | positive regulation of cell migration | 153/2872 | 449/13036 | 1.55 | 1.69E-09 | 1.21E-07 | BP |
| GO:0042060 | wound healing | 138/2872 | 395/13036 | 1.59 | 1.74E-09 | 1.22E-07 | BP |
| GO:0043583 | ear development | 67/2872 | 154/13036 | 1.97 | 1.86E-09 | 1.27E-07 | BP |
| GO:0060485 | mesenchyme development | 91/2872 | 232/13036 | 1.78 | 1.87E-09 | 1.27E-07 | BP |
| GO:0072088 | nephron epithelium morphogenesis | 33/2872 | 56/13036 | 2.67 | 2.43E-09 | 1.60E-07 | BP |
| GO:0040017 | positive regulation of locomotion | 159/2872 | 473/13036 | 1.53 | 2.43E-09 | 1.60E-07 | BP |
| GO:0008202 | steroid metabolic process | 94/2872 | 244/13036 | 1.75 | 3.02E-09 | 1.96E-07 | BP |
| GO:0050731 | positive regulation of peptidyl-tyrosine phosphorylation | 62/2872 | 140/13036 | 2.01 | 3.20E-09 | 2.04E-07 | BP |
| GO:0001657 | ureteric bud development | 41/2872 | 78/13036 | 2.39 | 3.34E-09 | 2.10E-07 | BP |
| GO:2000147 | positive regulation of cell motility | 156/2872 | 464/13036 | 1.53 | 3.41E-09 | 2.11E-07 | BP |
| GO:0045785 | positive regulation of cell adhesion | 118/2872 | 329/13036 | 1.63 | 4.74E-09 | 2.89E-07 | BP |
| GO:0009611 | response to wounding | 161/2872 | 485/13036 | 1.51 | 5.27E-09 | 3.17E-07 | BP |
| GO:0072163 | mesonephric epithelium development | 41/2872 | 79/13036 | 2.36 | 5.48E-09 | 3.20E-07 | BP |
| GO:0072164 | mesonephric tubule development | 41/2872 | 79/13036 | 2.36 | 5.48E-09 | 3.20E-07 | BP |
| GO:0030278 | regulation of ossification | 70/2872 | 167/13036 | 1.9 | 5.56E-09 | 3.20E-07 | BP |
| GO:1903522 | regulation of blood circulation | 77/2872 | 190/13036 | 1.84 | 6.02E-09 | 3.42E-07 | BP |
| GO:0043588 | skin development | 80/2872 | 200/13036 | 1.82 | 6.23E-09 | 3.49E-07 | BP |
| GO:0009612 | response to mechanical stimulus | 71/2872 | 171/13036 | 1.88 | 7.01E-09 | 3.88E-07 | BP |
| GO:0030282 | bone mineralization | 46/2872 | 94/13036 | 2.22 | 7.54E-09 | 4.12E-07 | BP |
| GO:0072028 | nephron morphogenesis | 33/2872 | 58/13036 | 2.58 | 8.33E-09 | 4.49E-07 | BP |
| GO:0043410 | positive regulation of MAPK cascade | 140/2872 | 411/13036 | 1.55 | 8.65E-09 | 4.60E-07 | BP |
| GO:0006873 | cellular ion homeostasis | 156/2872 | 470/13036 | 1.51 | 9.26E-09 | 4.86E-07 | BP |
| GO:0051272 | positive regulation of cellular component movement | 158/2872 | 478/13036 | 1.5 | 1.02E-08 | 5.30E-07 | BP |
| GO:0019933 | cAMP-mediated signaling | 53/2872 | 116/13036 | 2.07 | 1.18E-08 | 6.04E-07 | BP |
| GO:0007200 | phospholipase C-activating G protein-coupled receptor signaling pathway | 30/2872 | 51/13036 | 2.67 | 1.37E-08 | 6.94E-07 | BP |
| GO:0007187 | G protein-coupled receptor signaling pathway, coupled to cyclic nucleotide second messenger | 58/2872 | 132/13036 | 1.99 | 1.45E-08 | 7.24E-07 | BP |
| GO:0030003 | cellular cation homeostasis | 153/2872 | 462/13036 | 1.5 | 1.53E-08 | 7.53E-07 | BP |
| GO:0008203 | cholesterol metabolic process | 54/2872 | 120/13036 | 2.04 | 1.66E-08 | 8.09E-07 | BP |
| GO:0072078 | nephron tubule morphogenesis | 31/2872 | 54/13036 | 2.61 | 1.76E-08 | 8.50E-07 | BP |
| GO:0009617 | response to bacterium | 136/2872 | 401/13036 | 1.54 | 1.92E-08 | 9.15E-07 | BP |
| GO:0072171 | mesonephric tubule morphogenesis | 29/2872 | 49/13036 | 2.69 | 1.98E-08 | 9.31E-07 | BP |
| GO:0002521 | leukocyte differentiation | 132/2872 | 387/13036 | 1.55 | 2.11E-08 | 9.84E-07 | BP |
| GO:0061333 | renal tubule morphogenesis | 32/2872 | 57/13036 | 2.55 | 2.18E-08 | 1.01E-06 | BP |
| GO:0048660 | regulation of smooth muscle cell proliferation | 54/2872 | 121/13036 | 2.03 | 2.36E-08 | 1.08E-06 | BP |
| GO:0030595 | leukocyte chemotaxis | 63/2872 | 150/13036 | 1.91 | 2.90E-08 | 1.31E-06 | BP |
| GO:0007507 | heart development | 151/2872 | 459/13036 | 1.49 | 3.09E-08 | 1.38E-06 | BP |
| GO:0072006 | nephron development | 50/2872 | 110/13036 | 2.06 | 3.74E-08 | 1.65E-06 | BP |
| GO:0042476 | odontogenesis | 44/2872 | 92/13036 | 2.17 | 3.81E-08 | 1.66E-06 | BP |
| GO:0061326 | renal tubule development | 36/2872 | 69/13036 | 2.37 | 3.88E-08 | 1.67E-06 | BP |
| GO:0042391 | regulation of membrane potential | 104/2872 | 290/13036 | 1.63 | 3.90E-08 | 1.67E-06 | BP |
| GO:0048762 | mesenchymal cell differentiation | 76/2872 | 194/13036 | 1.78 | 4.24E-08 | 1.78E-06 | BP |
| GO:0060993 | kidney morphogenesis | 37/2872 | 72/13036 | 2.33 | 4.26E-08 | 1.78E-06 | BP |
| GO:1902652 | secondary alcohol metabolic process | 56/2872 | 129/13036 | 1.97 | 4.29E-08 | 1.78E-06 | BP |
| GO:0048659 | smooth muscle cell proliferation | 54/2872 | 123/13036 | 1.99 | 4.68E-08 | 1.90E-06 | BP |
| GO:0048754 | branching morphogenesis of an epithelial tube | 54/2872 | 123/13036 | 1.99 | 4.68E-08 | 1.90E-06 | BP |
| GO:0060675 | ureteric bud morphogenesis | 28/2872 | 48/13036 | 2.65 | 5.38E-08 | 2.17E-06 | BP |
| GO:0001658 | branching involved in ureteric bud morphogenesis | 26/2872 | 43/13036 | 2.74 | 5.80E-08 | 2.31E-06 | BP |
| GO:0050804 | modulation of chemical synaptic transmission | 116/2872 | 335/13036 | 1.57 | 6.04E-08 | 2.38E-06 | BP |
| GO:0008544 | epidermis development | 87/2872 | 233/13036 | 1.69 | 6.31E-08 | 2.47E-06 | BP |
| GO:0051046 | regulation of secretion | 158/2872 | 490/13036 | 1.46 | 6.57E-08 | 2.55E-06 | BP |
| GO:0099177 | regulation of trans-synaptic signaling | 116/2872 | 336/13036 | 1.57 | 7.26E-08 | 2.79E-06 | BP |
| GO:0071346 | cellular response to interferon-gamma | 57/2872 | 134/13036 | 1.93 | 7.72E-08 | 2.94E-06 | BP |
| GO:0034329 | cell junction assembly | 118/2872 | 344/13036 | 1.56 | 8.36E-08 | 3.15E-06 | BP |
| GO:0090066 | regulation of anatomical structure size | 137/2872 | 414/13036 | 1.5 | 9.16E-08 | 3.42E-06 | BP |
| GO:0072080 | nephron tubule development | 35/2872 | 68/13036 | 2.34 | 9.34E-08 | 3.46E-06 | BP |
| GO:0031214 | biomineral tissue development | 52/2872 | 119/13036 | 1.98 | 9.95E-08 | 3.62E-06 | BP |
| GO:0110148 | biomineralization | 52/2872 | 119/13036 | 1.98 | 9.95E-08 | 3.62E-06 | BP |
| GO:0048839 | inner ear development | 56/2872 | 132/13036 | 1.93 | 1.12E-07 | 4.03E-06 | BP |
| GO:0050727 | regulation of inflammatory response | 94/2872 | 260/13036 | 1.64 | 1.13E-07 | 4.03E-06 | BP |
| GO:0060349 | bone morphogenesis | 39/2872 | 80/13036 | 2.21 | 1.17E-07 | 4.16E-06 | BP |
| GO:0042471 | ear morphogenesis | 40/2872 | 83/13036 | 2.19 | 1.21E-07 | 4.20E-06 | BP |
| GO:1990868 | response to chemokine | 29/2872 | 52/13036 | 2.53 | 1.21E-07 | 4.20E-06 | BP |
| GO:1990869 | cellular response to chemokine | 29/2872 | 52/13036 | 2.53 | 1.21E-07 | 4.20E-06 | BP |
| GO:0006694 | steroid biosynthetic process | 62/2872 | 152/13036 | 1.85 | 1.35E-07 | 4.62E-06 | BP |
| GO:0015850 | organic hydroxy compound transport | 68/2872 | 172/13036 | 1.79 | 1.43E-07 | 4.86E-06 | BP |
| GO:0019218 | regulation of steroid metabolic process | 46/2872 | 102/13036 | 2.05 | 1.73E-07 | 5.84E-06 | BP |
| GO:0050865 | regulation of cell activation | 135/2872 | 411/13036 | 1.49 | 1.85E-07 | 6.18E-06 | BP |
| GO:0007160 | cell-matrix adhesion | 73/2872 | 190/13036 | 1.74 | 1.95E-07 | 6.43E-06 | BP |
| GO:0050900 | leukocyte migration | 107/2872 | 309/13036 | 1.57 | 1.95E-07 | 6.43E-06 | BP |
| GO:0072073 | kidney epithelium development | 48/2872 | 109/13036 | 2 | 2.30E-07 | 7.51E-06 | BP |
| GO:0022600 | digestive system process | 30/2872 | 56/13036 | 2.43 | 2.45E-07 | 7.93E-06 | BP |
| GO:0003007 | heart morphogenesis | 73/2872 | 191/13036 | 1.73 | 2.48E-07 | 7.97E-06 | BP |
| GO:0048608 | reproductive structure development | 116/2872 | 343/13036 | 1.54 | 2.51E-07 | 8.02E-06 | BP |
| GO:0048565 | digestive tract development | 44/2872 | 97/13036 | 2.06 | 2.60E-07 | 8.24E-06 | BP |
| GO:0090257 | regulation of muscle system process | 65/2872 | 165/13036 | 1.79 | 3.12E-07 | 9.68E-06 | BP |
| GO:0032330 | regulation of chondrocyte differentiation | 28/2872 | 51/13036 | 2.49 | 3.12E-07 | 9.68E-06 | BP |
| GO:0048871 | multicellular organismal homeostasis | 126/2872 | 381/13036 | 1.5 | 3.13E-07 | 9.68E-06 | BP |
| GO:0019229 | regulation of vasoconstriction | 23/2872 | 38/13036 | 2.75 | 3.32E-07 | 1.02E-05 | BP |
| GO:0016126 | sterol biosynthetic process | 34/2872 | 68/13036 | 2.27 | 3.50E-07 | 1.07E-05 | BP |
| GO:1901342 | regulation of vasculature development | 98/2872 | 280/13036 | 1.59 | 3.60E-07 | 1.09E-05 | BP |
| GO:0007229 | integrin-mediated signaling pathway | 42/2872 | 92/13036 | 2.07 | 3.90E-07 | 1.17E-05 | BP |
| GO:0007189 | adenylate cyclase-activating G protein-coupled receptor signaling pathway | 38/2872 | 80/13036 | 2.16 | 3.97E-07 | 1.18E-05 | BP |
| GO:1901615 | organic hydroxy compound metabolic process | 135/2872 | 416/13036 | 1.47 | 4.01E-07 | 1.19E-05 | BP |
| GO:0007389 | pattern specification process | 106/2872 | 310/13036 | 1.55 | 4.50E-07 | 1.32E-05 | BP |
| GO:0061458 | reproductive system development | 116/2872 | 347/13036 | 1.52 | 4.95E-07 | 1.44E-05 | BP |
| GO:0006816 | calcium ion transport | 100/2872 | 289/13036 | 1.57 | 5.07E-07 | 1.47E-05 | BP |
| GO:0007188 | adenylate cyclase-modulating G protein-coupled receptor signaling pathway | 51/2872 | 121/13036 | 1.91 | 5.18E-07 | 1.49E-05 | BP |
| GO:0023061 | signal release | 127/2872 | 388/13036 | 1.49 | 5.27E-07 | 1.50E-05 | BP |
| GO:0050729 | positive regulation of inflammatory response | 44/2872 | 99/13036 | 2.02 | 5.29E-07 | 1.50E-05 | BP |
| GO:0070098 | chemokine-mediated signaling pathway | 25/2872 | 44/13036 | 2.58 | 5.59E-07 | 1.58E-05 | BP |
| GO:0003014 | renal system process | 40/2872 | 87/13036 | 2.09 | 5.83E-07 | 1.63E-05 | BP |
| GO:0007610 | behavior | 134/2872 | 415/13036 | 1.47 | 6.06E-07 | 1.69E-05 | BP |
| GO:0007596 | blood coagulation | 88/2872 | 248/13036 | 1.61 | 7.22E-07 | 2.00E-05 | BP |
| GO:0060047 | heart contraction | 69/2872 | 182/13036 | 1.72 | 7.44E-07 | 2.03E-05 | BP |
| GO:0002062 | chondrocyte differentiation | 44/2872 | 100/13036 | 2 | 7.44E-07 | 2.03E-05 | BP |
| GO:0048706 | embryonic skeletal system development | 43/2872 | 97/13036 | 2.01 | 7.73E-07 | 2.10E-05 | BP |
| GO:0007156 | homophilic cell adhesion via plasma membrane adhesion molecules | 49/2872 | 116/13036 | 1.92 | 7.95E-07 | 2.14E-05 | BP |
| GO:0007586 | digestion | 33/2872 | 67/13036 | 2.24 | 8.08E-07 | 2.15E-05 | BP |
| GO:0042472 | inner ear morphogenesis | 33/2872 | 67/13036 | 2.24 | 8.08E-07 | 2.15E-05 | BP |
| GO:0048638 | regulation of developmental growth | 94/2872 | 270/13036 | 1.58 | 8.12E-07 | 2.15E-05 | BP |
| GO:0007157 | heterophilic cell-cell adhesion via plasma membrane cell adhesion molecules | 18/2872 | 27/13036 | 3.03 | 8.33E-07 | 2.19E-05 | BP |
| GO:0050817 | coagulation | 88/2872 | 249/13036 | 1.6 | 8.78E-07 | 2.29E-05 | BP |
| GO:0003015 | heart process | 71/2872 | 190/13036 | 1.7 | 9.71E-07 | 2.52E-05 | BP |
| GO:0019216 | regulation of lipid metabolic process | 114/2872 | 344/13036 | 1.5 | 1.01E-06 | 2.61E-05 | BP |
| GO:0007565 | female pregnancy | 60/2872 | 153/13036 | 1.78 | 1.05E-06 | 2.69E-05 | BP |
| GO:0030500 | regulation of bone mineralization | 31/2872 | 62/13036 | 2.27 | 1.14E-06 | 2.91E-05 | BP |
| GO:0010463 | mesenchymal cell proliferation | 23/2872 | 40/13036 | 2.61 | 1.19E-06 | 3.02E-05 | BP |
| GO:0010466 | negative regulation of peptidase activity | 64/2872 | 167/13036 | 1.74 | 1.20E-06 | 3.02E-05 | BP |
| GO:0055123 | digestive system development | 45/2872 | 105/13036 | 1.95 | 1.37E-06 | 3.42E-05 | BP |
| GO:0044706 | multi-multicellular organism process | 65/2872 | 171/13036 | 1.73 | 1.39E-06 | 3.44E-05 | BP |
| GO:0006940 | regulation of smooth muscle contraction | 24/2872 | 43/13036 | 2.53 | 1.46E-06 | 3.61E-05 | BP |
| GO:0071772 | response to BMP | 52/2872 | 128/13036 | 1.84 | 1.56E-06 | 3.79E-05 | BP |
| GO:0071773 | cellular response to BMP stimulus | 52/2872 | 128/13036 | 1.84 | 1.56E-06 | 3.79E-05 | BP |
| GO:0007599 | hemostasis | 88/2872 | 252/13036 | 1.59 | 1.56E-06 | 3.79E-05 | BP |
| GO:0072511 | divalent inorganic cation transport | 110/2872 | 332/13036 | 1.5 | 1.58E-06 | 3.80E-05 | BP |
| GO:0030217 | T cell differentiation | 68/2872 | 182/13036 | 1.7 | 1.64E-06 | 3.94E-05 | BP |
| GO:0097530 | granulocyte migration | 41/2872 | 93/13036 | 2 | 1.65E-06 | 3.94E-05 | BP |
| GO:0050730 | regulation of peptidyl-tyrosine phosphorylation | 72/2872 | 196/13036 | 1.67 | 1.71E-06 | 4.05E-05 | BP |
| GO:0060537 | muscle tissue development | 103/2872 | 307/13036 | 1.52 | 1.77E-06 | 4.18E-05 | BP |
| GO:0072009 | nephron epithelium development | 37/2872 | 81/13036 | 2.07 | 1.86E-06 | 4.33E-05 | BP |
| GO:0019934 | cGMP-mediated signaling | 15/2872 | 21/13036 | 3.24 | 1.86E-06 | 4.33E-05 | BP |
| GO:0006066 | alcohol metabolic process | 102/2872 | 304/13036 | 1.52 | 1.98E-06 | 4.59E-05 | BP |
| GO:0003002 | regionalization | 84/2872 | 239/13036 | 1.6 | 2.00E-06 | 4.60E-05 | BP |
| GO:0014829 | vascular associated smooth muscle contraction | 14/2872 | 19/13036 | 3.34 | 2.30E-06 | 5.26E-05 | BP |
| GO:0050921 | positive regulation of chemotaxis | 46/2872 | 110/13036 | 1.9 | 2.39E-06 | 5.46E-05 | BP |
| GO:0048771 | tissue remodeling | 53/2872 | 133/13036 | 1.81 | 2.49E-06 | 5.64E-05 | BP |
| GO:0051384 | response to glucocorticoid | 45/2872 | 107/13036 | 1.91 | 2.55E-06 | 5.75E-05 | BP |
| GO:0010951 | negative regulation of endopeptidase activity | 61/2872 | 160/13036 | 1.73 | 2.58E-06 | 5.79E-05 | BP |
| GO:0031960 | response to corticosteroid | 49/2872 | 120/13036 | 1.85 | 2.60E-06 | 5.79E-05 | BP |
| GO:0071396 | cellular response to lipid | 142/2872 | 456/13036 | 1.41 | 2.76E-06 | 6.12E-05 | BP |
| GO:0008217 | regulation of blood pressure | 48/2872 | 117/13036 | 1.86 | 2.80E-06 | 6.12E-05 | BP |
| GO:0070167 | regulation of biomineral tissue development | 34/2872 | 73/13036 | 2.11 | 2.80E-06 | 6.12E-05 | BP |
| GO:0110149 | regulation of biomineralization | 34/2872 | 73/13036 | 2.11 | 2.80E-06 | 6.12E-05 | BP |
| GO:0070661 | leukocyte proliferation | 73/2872 | 202/13036 | 1.64 | 2.91E-06 | 6.33E-05 | BP |
| GO:0060562 | epithelial tube morphogenesis | 89/2872 | 259/13036 | 1.56 | 2.93E-06 | 6.34E-05 | BP |
| GO:0072087 | renal vesicle development | 12/2872 | 15/13036 | 3.63 | 2.97E-06 | 6.37E-05 | BP |
| GO:0002237 | response to molecule of bacterial origin | 87/2872 | 252/13036 | 1.57 | 3.05E-06 | 6.52E-05 | BP |
| GO:0042493 | response to drug | 101/2872 | 303/13036 | 1.51 | 3.09E-06 | 6.57E-05 | BP |
| GO:0007416 | synapse assembly | 53/2872 | 134/13036 | 1.8 | 3.24E-06 | 6.87E-05 | BP |
| GO:0010959 | regulation of metal ion transport | 91/2872 | 267/13036 | 1.55 | 3.36E-06 | 7.08E-05 | BP |
| GO:0048639 | positive regulation of developmental growth | 55/2872 | 141/13036 | 1.77 | 3.52E-06 | 7.35E-05 | BP |
| GO:0097529 | myeloid leukocyte migration | 55/2872 | 141/13036 | 1.77 | 3.52E-06 | 7.35E-05 | BP |
| GO:0002694 | regulation of leukocyte activation | 122/2872 | 382/13036 | 1.45 | 3.54E-06 | 7.35E-05 | BP |
| GO:0097755 | positive regulation of blood vessel diameter | 23/2872 | 42/13036 | 2.49 | 3.76E-06 | 7.77E-05 | BP |
| GO:0007411 | axon guidance | 78/2872 | 221/13036 | 1.6 | 3.82E-06 | 7.85E-05 | BP |
| GO:0030098 | lymphocyte differentiation | 88/2872 | 257/13036 | 1.55 | 3.92E-06 | 8.01E-05 | BP |
| GO:0034765 | regulation of ion transmembrane transport | 106/2872 | 323/13036 | 1.49 | 3.97E-06 | 8.07E-05 | BP |
| GO:0006695 | cholesterol biosynthetic process | 30/2872 | 62/13036 | 2.2 | 4.05E-06 | 8.14E-05 | BP |
| GO:1902653 | secondary alcohol biosynthetic process | 30/2872 | 62/13036 | 2.2 | 4.05E-06 | 8.14E-05 | BP |
| GO:0060333 | interferon-gamma-mediated signaling pathway | 34/2872 | 74/13036 | 2.09 | 4.10E-06 | 8.20E-05 | BP |
| GO:0061035 | regulation of cartilage development | 32/2872 | 68/13036 | 2.14 | 4.15E-06 | 8.20E-05 | BP |
| GO:0110110 | positive regulation of animal organ morphogenesis | 32/2872 | 68/13036 | 2.14 | 4.15E-06 | 8.20E-05 | BP |
| GO:0070838 | divalent metal ion transport | 107/2872 | 327/13036 | 1.49 | 4.16E-06 | 8.20E-05 | BP |
| GO:0002576 | platelet degranulation | 42/2872 | 99/13036 | 1.93 | 4.17E-06 | 8.20E-05 | BP |
| GO:0043406 | positive regulation of MAP kinase activity | 77/2872 | 218/13036 | 1.6 | 4.26E-06 | 8.33E-05 | BP |
| GO:0008016 | regulation of heart contraction | 60/2872 | 159/13036 | 1.71 | 4.57E-06 | 8.89E-05 | BP |
| GO:0014074 | response to purine-containing compound | 45/2872 | 109/13036 | 1.87 | 4.64E-06 | 8.92E-05 | BP |
| GO:0097485 | neuron projection guidance | 78/2872 | 222/13036 | 1.59 | 4.64E-06 | 8.92E-05 | BP |
| GO:0050927 | positive regulation of positive chemotaxis | 15/2872 | 22/13036 | 3.09 | 4.65E-06 | 8.92E-05 | BP |
| GO:0042110 | T cell activation | 110/2872 | 339/13036 | 1.47 | 4.73E-06 | 9.04E-05 | BP |
| GO:0045765 | regulation of angiogenesis | 86/2872 | 251/13036 | 1.56 | 4.89E-06 | 9.30E-05 | BP |
| GO:0007517 | muscle organ development | 101/2872 | 306/13036 | 1.5 | 5.03E-06 | 9.53E-05 | BP |
| GO:0090183 | regulation of kidney development | 22/2872 | 40/13036 | 2.5 | 5.56E-06 | 0.000104326 | BP |
| GO:0050810 | regulation of steroid biosynthetic process | 36/2872 | 81/13036 | 2.02 | 5.58E-06 | 0.000104326 | BP |
| GO:0040013 | negative regulation of locomotion | 88/2872 | 259/13036 | 1.54 | 5.58E-06 | 0.000104326 | BP |
| GO:0048661 | positive regulation of smooth muscle cell proliferation | 35/2872 | 78/13036 | 2.04 | 5.77E-06 | 0.000107243 | BP |
| GO:0006022 | aminoglycan metabolic process | 55/2872 | 143/13036 | 1.75 | 5.81E-06 | 0.000107496 | BP |
| GO:0007015 | actin filament organization | 120/2872 | 378/13036 | 1.44 | 5.88E-06 | 0.000108334 | BP |
| GO:0030879 | mammary gland development | 49/2872 | 123/13036 | 1.81 | 5.94E-06 | 0.000108979 | BP |
| GO:0043405 | regulation of MAP kinase activity | 95/2872 | 285/13036 | 1.51 | 6.01E-06 | 0.000109837 | BP |
| GO:0010810 | regulation of cell-substrate adhesion | 66/2872 | 181/13036 | 1.66 | 6.07E-06 | 0.000110021 | BP |
| GO:0001894 | tissue homeostasis | 68/2872 | 188/13036 | 1.64 | 6.07E-06 | 0.000110021 | BP |
| GO:0048732 | gland development | 113/2872 | 352/13036 | 1.46 | 6.17E-06 | 0.00011129 | BP |
| GO:0061041 | regulation of wound healing | 44/2872 | 107/13036 | 1.87 | 6.66E-06 | 0.000119586 | BP |
| GO:0001704 | formation of primary germ layer | 43/2872 | 104/13036 | 1.88 | 7.15E-06 | 0.000127902 | BP |
| GO:0046890 | regulation of lipid biosynthetic process | 60/2872 | 161/13036 | 1.69 | 7.23E-06 | 0.000128681 | BP |
| GO:1903530 | regulation of secretion by cell | 140/2872 | 456/13036 | 1.39 | 7.45E-06 | 0.000131954 | BP |
| GO:0003158 | endothelium development | 42/2872 | 101/13036 | 1.89 | 7.67E-06 | 0.000135337 | BP |
| GO:1902105 | regulation of leukocyte differentiation | 75/2872 | 214/13036 | 1.59 | 7.78E-06 | 0.000136769 | BP |
| GO:0051271 | negative regulation of cellular component movement | 88/2872 | 261/13036 | 1.53 | 7.89E-06 | 0.000138086 | BP |
| GO:0002009 | morphogenesis of an epithelium | 138/2872 | 449/13036 | 1.4 | 8.12E-06 | 0.000141571 | BP |
| GO:0071621 | granulocyte chemotaxis | 35/2872 | 79/13036 | 2.01 | 8.18E-06 | 0.0001419 | BP |
| GO:1901617 | organic hydroxy compound biosynthetic process | 69/2872 | 193/13036 | 1.62 | 8.24E-06 | 0.000142393 | BP |
| GO:0001837 | epithelial to mesenchymal transition | 51/2872 | 131/13036 | 1.77 | 8.39E-06 | 0.000144434 | BP |
| GO:0009952 | anterior/posterior pattern specification | 56/2872 | 148/13036 | 1.72 | 8.53E-06 | 0.000146201 | BP |
| GO:0046649 | lymphocyte activation | 149/2872 | 492/13036 | 1.37 | 8.69E-06 | 0.0001483 | BP |
| GO:0006937 | regulation of muscle contraction | 44/2872 | 108/13036 | 1.85 | 8.87E-06 | 0.000150719 | BP |
| GO:0007422 | peripheral nervous system development | 30/2872 | 64/13036 | 2.13 | 9.09E-06 | 0.000153802 | BP |
| GO:0003206 | cardiac chamber morphogenesis | 39/2872 | 92/13036 | 1.92 | 9.30E-06 | 0.000156771 | BP |
| GO:0050673 | epithelial cell proliferation | 105/2872 | 325/13036 | 1.47 | 9.56E-06 | 0.00016051 | BP |
| GO:0071560 | cellular response to transforming growth factor beta stimulus | 71/2872 | 201/13036 | 1.6 | 9.89E-06 | 0.000165462 | BP |
| GO:1901654 | response to ketone | 61/2872 | 166/13036 | 1.67 | 1.02E-05 | 0.000169542 | BP |
| GO:0061572 | actin filament bundle organization | 54/2872 | 142/13036 | 1.73 | 1.04E-05 | 0.000171795 | BP |
| GO:0050926 | regulation of positive chemotaxis | 15/2872 | 23/13036 | 2.96 | 1.06E-05 | 0.000175011 | BP |
| GO:0050974 | detection of mechanical stimulus involved in sensory perception | 15/2872 | 23/13036 | 2.96 | 1.06E-05 | 0.000175011 | BP |
| GO:0046165 | alcohol biosynthetic process | 51/2872 | 132/13036 | 1.75 | 1.08E-05 | 0.000175878 | BP |
| GO:0030856 | regulation of epithelial cell differentiation | 45/2872 | 112/13036 | 1.82 | 1.08E-05 | 0.000175878 | BP |
| GO:0032496 | response to lipopolysaccharide | 82/2872 | 241/13036 | 1.54 | 1.08E-05 | 0.000175878 | BP |
| GO:0072077 | renal vesicle morphogenesis | 11/2872 | 14/13036 | 3.57 | 1.09E-05 | 0.000175878 | BP |
| GO:0051146 | striated muscle cell differentiation | 77/2872 | 223/13036 | 1.57 | 1.10E-05 | 0.000176711 | BP |
| GO:0022407 | regulation of cell-cell adhesion | 101/2872 | 311/13036 | 1.47 | 1.10E-05 | 0.000176711 | BP |
| GO:0048333 | mesodermal cell differentiation | 17/2872 | 28/13036 | 2.76 | 1.11E-05 | 0.000177142 | BP |
| GO:0048738 | cardiac muscle tissue development | 60/2872 | 163/13036 | 1.67 | 1.13E-05 | 0.000179956 | BP |
| GO:0014706 | striated muscle tissue development | 95/2872 | 289/13036 | 1.49 | 1.15E-05 | 0.000182392 | BP |
| GO:0052548 | regulation of endopeptidase activity | 99/2872 | 304/13036 | 1.48 | 1.18E-05 | 0.000186286 | BP |
| GO:0034762 | regulation of transmembrane transport | 122/2872 | 391/13036 | 1.42 | 1.24E-05 | 0.000195178 | BP |
| GO:1990266 | neutrophil migration | 33/2872 | 74/13036 | 2.02 | 1.24E-05 | 0.000195178 | BP |
| GO:2000146 | negative regulation of cell motility | 79/2872 | 231/13036 | 1.55 | 1.26E-05 | 0.000197819 | BP |
| GO:0022612 | gland morphogenesis | 43/2872 | 106/13036 | 1.84 | 1.27E-05 | 0.000197892 | BP |
| GO:0015837 | amine transport | 26/2872 | 53/13036 | 2.23 | 1.29E-05 | 0.000199954 | BP |
| GO:0090130 | tissue migration | 88/2872 | 264/13036 | 1.51 | 1.31E-05 | 0.000202184 | BP |
| GO:1900046 | regulation of hemostasis | 28/2872 | 59/13036 | 2.15 | 1.34E-05 | 0.00020682 | BP |
| GO:0007369 | gastrulation | 56/2872 | 150/13036 | 1.69 | 1.35E-05 | 0.000208117 | BP |
| GO:0007178 | transmembrane receptor protein serine/threonine kinase signaling pathway | 92/2872 | 279/13036 | 1.5 | 1.37E-05 | 0.000208935 | BP |
| GO:0061564 | axon development | 130/2872 | 423/13036 | 1.39 | 1.48E-05 | 0.000226134 | BP |
| GO:0046425 | regulation of receptor signaling pathway via JAK-STAT | 36/2872 | 84/13036 | 1.95 | 1.51E-05 | 0.000230104 | BP |
| GO:1901888 | regulation of cell junction assembly | 59/2872 | 161/13036 | 1.66 | 1.55E-05 | 0.000235257 | BP |
| GO:0051924 | regulation of calcium ion transport | 63/2872 | 175/13036 | 1.63 | 1.56E-05 | 0.000235257 | BP |
| GO:0071559 | response to transforming growth factor beta | 72/2872 | 207/13036 | 1.58 | 1.58E-05 | 0.000236849 | BP |
| GO:0010817 | regulation of hormone levels | 112/2872 | 355/13036 | 1.43 | 1.62E-05 | 0.000242852 | BP |
| GO:0045580 | regulation of T cell differentiation | 43/2872 | 107/13036 | 1.82 | 1.67E-05 | 0.000249807 | BP |
| GO:0045216 | cell-cell junction organization | 60/2872 | 165/13036 | 1.65 | 1.74E-05 | 0.000258054 | BP |
| GO:0050982 | detection of mechanical stimulus | 19/2872 | 34/13036 | 2.54 | 1.79E-05 | 0.000265288 | BP |
| GO:0050918 | positive chemotaxis | 24/2872 | 48/13036 | 2.27 | 1.84E-05 | 0.000269596 | BP |
| GO:0060688 | regulation of morphogenesis of a branching structure | 24/2872 | 48/13036 | 2.27 | 1.84E-05 | 0.000269596 | BP |
| GO:0071900 | regulation of protein serine/threonine kinase activity | 135/2872 | 444/13036 | 1.38 | 1.84E-05 | 0.000269596 | BP |
| GO:0050678 | regulation of epithelial cell proliferation | 92/2872 | 281/13036 | 1.49 | 1.87E-05 | 0.000273541 | BP |
| GO:0048015 | phosphatidylinositol-mediated signaling | 57/2872 | 155/13036 | 1.67 | 1.91E-05 | 0.000273792 | BP |
| GO:0042698 | ovulation cycle | 25/2872 | 51/13036 | 2.22 | 1.91E-05 | 0.000273792 | BP |
| GO:0051952 | regulation of amine transport | 25/2872 | 51/13036 | 2.22 | 1.91E-05 | 0.000273792 | BP |
| GO:0015711 | organic anion transport | 113/2872 | 360/13036 | 1.42 | 1.92E-05 | 0.000273792 | BP |
| GO:0001819 | positive regulation of cytokine production | 104/2872 | 326/13036 | 1.45 | 1.92E-05 | 0.000273792 | BP |
| GO:0001505 | regulation of neurotransmitter levels | 61/2872 | 169/13036 | 1.64 | 1.93E-05 | 0.000273792 | BP |
| GO:1903034 | regulation of response to wounding | 50/2872 | 131/13036 | 1.73 | 1.93E-05 | 0.000273792 | BP |
| GO:0001656 | metanephros development | 30/2872 | 66/13036 | 2.06 | 1.93E-05 | 0.000273792 | BP |
| GO:1902930 | regulation of alcohol biosynthetic process | 30/2872 | 66/13036 | 2.06 | 1.93E-05 | 0.000273792 | BP |
| GO:1904892 | regulation of receptor signaling pathway via STAT | 37/2872 | 88/13036 | 1.91 | 1.95E-05 | 0.000275058 | BP |
| GO:0046427 | positive regulation of receptor signaling pathway via JAK-STAT | 27/2872 | 57/13036 | 2.15 | 1.99E-05 | 0.000278825 | BP |
| GO:1904894 | positive regulation of receptor signaling pathway via STAT | 28/2872 | 60/13036 | 2.12 | 1.99E-05 | 0.000278825 | BP |
| GO:0006941 | striated muscle contraction | 44/2872 | 111/13036 | 1.8 | 2.02E-05 | 0.000281609 | BP |
| GO:0015849 | organic acid transport | 81/2872 | 241/13036 | 1.53 | 2.03E-05 | 0.000282701 | BP |
| GO:0050920 | regulation of chemotaxis | 62/2872 | 173/13036 | 1.63 | 2.13E-05 | 0.000295164 | BP |
| GO:0006836 | neurotransmitter transport | 58/2872 | 159/13036 | 1.66 | 2.14E-05 | 0.000295164 | BP |
| GO:0010720 | positive regulation of cell development | 137/2872 | 453/13036 | 1.37 | 2.14E-05 | 0.000295164 | BP |
| GO:0030203 | glycosaminoglycan metabolic process | 51/2872 | 135/13036 | 1.71 | 2.22E-05 | 0.000305547 | BP |
| GO:0046942 | carboxylic acid transport | 80/2872 | 238/13036 | 1.53 | 2.27E-05 | 0.000310617 | BP |
| GO:0042692 | muscle cell differentiation | 97/2872 | 301/13036 | 1.46 | 2.31E-05 | 0.000315247 | BP |
| GO:0001667 | ameboidal-type cell migration | 111/2872 | 354/13036 | 1.42 | 2.38E-05 | 0.000323689 | BP |
| GO:0030509 | BMP signaling pathway | 47/2872 | 122/13036 | 1.75 | 2.57E-05 | 0.000347231 | BP |
| GO:0090189 | regulation of branching involved in ureteric bud morphogenesis | 12/2872 | 17/13036 | 3.2 | 2.57E-05 | 0.000347231 | BP |
| GO:0007259 | receptor signaling pathway via JAK-STAT | 41/2872 | 102/13036 | 1.82 | 2.59E-05 | 0.000347865 | BP |
| GO:0048736 | appendage development | 54/2872 | 146/13036 | 1.68 | 2.60E-05 | 0.000347865 | BP |
| GO:0060173 | limb development | 54/2872 | 146/13036 | 1.68 | 2.60E-05 | 0.000347865 | BP |
| GO:0045834 | positive regulation of lipid metabolic process | 44/2872 | 112/13036 | 1.78 | 2.62E-05 | 0.000349533 | BP |
| GO:0050808 | synapse organization | 106/2872 | 336/13036 | 1.43 | 2.72E-05 | 0.000361099 | BP |
| GO:0046683 | response to organophosphorus | 40/2872 | 99/13036 | 1.83 | 2.81E-05 | 0.000372167 | BP |
| GO:0030593 | neutrophil chemotaxis | 28/2872 | 61/13036 | 2.08 | 2.91E-05 | 0.000383232 | BP |
| GO:0050818 | regulation of coagulation | 28/2872 | 61/13036 | 2.08 | 2.91E-05 | 0.000383232 | BP |
| GO:0030193 | regulation of blood coagulation | 27/2872 | 58/13036 | 2.11 | 2.95E-05 | 0.000386082 | BP |
| GO:0090181 | regulation of cholesterol metabolic process | 26/2872 | 55/13036 | 2.15 | 2.95E-05 | 0.000386082 | BP |
| GO:0048667 | cell morphogenesis involved in neuron differentiation | 145/2872 | 487/13036 | 1.35 | 2.99E-05 | 0.000389484 | BP |
| GO:0032102 | negative regulation of response to external stimulus | 89/2872 | 273/13036 | 1.48 | 3.05E-05 | 0.000396561 | BP |
| GO:0071902 | positive regulation of protein serine/threonine kinase activity | 93/2872 | 288/13036 | 1.47 | 3.09E-05 | 0.000399827 | BP |
| GO:0035137 | hindlimb morphogenesis | 16/2872 | 27/13036 | 2.69 | 3.09E-05 | 0.000399827 | BP |
| GO:0007162 | negative regulation of cell adhesion | 74/2872 | 218/13036 | 1.54 | 3.13E-05 | 0.000402589 | BP |
| GO:0030336 | negative regulation of cell migration | 75/2872 | 222/13036 | 1.53 | 3.33E-05 | 0.000427373 | BP |
| GO:0043114 | regulation of vascular permeability | 20/2872 | 38/13036 | 2.39 | 3.51E-05 | 0.000449313 | BP |
| GO:0045778 | positive regulation of ossification | 32/2872 | 74/13036 | 1.96 | 3.57E-05 | 0.000456095 | BP |
| GO:0007409 | axonogenesis | 118/2872 | 384/13036 | 1.39 | 3.65E-05 | 0.000464594 | BP |
| GO:0090287 | regulation of cellular response to growth factor stimulus | 77/2872 | 230/13036 | 1.52 | 3.75E-05 | 0.000474577 | BP |
| GO:0070542 | response to fatty acid | 31/2872 | 71/13036 | 1.98 | 3.75E-05 | 0.000474577 | BP |
| GO:0051346 | negative regulation of hydrolase activity | 103/2872 | 327/13036 | 1.43 | 3.77E-05 | 0.0004749 | BP |
| GO:0052547 | regulation of peptidase activity | 104/2872 | 331/13036 | 1.43 | 3.88E-05 | 0.000487265 | BP |
| GO:0071496 | cellular response to external stimulus | 92/2872 | 286/13036 | 1.46 | 4.01E-05 | 0.000502406 | BP |
| GO:0097696 | receptor signaling pathway via STAT | 42/2872 | 107/13036 | 1.78 | 4.06E-05 | 0.000506586 | BP |
| GO:0061098 | positive regulation of protein tyrosine kinase activity | 22/2872 | 44/13036 | 2.27 | 4.08E-05 | 0.000507686 | BP |
| GO:0072283 | metanephric renal vesicle morphogenesis | 9/2872 | 11/13036 | 3.71 | 4.29E-05 | 0.00053251 | BP |
| GO:0048844 | artery morphogenesis | 25/2872 | 53/13036 | 2.14 | 4.38E-05 | 0.000542143 | BP |
| GO:0048017 | inositol lipid-mediated signaling | 57/2872 | 159/13036 | 1.63 | 4.45E-05 | 0.000547752 | BP |
| GO:0032332 | positive regulation of chondrocyte differentiation | 13/2872 | 20/13036 | 2.95 | 4.46E-05 | 0.000547752 | BP |
| GO:0051482 | positive regulation of cytosolic calcium ion concentration involved in phospholipase C-activating G protein-coupled signaling pathway | 13/2872 | 20/13036 | 2.95 | 4.46E-05 | 0.000547752 | BP |
| GO:0055078 | sodium ion homeostasis | 15/2872 | 25/13036 | 2.72 | 4.49E-05 | 0.000549738 | BP |
| GO:0060840 | artery development | 33/2872 | 78/13036 | 1.92 | 4.64E-05 | 0.000566256 | BP |
| GO:0051047 | positive regulation of secretion | 79/2872 | 239/13036 | 1.5 | 4.92E-05 | 0.000597868 | BP |
| GO:0007548 | sex differentiation | 69/2872 | 203/13036 | 1.54 | 5.44E-05 | 0.000659138 | BP |
| GO:2000027 | regulation of animal organ morphogenesis | 72/2872 | 214/13036 | 1.53 | 5.51E-05 | 0.000666144 | BP |
| GO:0006959 | humoral immune response | 44/2872 | 115/13036 | 1.74 | 5.57E-05 | 0.000669111 | BP |
| GO:0035051 | cardiocyte differentiation | 44/2872 | 115/13036 | 1.74 | 5.57E-05 | 0.000669111 | BP |
| GO:0060560 | developmental growth involved in morphogenesis | 67/2872 | 196/13036 | 1.55 | 5.68E-05 | 0.000680866 | BP |
| GO:0035725 | sodium ion transmembrane transport | 41/2872 | 105/13036 | 1.77 | 5.74E-05 | 0.0006828 | BP |
| GO:0050433 | regulation of catecholamine secretion | 16/2872 | 28/13036 | 2.59 | 5.75E-05 | 0.0006828 | BP |
| GO:0090184 | positive regulation of kidney development | 16/2872 | 28/13036 | 2.59 | 5.75E-05 | 0.0006828 | BP |
| GO:0035107 | appendage morphogenesis | 46/2872 | 122/13036 | 1.71 | 5.82E-05 | 0.000687134 | BP |
| GO:0035108 | limb morphogenesis | 46/2872 | 122/13036 | 1.71 | 5.82E-05 | 0.000687134 | BP |
| GO:0050679 | positive regulation of epithelial cell proliferation | 56/2872 | 157/13036 | 1.62 | 6.07E-05 | 0.000714543 | BP |
| GO:2000649 | regulation of sodium ion transmembrane transporter activity | 21/2872 | 42/13036 | 2.27 | 6.08E-05 | 0.000714543 | BP |
| GO:0061217 | regulation of mesonephros development | 12/2872 | 18/13036 | 3.03 | 6.16E-05 | 0.000718193 | BP |
| GO:0097205 | renal filtration | 12/2872 | 18/13036 | 3.03 | 6.16E-05 | 0.000718193 | BP |
| GO:0010631 | epithelial cell migration | 84/2872 | 259/13036 | 1.47 | 6.18E-05 | 0.000718193 | BP |
| GO:0090132 | epithelium migration | 84/2872 | 259/13036 | 1.47 | 6.18E-05 | 0.000718193 | BP |
| GO:0007498 | mesoderm development | 37/2872 | 92/13036 | 1.83 | 6.22E-05 | 0.000720582 | BP |
| GO:0015844 | monoamine transport | 22/2872 | 45/13036 | 2.22 | 6.33E-05 | 0.000730737 | BP |
| GO:0060415 | muscle tissue morphogenesis | 23/2872 | 48/13036 | 2.17 | 6.46E-05 | 0.000738672 | BP |
| GO:0032331 | negative regulation of chondrocyte differentiation | 14/2872 | 23/13036 | 2.76 | 6.50E-05 | 0.000738672 | BP |
| GO:0042311 | vasodilation | 14/2872 | 23/13036 | 2.76 | 6.50E-05 | 0.000738672 | BP |
| GO:0098801 | regulation of renal system process | 14/2872 | 23/13036 | 2.76 | 6.50E-05 | 0.000738672 | BP |
| GO:1900120 | regulation of receptor binding | 14/2872 | 23/13036 | 2.76 | 6.50E-05 | 0.000738672 | BP |
| GO:0042100 | B cell proliferation | 24/2872 | 51/13036 | 2.14 | 6.50E-05 | 0.000738672 | BP |
| GO:0055002 | striated muscle cell development | 47/2872 | 126/13036 | 1.69 | 6.63E-05 | 0.000751085 | BP |
| GO:0006820 | anion transport | 135/2872 | 455/13036 | 1.35 | 6.66E-05 | 0.000751639 | BP |
| GO:0006865 | amino acid transport | 42/2872 | 109/13036 | 1.75 | 6.73E-05 | 0.000751639 | BP |
| GO:0060337 | type I interferon signaling pathway | 32/2872 | 76/13036 | 1.91 | 6.73E-05 | 0.000751639 | BP |
| GO:0071357 | cellular response to type I interferon | 32/2872 | 76/13036 | 1.91 | 6.73E-05 | 0.000751639 | BP |
| GO:0060048 | cardiac muscle contraction | 36/2872 | 89/13036 | 1.84 | 6.74E-05 | 0.000751639 | BP |
| GO:1904018 | positive regulation of vasculature development | 55/2872 | 154/13036 | 1.62 | 6.76E-05 | 0.000751639 | BP |
| GO:0045619 | regulation of lymphocyte differentiation | 49/2872 | 133/13036 | 1.67 | 6.76E-05 | 0.000751639 | BP |
| GO:0045429 | positive regulation of nitric oxide biosynthetic process | 17/2872 | 31/13036 | 2.49 | 6.86E-05 | 0.00075921 | BP |
| GO:0048247 | lymphocyte chemotaxis | 17/2872 | 31/13036 | 2.49 | 6.86E-05 | 0.00075921 | BP |
| GO:0045860 | positive regulation of protein kinase activity | 131/2872 | 440/13036 | 1.35 | 7.16E-05 | 0.000788383 | BP |
| GO:1901890 | positive regulation of cell junction assembly | 31/2872 | 73/13036 | 1.93 | 7.17E-05 | 0.000788383 | BP |
| GO:0003205 | cardiac chamber development | 48/2872 | 130/13036 | 1.68 | 7.49E-05 | 0.000822148 | BP |
| GO:0030168 | platelet activation | 45/2872 | 120/13036 | 1.7 | 8.10E-05 | 0.000886875 | BP |
| GO:0033687 | osteoblast proliferation | 15/2872 | 26/13036 | 2.62 | 8.46E-05 | 0.000923311 | BP |
| GO:0010876 | lipid localization | 98/2872 | 314/13036 | 1.42 | 8.51E-05 | 0.000926783 | BP |
| GO:0032943 | mononuclear cell proliferation | 63/2872 | 184/13036 | 1.55 | 8.84E-05 | 0.000959602 | BP |
| GO:0042445 | hormone metabolic process | 52/2872 | 145/13036 | 1.63 | 9.32E-05 | 0.001003086 | BP |
| GO:0021602 | cranial nerve morphogenesis | 13/2872 | 21/13036 | 2.81 | 9.35E-05 | 0.001003086 | BP |
| GO:0033688 | regulation of osteoblast proliferation | 13/2872 | 21/13036 | 2.81 | 9.35E-05 | 0.001003086 | BP |
| GO:0045932 | negative regulation of muscle contraction | 13/2872 | 21/13036 | 2.81 | 9.35E-05 | 0.001003086 | BP |
| GO:0051017 | actin filament bundle assembly | 50/2872 | 138/13036 | 1.64 | 9.37E-05 | 0.001003086 | BP |
| GO:0051145 | smooth muscle cell differentiation | 25/2872 | 55/13036 | 2.06 | 9.38E-05 | 0.001003086 | BP |
| GO:0030858 | positive regulation of epithelial cell differentiation | 21/2872 | 43/13036 | 2.22 | 9.43E-05 | 0.001005776 | BP |
| GO:0010718 | positive regulation of epithelial to mesenchymal transition | 22/2872 | 46/13036 | 2.17 | 9.62E-05 | 0.00102147 | BP |
| GO:0045446 | endothelial cell differentiation | 35/2872 | 87/13036 | 1.83 | 9.65E-05 | 0.00102147 | BP |
| GO:0086003 | cardiac muscle cell contraction | 23/2872 | 49/13036 | 2.13 | 9.65E-05 | 0.00102147 | BP |
| GO:0061037 | negative regulation of cartilage development | 16/2872 | 29/13036 | 2.5 | 0.000102022 | 0.001076648 | BP |
| GO:0045766 | positive regulation of angiogenesis | 49/2872 | 135/13036 | 1.65 | 0.000104139 | 0.001096199 | BP |
| GO:0018108 | peptidyl-tyrosine phosphorylation | 91/2872 | 289/13036 | 1.43 | 0.000105628 | 0.001109058 | BP |
| GO:0045986 | negative regulation of smooth muscle contraction | 10/2872 | 14/13036 | 3.24 | 0.000109593 | 0.001147787 | BP |
| GO:0006641 | triglyceride metabolic process | 29/2872 | 68/13036 | 1.94 | 0.000110677 | 0.001156222 | BP |
| GO:0010811 | positive regulation of cell-substrate adhesion | 39/2872 | 101/13036 | 1.75 | 0.000114336 | 0.001191442 | BP |
| GO:0055001 | muscle cell development | 50/2872 | 139/13036 | 1.63 | 0.000115455 | 0.001200091 | BP |
| GO:1904407 | positive regulation of nitric oxide metabolic process | 17/2872 | 32/13036 | 2.41 | 0.000116491 | 0.001207832 | BP |
| GO:0046889 | positive regulation of lipid biosynthetic process | 28/2872 | 65/13036 | 1.96 | 0.000117139 | 0.001211521 | BP |
| GO:0001558 | regulation of cell growth | 107/2872 | 351/13036 | 1.38 | 0.000117609 | 0.001211812 | BP |
| GO:0070371 | ERK1 and ERK2 cascade | 74/2872 | 226/13036 | 1.49 | 0.000117752 | 0.001211812 | BP |
| GO:0048332 | mesoderm morphogenesis | 27/2872 | 62/13036 | 1.98 | 0.000123359 | 0.001266377 | BP |
| GO:0045927 | positive regulation of growth | 69/2872 | 208/13036 | 1.51 | 0.000126906 | 0.001299571 | BP |
| GO:1900047 | negative regulation of hemostasis | 18/2872 | 35/13036 | 2.33 | 0.00012772 | 0.001304684 | BP |
| GO:0030326 | embryonic limb morphogenesis | 40/2872 | 105/13036 | 1.73 | 0.000132762 | 0.001349538 | BP |
| GO:0035113 | embryonic appendage morphogenesis | 40/2872 | 105/13036 | 1.73 | 0.000132762 | 0.001349538 | BP |
| GO:1905153 | regulation of membrane invagination | 9/2872 | 12/13036 | 3.4 | 0.000137776 | 0.001397088 | BP |
| GO:0048644 | muscle organ morphogenesis | 24/2872 | 53/13036 | 2.06 | 0.000138652 | 0.001402537 | BP |
| GO:0016266 | O-glycan processing | 20/2872 | 41/13036 | 2.21 | 0.00014081 | 0.001420906 | BP |
| GO:0086001 | cardiac muscle cell action potential | 23/2872 | 50/13036 | 2.09 | 0.000141797 | 0.001427391 | BP |
| GO:1903708 | positive regulation of hemopoiesis | 57/2872 | 165/13036 | 1.57 | 0.000142935 | 0.001435056 | BP |
| GO:0072132 | mesenchyme morphogenesis | 21/2872 | 44/13036 | 2.17 | 0.000143251 | 0.001435056 | BP |
| GO:0051588 | regulation of neurotransmitter transport | 36/2872 | 92/13036 | 1.78 | 0.000150822 | 0.001507268 | BP |
| GO:0009913 | epidermal cell differentiation | 53/2872 | 151/13036 | 1.59 | 0.000152034 | 0.00151572 | BP |
| GO:0014065 | phosphatidylinositol 3-kinase signaling | 45/2872 | 123/13036 | 1.66 | 0.000158587 | 0.001572206 | BP |
| GO:0048545 | response to steroid hormone | 86/2872 | 273/13036 | 1.43 | 0.000158642 | 0.001572206 | BP |
| GO:0018212 | peptidyl-tyrosine modification | 91/2872 | 292/13036 | 1.41 | 0.000158837 | 0.001572206 | BP |
| GO:0060601 | lateral sprouting from an epithelium | 8/2872 | 10/13036 | 3.63 | 0.00016058 | 0.001585682 | BP |
| GO:0001508 | action potential | 35/2872 | 89/13036 | 1.78 | 0.000165024 | 0.001621834 | BP |
| GO:0009581 | detection of external stimulus | 35/2872 | 89/13036 | 1.78 | 0.000165024 | 0.001621834 | BP |
| GO:0010721 | negative regulation of cell development | 83/2872 | 262/13036 | 1.44 | 0.000166438 | 0.00163187 | BP |
| GO:0042632 | cholesterol homeostasis | 27/2872 | 63/13036 | 1.95 | 0.000170995 | 0.001672592 | BP |
| GO:0007600 | sensory perception | 101/2872 | 331/13036 | 1.39 | 0.000173336 | 0.001690657 | BP |
| GO:0050432 | catecholamine secretion | 16/2872 | 30/13036 | 2.42 | 0.000174065 | 0.001690657 | BP |
| GO:0061036 | positive regulation of cartilage development | 16/2872 | 30/13036 | 2.42 | 0.000174065 | 0.001690657 | BP |
| GO:0046632 | alpha-beta T cell differentiation | 31/2872 | 76/13036 | 1.85 | 0.000175832 | 0.001703668 | BP |
| GO:0030900 | forebrain development | 90/2872 | 289/13036 | 1.41 | 0.000176983 | 0.001703668 | BP |
| GO:0048880 | sensory system development | 90/2872 | 289/13036 | 1.41 | 0.000176983 | 0.001703668 | BP |
| GO:0006024 | glycosaminoglycan biosynthetic process | 37/2872 | 96/13036 | 1.75 | 0.000177047 | 0.001703668 | BP |
| GO:1903532 | positive regulation of secretion by cell | 73/2872 | 225/13036 | 1.47 | 0.000179365 | 0.001721974 | BP |
| GO:0001707 | mesoderm formation | 26/2872 | 60/13036 | 1.97 | 0.000180606 | 0.00172988 | BP |
| GO:0002053 | positive regulation of mesenchymal cell proliferation | 13/2872 | 22/13036 | 2.68 | 0.000182523 | 0.001740207 | BP |
| GO:0045940 | positive regulation of steroid metabolic process | 13/2872 | 22/13036 | 2.68 | 0.000182523 | 0.001740207 | BP |
| GO:0051962 | positive regulation of nervous system development | 127/2872 | 433/13036 | 1.33 | 0.000186526 | 0.001774297 | BP |
| GO:0003094 | glomerular filtration | 11/2872 | 17/13036 | 2.94 | 0.000189049 | 0.001794175 | BP |
| GO:0048704 | embryonic skeletal system morphogenesis | 30/2872 | 73/13036 | 1.87 | 0.000189794 | 0.00179714 | BP |
| GO:0030501 | positive regulation of bone mineralization | 17/2872 | 33/13036 | 2.34 | 0.000191172 | 0.001801958 | BP |
| GO:1902895 | positive regulation of pri-miRNA transcription by RNA polymerase II | 17/2872 | 33/13036 | 2.34 | 0.000191172 | 0.001801958 | BP |
| GO:0009991 | response to extracellular stimulus | 117/2872 | 394/13036 | 1.35 | 0.000191807 | 0.001803838 | BP |
| GO:0035637 | multicellular organismal signaling | 45/2872 | 124/13036 | 1.65 | 0.000196528 | 0.001844057 | BP |
| GO:0048546 | digestive tract morphogenesis | 18/2872 | 36/13036 | 2.27 | 0.000203124 | 0.001897362 | BP |
| GO:0050819 | negative regulation of coagulation | 18/2872 | 36/13036 | 2.27 | 0.000203124 | 0.001897362 | BP |
| GO:0014068 | positive regulation of phosphatidylinositol 3-kinase signaling | 29/2872 | 70/13036 | 1.88 | 0.000204288 | 0.001903855 | BP |
| GO:0030199 | collagen fibril organization | 23/2872 | 51/13036 | 2.05 | 0.000204873 | 0.001903855 | BP |
| GO:0006023 | aminoglycan biosynthetic process | 38/2872 | 100/13036 | 1.72 | 0.000205196 | 0.001903855 | BP |
| GO:0035265 | organ growth | 50/2872 | 142/13036 | 1.6 | 0.000211025 | 0.001953564 | BP |
| GO:0150076 | neuroinflammatory response | 21/2872 | 45/13036 | 2.12 | 0.000213248 | 0.00196787 | BP |
| GO:0046660 | female sex differentiation | 35/2872 | 90/13036 | 1.77 | 0.000213519 | 0.00196787 | BP |
| GO:0034340 | response to type I interferon | 32/2872 | 80/13036 | 1.82 | 0.000214263 | 0.001970347 | BP |
| GO:0046651 | lymphocyte proliferation | 61/2872 | 182/13036 | 1.52 | 0.000221778 | 0.002034948 | BP |
| GO:0045926 | negative regulation of growth | 68/2872 | 208/13036 | 1.48 | 0.000229327 | 0.00209825 | BP |
| GO:0002573 | myeloid leukocyte differentiation | 56/2872 | 164/13036 | 1.55 | 0.000229689 | 0.00209825 | BP |
| GO:0035270 | endocrine system development | 31/2872 | 77/13036 | 1.83 | 0.000232926 | 0.002117208 | BP |
| GO:0070252 | actin-mediated cell contraction | 31/2872 | 77/13036 | 1.83 | 0.000232926 | 0.002117208 | BP |
| GO:0022617 | extracellular matrix disassembly | 27/2872 | 64/13036 | 1.91 | 0.000234317 | 0.002117208 | BP |
| GO:0055006 | cardiac cell development | 27/2872 | 64/13036 | 1.91 | 0.000234317 | 0.002117208 | BP |
| GO:0055092 | sterol homeostasis | 27/2872 | 64/13036 | 1.91 | 0.000234317 | 0.002117208 | BP |
| GO:0002685 | regulation of leukocyte migration | 54/2872 | 157/13036 | 1.56 | 0.00023934 | 0.002157806 | BP |
| GO:0007584 | response to nutrient | 47/2872 | 132/13036 | 1.62 | 0.000239851 | 0.002157806 | BP |
| GO:0043270 | positive regulation of ion transport | 63/2872 | 190/13036 | 1.51 | 0.000247546 | 0.002222222 | BP |
| GO:0051963 | regulation of synapse assembly | 30/2872 | 74/13036 | 1.84 | 0.000252679 | 0.002263399 | BP |
| GO:0150063 | visual system development | 89/2872 | 288/13036 | 1.4 | 0.000256549 | 0.002293114 | BP |
| GO:0021515 | cell differentiation in spinal cord | 15/2872 | 28/13036 | 2.43 | 0.000260361 | 0.002317194 | BP |
| GO:0048483 | autonomic nervous system development | 15/2872 | 28/13036 | 2.43 | 0.000260361 | 0.002317194 | BP |
| GO:0010934 | macrophage cytokine production | 10/2872 | 15/13036 | 3.03 | 0.000263666 | 0.00232664 | BP |
| GO:0030878 | thyroid gland development | 10/2872 | 15/13036 | 3.03 | 0.000263666 | 0.00232664 | BP |
| GO:0044062 | regulation of excretion | 10/2872 | 15/13036 | 3.03 | 0.000263666 | 0.00232664 | BP |
| GO:0060602 | branch elongation of an epithelium | 10/2872 | 15/13036 | 3.03 | 0.000263666 | 0.00232664 | BP |
| GO:0030431 | sleep | 12/2872 | 20/13036 | 2.72 | 0.000267101 | 0.002346963 | BP |
| GO:0044058 | regulation of digestive system process | 12/2872 | 20/13036 | 2.72 | 0.000267101 | 0.002346963 | BP |
| GO:0006869 | lipid transport | 86/2872 | 277/13036 | 1.41 | 0.000272684 | 0.002390644 | BP |
| GO:0071260 | cellular response to mechanical stimulus | 29/2872 | 71/13036 | 1.85 | 0.000273455 | 0.002390644 | BP |
| GO:0006638 | neutral lipid metabolic process | 35/2872 | 91/13036 | 1.75 | 0.000274378 | 0.002390644 | BP |
| GO:0006639 | acylglycerol metabolic process | 35/2872 | 91/13036 | 1.75 | 0.000274378 | 0.002390644 | BP |
| GO:0021675 | nerve development | 24/2872 | 55/13036 | 1.98 | 0.000278449 | 0.002415962 | BP |
| GO:0043367 | CD4-positive, alpha-beta T cell differentiation | 24/2872 | 55/13036 | 1.98 | 0.000278449 | 0.002415962 | BP |
| GO:0033280 | response to vitamin D | 16/2872 | 31/13036 | 2.34 | 0.00028651 | 0.002480719 | BP |
| GO:0050863 | regulation of T cell activation | 74/2872 | 232/13036 | 1.45 | 0.000291216 | 0.002516207 | BP |
| GO:0022602 | ovulation cycle process | 17/2872 | 34/13036 | 2.27 | 0.000304311 | 0.002607622 | BP |
| GO:0030195 | negative regulation of blood coagulation | 17/2872 | 34/13036 | 2.27 | 0.000304311 | 0.002607622 | BP |
| GO:0061900 | glial cell activation | 17/2872 | 34/13036 | 2.27 | 0.000304311 | 0.002607622 | BP |
| GO:0086002 | cardiac muscle cell action potential involved in contraction | 17/2872 | 34/13036 | 2.27 | 0.000304311 | 0.002607622 | BP |
| GO:0050867 | positive regulation of cell activation | 80/2872 | 255/13036 | 1.42 | 0.000305162 | 0.002609523 | BP |
| GO:2000379 | positive regulation of reactive oxygen species metabolic process | 31/2872 | 78/13036 | 1.8 | 0.000305956 | 0.002610931 | BP |
| GO:0031667 | response to nutrient levels | 109/2872 | 367/13036 | 1.35 | 0.000310692 | 0.002642725 | BP |
| GO:0071398 | cellular response to fatty acid | 21/2872 | 46/13036 | 2.07 | 0.000311593 | 0.002642725 | BP |
| GO:1903524 | positive regulation of blood circulation | 21/2872 | 46/13036 | 2.07 | 0.000311593 | 0.002642725 | BP |
| GO:1902893 | regulation of pri-miRNA transcription by RNA polymerase II | 20/2872 | 43/13036 | 2.11 | 0.000317179 | 0.002683887 | BP |
| GO:0051937 | catecholamine transport | 19/2872 | 40/13036 | 2.16 | 0.000318544 | 0.002683887 | BP |
| GO:0055008 | cardiac muscle tissue morphogenesis | 19/2872 | 40/13036 | 2.16 | 0.000318544 | 0.002683887 | BP |
| GO:0006813 | potassium ion transport | 48/2872 | 137/13036 | 1.59 | 0.000319035 | 0.002683887 | BP |
| GO:0002250 | adaptive immune response | 82/2872 | 263/13036 | 1.42 | 0.000322058 | 0.00270383 | BP |
| GO:0014066 | regulation of phosphatidylinositol 3-kinase signaling | 38/2872 | 102/13036 | 1.69 | 0.000327539 | 0.002744295 | BP |
| GO:0051249 | regulation of lymphocyte activation | 96/2872 | 317/13036 | 1.37 | 0.000332294 | 0.002778519 | BP |
| GO:0046849 | bone remodeling | 30/2872 | 75/13036 | 1.82 | 0.000333416 | 0.002781315 | BP |
| GO:0003338 | metanephros morphogenesis | 13/2872 | 23/13036 | 2.57 | 0.000335311 | 0.002781315 | BP |
| GO:0021983 | pituitary gland development | 13/2872 | 23/13036 | 2.57 | 0.000335311 | 0.002781315 | BP |
| GO:0046697 | decidualization | 13/2872 | 23/13036 | 2.57 | 0.000335311 | 0.002781315 | BP |
| GO:0002718 | regulation of cytokine production involved in immune response | 26/2872 | 62/13036 | 1.9 | 0.000340515 | 0.002807636 | BP |
| GO:0061180 | mammary gland epithelium development | 26/2872 | 62/13036 | 1.9 | 0.000340515 | 0.002807636 | BP |
| GO:2001057 | reactive nitrogen species metabolic process | 26/2872 | 62/13036 | 1.9 | 0.000340515 | 0.002807636 | BP |
| GO:0002688 | regulation of leukocyte chemotaxis | 35/2872 | 92/13036 | 1.73 | 0.000350245 | 0.002868689 | BP |
| GO:0003231 | cardiac ventricle development | 35/2872 | 92/13036 | 1.73 | 0.000350245 | 0.002868689 | BP |
| GO:0055007 | cardiac muscle cell differentiation | 35/2872 | 92/13036 | 1.73 | 0.000350245 | 0.002868689 | BP |
| GO:0032103 | positive regulation of response to external stimulus | 110/2872 | 372/13036 | 1.34 | 0.000350686 | 0.002868689 | BP |
| GO:0043491 | protein kinase B signaling | 64/2872 | 196/13036 | 1.48 | 0.000357916 | 0.002915085 | BP |
| GO:0010935 | regulation of macrophage cytokine production | 9/2872 | 13/13036 | 3.14 | 0.000359872 | 0.002915085 | BP |
| GO:0043116 | negative regulation of vascular permeability | 9/2872 | 13/13036 | 3.14 | 0.000359872 | 0.002915085 | BP |
| GO:0051953 | negative regulation of amine transport | 9/2872 | 13/13036 | 3.14 | 0.000359872 | 0.002915085 | BP |
| GO:0070293 | renal absorption | 9/2872 | 13/13036 | 3.14 | 0.000359872 | 0.002915085 | BP |
| GO:0046209 | nitric oxide metabolic process | 25/2872 | 59/13036 | 1.92 | 0.000363642 | 0.002939875 | BP |
| GO:0001654 | eye development | 88/2872 | 287/13036 | 1.39 | 0.00036914 | 0.00297852 | BP |
| GO:0032963 | collagen metabolic process | 34/2872 | 89/13036 | 1.73 | 0.000386259 | 0.003110602 | BP |
| GO:0016048 | detection of temperature stimulus | 11/2872 | 18/13036 | 2.77 | 0.000389327 | 0.003127658 | BP |
| GO:0048566 | embryonic digestive tract development | 14/2872 | 26/13036 | 2.44 | 0.000389886 | 0.003127658 | BP |
| GO:0061045 | negative regulation of wound healing | 23/2872 | 53/13036 | 1.97 | 0.000408617 | 0.003271592 | BP |
| GO:1904062 | regulation of cation transmembrane transport | 76/2872 | 242/13036 | 1.43 | 0.000411457 | 0.003284556 | BP |
| GO:1901861 | regulation of muscle tissue development | 42/2872 | 117/13036 | 1.63 | 0.00041182 | 0.003284556 | BP |
| GO:0002696 | positive regulation of leukocyte activation | 77/2872 | 246/13036 | 1.42 | 0.000423115 | 0.003368163 | BP |
| GO:1905332 | positive regulation of morphogenesis of an epithelium | 15/2872 | 29/13036 | 2.35 | 0.00043001 | 0.003416491 | BP |
| GO:0006721 | terpenoid metabolic process | 30/2872 | 76/13036 | 1.79 | 0.000436188 | 0.003458955 | BP |
| GO:0007269 | neurotransmitter secretion | 45/2872 | 128/13036 | 1.6 | 0.000443666 | 0.003504853 | BP |
| GO:0099643 | signal release from synapse | 45/2872 | 128/13036 | 1.6 | 0.000443666 | 0.003504853 | BP |
| GO:0048645 | animal organ formation | 21/2872 | 47/13036 | 2.03 | 0.0004474 | 0.003514269 | BP |
| GO:1904705 | regulation of vascular associated smooth muscle cell proliferation | 21/2872 | 47/13036 | 2.03 | 0.0004474 | 0.003514269 | BP |
| GO:1990874 | vascular associated smooth muscle cell proliferation | 21/2872 | 47/13036 | 2.03 | 0.0004474 | 0.003514269 | BP |
| GO:0046631 | alpha-beta T cell activation | 37/2872 | 100/13036 | 1.68 | 0.000455266 | 0.003569295 | BP |
| GO:0002063 | chondrocyte development | 16/2872 | 32/13036 | 2.27 | 0.000456529 | 0.003569822 | BP |
| GO:0016202 | regulation of striated muscle tissue development | 41/2872 | 114/13036 | 1.63 | 0.000458705 | 0.003569822 | BP |
| GO:2000241 | regulation of reproductive process | 41/2872 | 114/13036 | 1.63 | 0.000458705 | 0.003569822 | BP |
| GO:0051899 | membrane depolarization | 26/2872 | 63/13036 | 1.87 | 0.000459637 | 0.003569822 | BP |
| GO:1903035 | negative regulation of response to wounding | 26/2872 | 63/13036 | 1.87 | 0.000459637 | 0.003569822 | BP |
| GO:0003208 | cardiac ventricle morphogenesis | 20/2872 | 44/13036 | 2.06 | 0.000462118 | 0.003582383 | BP |
| GO:0070268 | cornification | 17/2872 | 35/13036 | 2.2 | 0.000471091 | 0.003623684 | BP |
| GO:0051965 | positive regulation of synapse assembly | 19/2872 | 41/13036 | 2.1 | 0.000472042 | 0.003623684 | BP |
| GO:0033674 | positive regulation of kinase activity | 141/2872 | 498/13036 | 1.29 | 0.000472242 | 0.003623684 | BP |
| GO:0045217 | cell-cell junction maintenance | 8/2872 | 11/13036 | 3.3 | 0.000474436 | 0.003623684 | BP |
| GO:0046068 | cGMP metabolic process | 8/2872 | 11/13036 | 3.3 | 0.000474436 | 0.003623684 | BP |
| GO:0060099 | regulation of phagocytosis, engulfment | 8/2872 | 11/13036 | 3.3 | 0.000474436 | 0.003623684 | BP |
| GO:0060100 | positive regulation of phagocytosis, engulfment | 8/2872 | 11/13036 | 3.3 | 0.000474436 | 0.003623684 | BP |
| GO:1905155 | positive regulation of membrane invagination | 8/2872 | 11/13036 | 3.3 | 0.000474436 | 0.003623684 | BP |
| GO:0007588 | excretion | 18/2872 | 38/13036 | 2.15 | 0.000475622 | 0.003626063 | BP |
| GO:0019722 | calcium-mediated signaling | 51/2872 | 150/13036 | 1.54 | 0.000479028 | 0.00364533 | BP |
| GO:0048592 | eye morphogenesis | 44/2872 | 125/13036 | 1.6 | 0.000495217 | 0.003761629 | BP |
| GO:0035116 | embryonic hindlimb morphogenesis | 12/2872 | 21/13036 | 2.59 | 0.00049854 | 0.003773048 | BP |
| GO:0070528 | protein kinase C signaling | 12/2872 | 21/13036 | 2.59 | 0.00049854 | 0.003773048 | BP |
| GO:0007159 | leukocyte cell-cell adhesion | 79/2872 | 255/13036 | 1.41 | 0.00050894 | 0.003844737 | BP |
| GO:0062013 | positive regulation of small molecule metabolic process | 40/2872 | 111/13036 | 1.64 | 0.000510868 | 0.003852285 | BP |
| GO:0072676 | lymphocyte migration | 28/2872 | 70/13036 | 1.82 | 0.000519627 | 0.003911222 | BP |
| GO:0048167 | regulation of synaptic plasticity | 50/2872 | 147/13036 | 1.54 | 0.000535665 | 0.004024638 | BP |
| GO:0008406 | gonad development | 56/2872 | 169/13036 | 1.5 | 0.000541124 | 0.004058298 | BP |
| GO:0006720 | isoprenoid metabolic process | 35/2872 | 94/13036 | 1.69 | 0.000559861 | 0.004191242 | BP |
| GO:0048634 | regulation of muscle organ development | 41/2872 | 115/13036 | 1.62 | 0.000563356 | 0.004199101 | BP |
| GO:0060065 | uterus development | 10/2872 | 16/13036 | 2.84 | 0.000564186 | 0.004199101 | BP |
| GO:2000738 | positive regulation of stem cell differentiation | 10/2872 | 16/13036 | 2.84 | 0.000564186 | 0.004199101 | BP |
| GO:0003073 | regulation of systemic arterial blood pressure | 23/2872 | 54/13036 | 1.93 | 0.00056496 | 0.004199101 | BP |
| GO:0070665 | positive regulation of leukocyte proliferation | 37/2872 | 101/13036 | 1.66 | 0.000568154 | 0.004208522 | BP |
| GO:0030216 | keratinocyte differentiation | 39/2872 | 108/13036 | 1.64 | 0.000568887 | 0.004208522 | BP |
| GO:0045137 | development of primary sexual characteristics | 57/2872 | 173/13036 | 1.5 | 0.000569272 | 0.004208522 | BP |
| GO:0010038 | response to metal ion | 85/2872 | 279/13036 | 1.38 | 0.00057592 | 0.00425009 | BP |
| GO:0060350 | endochondral bone morphogenesis | 22/2872 | 51/13036 | 1.96 | 0.000599384 | 0.004415396 | BP |
| GO:0070663 | regulation of leukocyte proliferation | 52/2872 | 155/13036 | 1.52 | 0.000606362 | 0.004458874 | BP |
| GO:0048588 | developmental cell growth | 62/2872 | 192/13036 | 1.47 | 0.000608346 | 0.004465552 | BP |
| GO:0032755 | positive regulation of interleukin-6 production | 26/2872 | 64/13036 | 1.84 | 0.000613804 | 0.00449765 | BP |
| GO:0009582 | detection of abiotic stimulus | 34/2872 | 91/13036 | 1.7 | 0.000620433 | 0.004538207 | BP |
| GO:1902305 | regulation of sodium ion transmembrane transport | 21/2872 | 48/13036 | 1.99 | 0.000631916 | 0.004614064 | BP |
| GO:0001952 | regulation of cell-matrix adhesion | 38/2872 | 105/13036 | 1.64 | 0.000633402 | 0.004616787 | BP |
| GO:0042531 | positive regulation of tyrosine phosphorylation of STAT protein | 20/2872 | 45/13036 | 2.02 | 0.000661214 | 0.004802626 | BP |
| GO:0061614 | pri-miRNA transcription by RNA polymerase II | 20/2872 | 45/13036 | 2.02 | 0.000661214 | 0.004802626 | BP |
| GO:0030279 | negative regulation of ossification | 25/2872 | 61/13036 | 1.86 | 0.000664473 | 0.004809452 | BP |
| GO:0060411 | cardiac septum morphogenesis | 25/2872 | 61/13036 | 1.86 | 0.000664473 | 0.004809452 | BP |
| GO:0030510 | regulation of BMP signaling pathway | 28/2872 | 71/13036 | 1.79 | 0.000679924 | 0.004904169 | BP |
| GO:0045582 | positive regulation of T cell differentiation | 28/2872 | 71/13036 | 1.79 | 0.000679924 | 0.004904169 | BP |
| GO:0010464 | regulation of mesenchymal cell proliferation | 15/2872 | 30/13036 | 2.27 | 0.000685955 | 0.004939073 | BP |
| GO:0050866 | negative regulation of cell activation | 46/2872 | 134/13036 | 1.56 | 0.000699632 | 0.005009455 | BP |
| GO:0061337 | cardiac conduction | 35/2872 | 95/13036 | 1.67 | 0.000701328 | 0.005009455 | BP |
| GO:0032613 | interleukin-10 production | 18/2872 | 39/13036 | 2.09 | 0.000702977 | 0.005009455 | BP |
| GO:0048662 | negative regulation of smooth muscle cell proliferation | 18/2872 | 39/13036 | 2.09 | 0.000702977 | 0.005009455 | BP |
| GO:0050919 | negative chemotaxis | 18/2872 | 39/13036 | 2.09 | 0.000702977 | 0.005009455 | BP |
| GO:2000242 | negative regulation of reproductive process | 18/2872 | 39/13036 | 2.09 | 0.000702977 | 0.005009455 | BP |
| GO:0030048 | actin filament-based movement | 37/2872 | 102/13036 | 1.65 | 0.00070512 | 0.00501611 | BP |
| GO:0090092 | regulation of transmembrane receptor protein serine/threonine kinase signaling pathway | 62/2872 | 193/13036 | 1.46 | 0.000706908 | 0.005020216 | BP |
| GO:0071248 | cellular response to metal ion | 49/2872 | 145/13036 | 1.53 | 0.000714434 | 0.005064991 | BP |
| GO:0045861 | negative regulation of proteolysis | 77/2872 | 250/13036 | 1.4 | 0.000719303 | 0.005090807 | BP |
| GO:0022898 | regulation of transmembrane transporter activity | 63/2872 | 197/13036 | 1.45 | 0.000733482 | 0.005182317 | BP |
| GO:0007263 | nitric oxide mediated signal transduction | 11/2872 | 19/13036 | 2.63 | 0.000740917 | 0.00521707 | BP |
| GO:0072273 | metanephric nephron morphogenesis | 11/2872 | 19/13036 | 2.63 | 0.000740917 | 0.00521707 | BP |
| GO:0035710 | CD4-positive, alpha-beta T cell activation | 27/2872 | 68/13036 | 1.8 | 0.000743432 | 0.005225911 | BP |
| GO:0050769 | positive regulation of neurogenesis | 114/2872 | 395/13036 | 1.31 | 0.000751883 | 0.005276374 | BP |
| GO:0022409 | positive regulation of cell-cell adhesion | 64/2872 | 201/13036 | 1.45 | 0.000759646 | 0.005321846 | BP |
| GO:0007264 | small GTPase mediated signal transduction | 128/2872 | 451/13036 | 1.29 | 0.000762739 | 0.005334503 | BP |
| GO:0050954 | sensory perception of mechanical stimulus | 40/2872 | 113/13036 | 1.61 | 0.000768664 | 0.005364373 | BP |
| GO:0001708 | cell fate specification | 23/2872 | 55/13036 | 1.9 | 0.00077089 | 0.005364373 | BP |
| GO:0051058 | negative regulation of small GTPase mediated signal transduction | 23/2872 | 55/13036 | 1.9 | 0.00077089 | 0.005364373 | BP |
| GO:0010544 | negative regulation of platelet activation | 9/2872 | 14/13036 | 2.92 | 0.000810375 | 0.005593889 | BP |
| GO:0010744 | positive regulation of macrophage derived foam cell differentiation | 9/2872 | 14/13036 | 2.92 | 0.000810375 | 0.005593889 | BP |
| GO:0051412 | response to corticosterone | 9/2872 | 14/13036 | 2.92 | 0.000810375 | 0.005593889 | BP |
| GO:0090190 | positive regulation of branching involved in ureteric bud morphogenesis | 9/2872 | 14/13036 | 2.92 | 0.000810375 | 0.005593889 | BP |
| GO:0070372 | regulation of ERK1 and ERK2 cascade | 66/2872 | 209/13036 | 1.43 | 0.000810617 | 0.005593889 | BP |
| GO:1902107 | positive regulation of leukocyte differentiation | 43/2872 | 124/13036 | 1.57 | 0.000812322 | 0.005596342 | BP |
| GO:0016049 | cell growth | 117/2872 | 408/13036 | 1.3 | 0.000836098 | 0.005750591 | BP |
| GO:0043524 | negative regulation of neuron apoptotic process | 41/2872 | 117/13036 | 1.59 | 0.000838011 | 0.005754203 | BP |
| GO:0006814 | sodium ion transport | 49/2872 | 146/13036 | 1.52 | 0.000849146 | 0.005821027 | BP |
| GO:0071692 | protein localization to extracellular region | 98/2872 | 333/13036 | 1.34 | 0.000853369 | 0.005840322 | BP |
| GO:0060078 | regulation of postsynaptic membrane potential | 33/2872 | 89/13036 | 1.68 | 0.000864547 | 0.005907074 | BP |
| GO:0021988 | olfactory lobe development | 12/2872 | 22/13036 | 2.48 | 0.000877761 | 0.005966301 | BP |
| GO:0050892 | intestinal absorption | 12/2872 | 22/13036 | 2.48 | 0.000877761 | 0.005966301 | BP |
| GO:0150077 | regulation of neuroinflammatory response | 12/2872 | 22/13036 | 2.48 | 0.000877761 | 0.005966301 | BP |
| GO:0002690 | positive regulation of leukocyte chemotaxis | 28/2872 | 72/13036 | 1.77 | 0.000881847 | 0.005966301 | BP |
| GO:0030301 | cholesterol transport | 28/2872 | 72/13036 | 1.77 | 0.000881847 | 0.005966301 | BP |
| GO:0060191 | regulation of lipase activity | 28/2872 | 72/13036 | 1.77 | 0.000881847 | 0.005966301 | BP |
| GO:0003151 | outflow tract morphogenesis | 25/2872 | 62/13036 | 1.83 | 0.000883364 | 0.005966835 | BP |
| GO:0015672 | monovalent inorganic cation transport | 105/2872 | 361/13036 | 1.32 | 0.000891455 | 0.006011696 | BP |
| GO:0045444 | fat cell differentiation | 61/2872 | 191/13036 | 1.45 | 0.000915613 | 0.006164585 | BP |
| GO:0071219 | cellular response to molecule of bacterial origin | 51/2872 | 154/13036 | 1.5 | 0.000948256 | 0.006374016 | BP |
| GO:0014855 | striated muscle cell proliferation | 24/2872 | 59/13036 | 1.85 | 0.000959363 | 0.006427836 | BP |
| GO:0055013 | cardiac muscle cell development | 24/2872 | 59/13036 | 1.85 | 0.000959363 | 0.006427836 | BP |
| GO:0016101 | diterpenoid metabolic process | 27/2872 | 69/13036 | 1.78 | 0.000968394 | 0.006464185 | BP |
| GO:0032946 | positive regulation of mononuclear cell proliferation | 34/2872 | 93/13036 | 1.66 | 0.000971643 | 0.006464185 | BP |
| GO:0009435 | NAD biosynthetic process | 13/2872 | 25/13036 | 2.36 | 0.000973602 | 0.006464185 | BP |
| GO:0009595 | detection of biotic stimulus | 13/2872 | 25/13036 | 2.36 | 0.000973602 | 0.006464185 | BP |
| GO:0010743 | regulation of macrophage derived foam cell differentiation | 13/2872 | 25/13036 | 2.36 | 0.000973602 | 0.006464185 | BP |
| GO:0002790 | peptide secretion | 102/2872 | 350/13036 | 1.32 | 0.000974839 | 0.006464185 | BP |
| GO:0045540 | regulation of cholesterol biosynthetic process | 19/2872 | 43/13036 | 2.01 | 0.000977257 | 0.006464185 | BP |
| GO:0106118 | regulation of sterol biosynthetic process | 19/2872 | 43/13036 | 2.01 | 0.000977257 | 0.006464185 | BP |
| GO:0009914 | hormone transport | 69/2872 | 222/13036 | 1.41 | 0.001010554 | 0.006673791 | BP |
| GO:0021536 | diencephalon development | 18/2872 | 40/13036 | 2.04 | 0.001017512 | 0.006698408 | BP |
| GO:0085029 | extracellular matrix assembly | 18/2872 | 40/13036 | 2.04 | 0.001017512 | 0.006698408 | BP |
| GO:0002064 | epithelial cell development | 56/2872 | 173/13036 | 1.47 | 0.001020112 | 0.006704883 | BP |
| GO:0043542 | endothelial cell migration | 60/2872 | 188/13036 | 1.45 | 0.001023063 | 0.006713641 | BP |
| GO:0014812 | muscle cell migration | 29/2872 | 76/13036 | 1.73 | 0.001027626 | 0.006722308 | BP |
| GO:0060021 | roof of mouth development | 29/2872 | 76/13036 | 1.73 | 0.001027626 | 0.006722308 | BP |
| GO:0003044 | regulation of systemic arterial blood pressure mediated by a chemical signal | 14/2872 | 28/13036 | 2.27 | 0.001032506 | 0.006743597 | BP |
| GO:0032409 | regulation of transporter activity | 65/2872 | 207/13036 | 1.43 | 0.001040382 | 0.006767837 | BP |
| GO:0001936 | regulation of endothelial cell proliferation | 39/2872 | 111/13036 | 1.59 | 0.001045953 | 0.006767837 | BP |
| GO:0002448 | mast cell mediated immunity | 17/2872 | 37/13036 | 2.09 | 0.001047641 | 0.006767837 | BP |
| GO:0030574 | collagen catabolic process | 17/2872 | 37/13036 | 2.09 | 0.001047641 | 0.006767837 | BP |
| GO:0032570 | response to progesterone | 17/2872 | 37/13036 | 2.09 | 0.001047641 | 0.006767837 | BP |
| GO:0070169 | positive regulation of biomineral tissue development | 17/2872 | 37/13036 | 2.09 | 0.001047641 | 0.006767837 | BP |
| GO:0110151 | positive regulation of biomineralization | 17/2872 | 37/13036 | 2.09 | 0.001047641 | 0.006767837 | BP |
| GO:0022408 | negative regulation of cell-cell adhesion | 44/2872 | 129/13036 | 1.55 | 0.00104931 | 0.00676808 | BP |
| GO:0032722 | positive regulation of chemokine production | 15/2872 | 31/13036 | 2.2 | 0.001060433 | 0.006799099 | BP |
| GO:0035136 | forelimb morphogenesis | 15/2872 | 31/13036 | 2.2 | 0.001060433 | 0.006799099 | BP |
| GO:0002686 | negative regulation of leukocyte migration | 16/2872 | 34/13036 | 2.14 | 0.001063581 | 0.006799099 | BP |
| GO:0043090 | amino acid import | 16/2872 | 34/13036 | 2.14 | 0.001063581 | 0.006799099 | BP |
| GO:0050707 | regulation of cytokine secretion | 16/2872 | 34/13036 | 2.14 | 0.001063581 | 0.006799099 | BP |
| GO:0010717 | regulation of epithelial to mesenchymal transition | 31/2872 | 83/13036 | 1.7 | 0.001063955 | 0.006799099 | BP |
| GO:0071805 | potassium ion transmembrane transport | 42/2872 | 122/13036 | 1.56 | 0.001094635 | 0.006984392 | BP |
| GO:0050951 | sensory perception of temperature stimulus | 10/2872 | 17/13036 | 2.67 | 0.001100096 | 0.006997704 | BP |
| GO:0060384 | innervation | 10/2872 | 17/13036 | 2.67 | 0.001100096 | 0.006997704 | BP |
| GO:0010975 | regulation of neuron projection development | 123/2872 | 435/13036 | 1.28 | 0.001113247 | 0.007070511 | BP |
| GO:0033273 | response to vitamin | 28/2872 | 73/13036 | 1.74 | 0.001134047 | 0.007180625 | BP |
| GO:0055024 | regulation of cardiac muscle tissue development | 28/2872 | 73/13036 | 1.74 | 0.001134047 | 0.007180625 | BP |
| GO:0032693 | negative regulation of interleukin-10 production | 8/2872 | 12/13036 | 3.03 | 0.001147755 | 0.007223314 | BP |
| GO:0072498 | embryonic skeletal joint development | 8/2872 | 12/13036 | 3.03 | 0.001147755 | 0.007223314 | BP |
| GO:0072677 | eosinophil migration | 8/2872 | 12/13036 | 3.03 | 0.001147755 | 0.007223314 | BP |
| GO:2000696 | regulation of epithelial cell differentiation involved in kidney development | 8/2872 | 12/13036 | 3.03 | 0.001147755 | 0.007223314 | BP |
| GO:0002683 | negative regulation of immune system process | 95/2872 | 324/13036 | 1.33 | 0.001155761 | 0.007262677 | BP |
| GO:0032526 | response to retinoic acid | 30/2872 | 80/13036 | 1.7 | 0.001179391 | 0.007392629 | BP |
| GO:0051056 | regulation of small GTPase mediated signal transduction | 85/2872 | 285/13036 | 1.35 | 0.001180006 | 0.007392629 | BP |
| GO:0051235 | maintenance of location | 77/2872 | 254/13036 | 1.38 | 0.001189436 | 0.007440468 | BP |
| GO:1901655 | cellular response to ketone | 32/2872 | 87/13036 | 1.67 | 0.001201725 | 0.007506016 | BP |
| GO:0032602 | chemokine production | 21/2872 | 50/13036 | 1.91 | 0.001204361 | 0.007511169 | BP |
| GO:0007266 | Rho protein signal transduction | 41/2872 | 119/13036 | 1.56 | 0.001224543 | 0.007625569 | BP |
| GO:0007589 | body fluid secretion | 27/2872 | 70/13036 | 1.75 | 0.001250179 | 0.007750301 | BP |
| GO:0014031 | mesenchymal cell development | 27/2872 | 70/13036 | 1.75 | 0.001250179 | 0.007750301 | BP |
| GO:1903845 | negative regulation of cellular response to transforming growth factor beta stimulus | 27/2872 | 70/13036 | 1.75 | 0.001250179 | 0.007750301 | BP |
| GO:0090288 | negative regulation of cellular response to growth factor stimulus | 44/2872 | 130/13036 | 1.54 | 0.001253529 | 0.007759472 | BP |
| GO:0045666 | positive regulation of neuron differentiation | 92/2872 | 313/13036 | 1.33 | 0.001260456 | 0.007790725 | BP |
| GO:0032642 | regulation of chemokine production | 20/2872 | 47/13036 | 1.93 | 0.001287748 | 0.007935761 | BP |
| GO:0045428 | regulation of nitric oxide biosynthetic process | 20/2872 | 47/13036 | 1.93 | 0.001287748 | 0.007935761 | BP |
| GO:0062149 | detection of stimulus involved in sensory perception of pain | 11/2872 | 20/13036 | 2.5 | 0.001320025 | 0.008122593 | BP |
| GO:0072593 | reactive oxygen species metabolic process | 64/2872 | 205/13036 | 1.42 | 0.001331594 | 0.008174914 | BP |
| GO:0010634 | positive regulation of epithelial cell migration | 45/2872 | 134/13036 | 1.52 | 0.00133247 | 0.008174914 | BP |
| GO:0015918 | sterol transport | 31/2872 | 84/13036 | 1.68 | 0.001335835 | 0.008183455 | BP |
| GO:0030308 | negative regulation of cell growth | 52/2872 | 160/13036 | 1.48 | 0.00136868 | 0.008335919 | BP |
| GO:0050663 | cytokine secretion | 19/2872 | 44/13036 | 1.96 | 0.001368762 | 0.008335919 | BP |
| GO:0051353 | positive regulation of oxidoreductase activity | 19/2872 | 44/13036 | 1.96 | 0.001368762 | 0.008335919 | BP |
| GO:0051966 | regulation of synaptic transmission, glutamatergic | 19/2872 | 44/13036 | 1.96 | 0.001368762 | 0.008335919 | BP |
| GO:0032612 | interleukin-1 production | 26/2872 | 67/13036 | 1.76 | 0.001376555 | 0.008362226 | BP |
| GO:0002791 | regulation of peptide secretion | 79/2872 | 263/13036 | 1.36 | 0.001382408 | 0.008362226 | BP |
| GO:0006026 | aminoglycan catabolic process | 23/2872 | 57/13036 | 1.83 | 0.001383163 | 0.008362226 | BP |
| GO:0007260 | tyrosine phosphorylation of STAT protein | 23/2872 | 57/13036 | 1.83 | 0.001383163 | 0.008362226 | BP |
| GO:0014910 | regulation of smooth muscle cell migration | 23/2872 | 57/13036 | 1.83 | 0.001383163 | 0.008362226 | BP |
| GO:0006636 | unsaturated fatty acid biosynthetic process | 18/2872 | 41/13036 | 1.99 | 0.001444402 | 0.008706881 | BP |
| GO:1903428 | positive regulation of reactive oxygen species biosynthetic process | 18/2872 | 41/13036 | 1.99 | 0.001444402 | 0.008706881 | BP |
| GO:0055017 | cardiac muscle tissue growth | 28/2872 | 74/13036 | 1.72 | 0.001446469 | 0.008706881 | BP |
| GO:0032147 | activation of protein kinase activity | 83/2872 | 279/13036 | 1.35 | 0.001451403 | 0.008723915 | BP |
| GO:0070482 | response to oxygen levels | 99/2872 | 342/13036 | 1.31 | 0.001456349 | 0.008740977 | BP |
| GO:0032770 | positive regulation of monooxygenase activity | 12/2872 | 23/13036 | 2.37 | 0.001469525 | 0.008807315 | BP |
| GO:0010001 | glial cell differentiation | 58/2872 | 183/13036 | 1.44 | 0.001475989 | 0.008833288 | BP |
| GO:0006809 | nitric oxide biosynthetic process | 22/2872 | 54/13036 | 1.85 | 0.00150299 | 0.008981919 | BP |
| GO:0030225 | macrophage differentiation | 17/2872 | 38/13036 | 2.03 | 0.001510704 | 0.009015028 | BP |
| GO:0014909 | smooth muscle cell migration | 25/2872 | 64/13036 | 1.77 | 0.001513624 | 0.009019479 | BP |
| GO:1903037 | regulation of leukocyte cell-cell adhesion | 70/2872 | 229/13036 | 1.39 | 0.001525444 | 0.009076871 | BP |
| GO:0031032 | actomyosin structure organization | 55/2872 | 172/13036 | 1.45 | 0.001540604 | 0.009140848 | BP |
| GO:0060541 | respiratory system development | 55/2872 | 172/13036 | 1.45 | 0.001540604 | 0.009140848 | BP |
| GO:0001774 | microglial cell activation | 13/2872 | 26/13036 | 2.27 | 0.001557318 | 0.009200528 | BP |
| GO:0002269 | leukocyte activation involved in inflammatory response | 13/2872 | 26/13036 | 2.27 | 0.001557318 | 0.009200528 | BP |
| GO:0010092 | specification of animal organ identity | 13/2872 | 26/13036 | 2.27 | 0.001557318 | 0.009200528 | BP |
| GO:0001935 | endothelial cell proliferation | 42/2872 | 124/13036 | 1.54 | 0.001571011 | 0.009212221 | BP |
| GO:0001977 | renal system process involved in regulation of blood volume | 7/2872 | 10/13036 | 3.18 | 0.001587555 | 0.009212221 | BP |
| GO:0007494 | midgut development | 7/2872 | 10/13036 | 3.18 | 0.001587555 | 0.009212221 | BP |
| GO:0014821 | phasic smooth muscle contraction | 7/2872 | 10/13036 | 3.18 | 0.001587555 | 0.009212221 | BP |
| GO:0021561 | facial nerve development | 7/2872 | 10/13036 | 3.18 | 0.001587555 | 0.009212221 | BP |
| GO:0021610 | facial nerve morphogenesis | 7/2872 | 10/13036 | 3.18 | 0.001587555 | 0.009212221 | BP |
| GO:0033089 | positive regulation of T cell differentiation in thymus | 7/2872 | 10/13036 | 3.18 | 0.001587555 | 0.009212221 | BP |
| GO:0042976 | activation of Janus kinase activity | 7/2872 | 10/13036 | 3.18 | 0.001587555 | 0.009212221 | BP |
| GO:0048934 | peripheral nervous system neuron differentiation | 7/2872 | 10/13036 | 3.18 | 0.001587555 | 0.009212221 | BP |
| GO:0048935 | peripheral nervous system neuron development | 7/2872 | 10/13036 | 3.18 | 0.001587555 | 0.009212221 | BP |
| GO:0099550 | trans-synaptic signaling, modulating synaptic transmission | 7/2872 | 10/13036 | 3.18 | 0.001587555 | 0.009212221 | BP |
| GO:0035633 | maintenance of blood-brain barrier | 15/2872 | 32/13036 | 2.13 | 0.00159329 | 0.009212221 | BP |
| GO:0002673 | regulation of acute inflammatory response | 14/2872 | 29/13036 | 2.19 | 0.001594836 | 0.009212221 | BP |
| GO:0030890 | positive regulation of B cell proliferation | 14/2872 | 29/13036 | 2.19 | 0.001594836 | 0.009212221 | BP |
| GO:0060317 | cardiac epithelial to mesenchymal transition | 14/2872 | 29/13036 | 2.19 | 0.001594836 | 0.009212221 | BP |
| GO:0098868 | bone growth | 14/2872 | 29/13036 | 2.19 | 0.001594836 | 0.009212221 | BP |
| GO:0021510 | spinal cord development | 27/2872 | 71/13036 | 1.73 | 0.001600107 | 0.009229812 | BP |
| GO:0003071 | renal system process involved in regulation of systemic arterial blood pressure | 9/2872 | 15/13036 | 2.72 | 0.001630437 | 0.009339813 | BP |
| GO:0043901 | negative regulation of multi-organism process | 9/2872 | 15/13036 | 2.72 | 0.001630437 | 0.009339813 | BP |
| GO:0048486 | parasympathetic nervous system development | 9/2872 | 15/13036 | 2.72 | 0.001630437 | 0.009339813 | BP |
| GO:0061213 | positive regulation of mesonephros development | 9/2872 | 15/13036 | 2.72 | 0.001630437 | 0.009339813 | BP |
| GO:0072215 | regulation of metanephros development | 9/2872 | 15/13036 | 2.72 | 0.001630437 | 0.009339813 | BP |
| GO:0002367 | cytokine production involved in immune response | 29/2872 | 78/13036 | 1.69 | 0.001648739 | 0.009418633 | BP |
| GO:0099565 | chemical synaptic transmission, postsynaptic | 29/2872 | 78/13036 | 1.69 | 0.001648739 | 0.009418633 | BP |
| GO:0050671 | positive regulation of lymphocyte proliferation | 33/2872 | 92/13036 | 1.63 | 0.001657974 | 0.009458362 | BP |
| GO:0001666 | response to hypoxia | 90/2872 | 308/13036 | 1.33 | 0.001717812 | 0.009786264 | BP |
| GO:0010035 | response to inorganic substance | 124/2872 | 444/13036 | 1.27 | 0.001743486 | 0.009918529 | BP |
| GO:0036293 | response to decreased oxygen levels | 93/2872 | 320/13036 | 1.32 | 0.001745812 | 0.009918529 | BP |
| GO:0032412 | regulation of ion transmembrane transporter activity | 59/2872 | 188/13036 | 1.42 | 0.001751939 | 0.009939724 | BP |
| GO:0022604 | regulation of cell morphogenesis | 120/2872 | 428/13036 | 1.27 | 0.001765979 | 0.009992603 | BP |
| GO:0030512 | negative regulation of transforming growth factor beta receptor signaling pathway | 26/2872 | 68/13036 | 1.74 | 0.001768488 | 0.009992603 | BP |
| GO:0051591 | response to cAMP | 26/2872 | 68/13036 | 1.74 | 0.001768488 | 0.009992603 | BP |
| GO:0007179 | transforming growth factor beta receptor signaling pathway | 51/2872 | 158/13036 | 1.47 | 0.001785956 | 0.010077575 | BP |
| GO:0032611 | interleukin-1 beta production | 23/2872 | 58/13036 | 1.8 | 0.001820904 | 0.010260816 | BP |
| GO:0002720 | positive regulation of cytokine production involved in immune response | 19/2872 | 45/13036 | 1.92 | 0.001885816 | 0.010597798 | BP |
| GO:0048747 | muscle fiber development | 19/2872 | 45/13036 | 1.92 | 0.001885816 | 0.010597798 | BP |
| GO:0002687 | positive regulation of leukocyte migration | 37/2872 | 107/13036 | 1.57 | 0.001919908 | 0.010774783 | BP |
| GO:0045667 | regulation of osteoblast differentiation | 35/2872 | 100/13036 | 1.59 | 0.001985486 | 0.01102489 | BP |
| GO:1903510 | mucopolysaccharide metabolic process | 35/2872 | 100/13036 | 1.59 | 0.001985486 | 0.01102489 | BP |
| GO:0018149 | peptide cross-linking | 10/2872 | 18/13036 | 2.52 | 0.001988558 | 0.01102489 | BP |
| GO:0019433 | triglyceride catabolic process | 10/2872 | 18/13036 | 2.52 | 0.001988558 | 0.01102489 | BP |
| GO:0042474 | middle ear morphogenesis | 10/2872 | 18/13036 | 2.52 | 0.001988558 | 0.01102489 | BP |
| GO:0051968 | positive regulation of synaptic transmission, glutamatergic | 10/2872 | 18/13036 | 2.52 | 0.001988558 | 0.01102489 | BP |
| GO:0072202 | cell differentiation involved in metanephros development | 10/2872 | 18/13036 | 2.52 | 0.001988558 | 0.01102489 | BP |
| GO:0042509 | regulation of tyrosine phosphorylation of STAT protein | 22/2872 | 55/13036 | 1.82 | 0.001991056 | 0.01102489 | BP |
| GO:0055021 | regulation of cardiac muscle tissue growth | 22/2872 | 55/13036 | 1.82 | 0.001991056 | 0.01102489 | BP |
| GO:0060135 | maternal process involved in female pregnancy | 22/2872 | 55/13036 | 1.82 | 0.001991056 | 0.01102489 | BP |
| GO:0098657 | import into cell | 50/2872 | 155/13036 | 1.46 | 0.001998208 | 0.011049742 | BP |
| GO:0003279 | cardiac septum development | 33/2872 | 93/13036 | 1.61 | 0.002035456 | 0.011240727 | BP |
| GO:0043010 | camera-type eye development | 74/2872 | 247/13036 | 1.36 | 0.002074338 | 0.011440216 | BP |
| GO:0030888 | regulation of B cell proliferation | 17/2872 | 39/13036 | 1.98 | 0.002134856 | 0.011758348 | BP |
| GO:0071222 | cellular response to lipopolysaccharide | 48/2872 | 148/13036 | 1.47 | 0.002146412 | 0.011806315 | BP |
| GO:0001890 | placenta development | 42/2872 | 126/13036 | 1.51 | 0.002219088 | 0.012182728 | BP |
| GO:0061387 | regulation of extent of cell growth | 34/2872 | 97/13036 | 1.59 | 0.002221018 | 0.012182728 | BP |
| GO:0051043 | regulation of membrane protein ectodomain proteolysis | 11/2872 | 21/13036 | 2.38 | 0.002223657 | 0.012182728 | BP |
| GO:0006956 | complement activation | 16/2872 | 36/13036 | 2.02 | 0.002243856 | 0.012260998 | BP |
| GO:0032653 | regulation of interleukin-10 production | 16/2872 | 36/13036 | 2.02 | 0.002243856 | 0.012260998 | BP |
| GO:0050906 | detection of stimulus involved in sensory perception | 26/2872 | 69/13036 | 1.71 | 0.002252265 | 0.012290755 | BP |
| GO:0042063 | gliogenesis | 72/2872 | 240/13036 | 1.36 | 0.002273754 | 0.012391714 | BP |
| GO:0045621 | positive regulation of lymphocyte differentiation | 30/2872 | 83/13036 | 1.64 | 0.002303101 | 0.012532483 | BP |
| GO:0033135 | regulation of peptidyl-serine phosphorylation | 37/2872 | 108/13036 | 1.56 | 0.002311433 | 0.012532483 | BP |
| GO:0008361 | regulation of cell size | 50/2872 | 156/13036 | 1.45 | 0.002323207 | 0.012532483 | BP |
| GO:0021545 | cranial nerve development | 15/2872 | 33/13036 | 2.06 | 0.002332466 | 0.012532483 | BP |
| GO:0030212 | hyaluronan metabolic process | 15/2872 | 33/13036 | 2.06 | 0.002332466 | 0.012532483 | BP |
| GO:0030224 | monocyte differentiation | 15/2872 | 33/13036 | 2.06 | 0.002332466 | 0.012532483 | BP |
| GO:1903131 | mononuclear cell differentiation | 15/2872 | 33/13036 | 2.06 | 0.002332466 | 0.012532483 | BP |
| GO:0070588 | calcium ion transmembrane transport | 66/2872 | 217/13036 | 1.38 | 0.00234133 | 0.012532483 | BP |
| GO:1903305 | regulation of regulated secretory pathway | 40/2872 | 119/13036 | 1.53 | 0.002347525 | 0.012532483 | BP |
| GO:0003401 | axis elongation | 12/2872 | 24/13036 | 2.27 | 0.002354462 | 0.012532483 | BP |
| GO:0010543 | regulation of platelet activation | 12/2872 | 24/13036 | 2.27 | 0.002354462 | 0.012532483 | BP |
| GO:0086010 | membrane depolarization during action potential | 12/2872 | 24/13036 | 2.27 | 0.002354462 | 0.012532483 | BP |
| GO:0071216 | cellular response to biotic stimulus | 55/2872 | 175/13036 | 1.43 | 0.002370585 | 0.012532483 | BP |
| GO:0009798 | axis specification | 23/2872 | 59/13036 | 1.77 | 0.002371334 | 0.012532483 | BP |
| GO:1901863 | positive regulation of muscle tissue development | 23/2872 | 59/13036 | 1.77 | 0.002371334 | 0.012532483 | BP |
| GO:0006584 | catecholamine metabolic process | 14/2872 | 30/13036 | 2.12 | 0.002390108 | 0.012532483 | BP |
| GO:0009712 | catechol-containing compound metabolic process | 14/2872 | 30/13036 | 2.12 | 0.002390108 | 0.012532483 | BP |
| GO:0010742 | macrophage derived foam cell differentiation | 14/2872 | 30/13036 | 2.12 | 0.002390108 | 0.012532483 | BP |
| GO:0014047 | glutamate secretion | 14/2872 | 30/13036 | 2.12 | 0.002390108 | 0.012532483 | BP |
| GO:0090077 | foam cell differentiation | 14/2872 | 30/13036 | 2.12 | 0.002390108 | 0.012532483 | BP |
| GO:0032970 | regulation of actin filament-based process | 97/2872 | 339/13036 | 1.3 | 0.002392011 | 0.012532483 | BP |
| GO:0007218 | neuropeptide signaling pathway | 13/2872 | 27/13036 | 2.19 | 0.002403139 | 0.012532483 | BP |
| GO:0009954 | proximal/distal pattern formation | 13/2872 | 27/13036 | 2.19 | 0.002403139 | 0.012532483 | BP |
| GO:0031128 | developmental induction | 13/2872 | 27/13036 | 2.19 | 0.002403139 | 0.012532483 | BP |
| GO:0061082 | myeloid leukocyte cytokine production | 13/2872 | 27/13036 | 2.19 | 0.002403139 | 0.012532483 | BP |
| GO:0071295 | cellular response to vitamin | 13/2872 | 27/13036 | 2.19 | 0.002403139 | 0.012532483 | BP |
| GO:0072210 | metanephric nephron development | 13/2872 | 27/13036 | 2.19 | 0.002403139 | 0.012532483 | BP |
| GO:1905330 | regulation of morphogenesis of an epithelium | 51/2872 | 160/13036 | 1.45 | 0.002408138 | 0.012532483 | BP |
| GO:0002544 | chronic inflammatory response | 8/2872 | 13/13036 | 2.79 | 0.002408368 | 0.012532483 | BP |
| GO:0003215 | cardiac right ventricle morphogenesis | 8/2872 | 13/13036 | 2.79 | 0.002408368 | 0.012532483 | BP |
| GO:0035813 | regulation of renal sodium excretion | 8/2872 | 13/13036 | 2.79 | 0.002408368 | 0.012532483 | BP |
| GO:0048557 | embryonic digestive tract morphogenesis | 8/2872 | 13/13036 | 2.79 | 0.002408368 | 0.012532483 | BP |
| GO:0050930 | induction of positive chemotaxis | 8/2872 | 13/13036 | 2.79 | 0.002408368 | 0.012532483 | BP |
| GO:0060231 | mesenchymal to epithelial transition | 8/2872 | 13/13036 | 2.79 | 0.002408368 | 0.012532483 | BP |
| GO:0060572 | morphogenesis of an epithelial bud | 8/2872 | 13/13036 | 2.79 | 0.002408368 | 0.012532483 | BP |
| GO:0071371 | cellular response to gonadotropin stimulus | 8/2872 | 13/13036 | 2.79 | 0.002408368 | 0.012532483 | BP |
| GO:0051928 | positive regulation of calcium ion transport | 31/2872 | 87/13036 | 1.62 | 0.002541801 | 0.013210255 | BP |
| GO:0001706 | endoderm formation | 19/2872 | 46/13036 | 1.87 | 0.00255832 | 0.013253162 | BP |
| GO:0042490 | mechanoreceptor differentiation | 19/2872 | 46/13036 | 1.87 | 0.00255832 | 0.013253162 | BP |
| GO:0001649 | osteoblast differentiation | 58/2872 | 187/13036 | 1.41 | 0.002567659 | 0.013253162 | BP |
| GO:0001938 | positive regulation of endothelial cell proliferation | 29/2872 | 80/13036 | 1.65 | 0.00256923 | 0.013253162 | BP |
| GO:0046545 | development of primary female sexual characteristics | 29/2872 | 80/13036 | 1.65 | 0.00256923 | 0.013253162 | BP |
| GO:0046928 | regulation of neurotransmitter secretion | 29/2872 | 80/13036 | 1.65 | 0.00256923 | 0.013253162 | BP |
| GO:0007265 | Ras protein signal transduction | 88/2872 | 304/13036 | 1.31 | 0.00258633 | 0.0133248 | BP |
| GO:2000243 | positive regulation of reproductive process | 22/2872 | 56/13036 | 1.78 | 0.002607235 | 0.013415835 | BP |
| GO:0050708 | regulation of protein secretion | 74/2872 | 249/13036 | 1.35 | 0.002615938 | 0.013443938 | BP |
| GO:0009306 | protein secretion | 94/2872 | 328/13036 | 1.3 | 0.002628167 | 0.013468123 | BP |
| GO:0035592 | establishment of protein localization to extracellular region | 94/2872 | 328/13036 | 1.3 | 0.002628167 | 0.013468123 | BP |
| GO:0015718 | monocarboxylic acid transport | 39/2872 | 116/13036 | 1.53 | 0.002630386 | 0.013468123 | BP |
| GO:0032944 | regulation of mononuclear cell proliferation | 46/2872 | 142/13036 | 1.47 | 0.002689906 | 0.013755894 | BP |
| GO:0031424 | keratinization | 18/2872 | 43/13036 | 1.9 | 0.002759607 | 0.01409496 | BP |
| GO:0034103 | regulation of tissue remodeling | 24/2872 | 63/13036 | 1.73 | 0.002763224 | 0.014096075 | BP |
| GO:0016079 | synaptic vesicle exocytosis | 32/2872 | 91/13036 | 1.6 | 0.002780018 | 0.014164322 | BP |
| GO:0060079 | excitatory postsynaptic potential | 26/2872 | 70/13036 | 1.69 | 0.002844408 | 0.014474608 | BP |
| GO:0043281 | regulation of cysteine-type endopeptidase activity involved in apoptotic process | 57/2872 | 184/13036 | 1.41 | 0.002864683 | 0.014529535 | BP |
| GO:0046883 | regulation of hormone secretion | 57/2872 | 184/13036 | 1.41 | 0.002864683 | 0.014529535 | BP |
| GO:0008585 | female gonad development | 28/2872 | 77/13036 | 1.65 | 0.002865711 | 0.014529535 | BP |
| GO:0051961 | negative regulation of nervous system development | 72/2872 | 242/13036 | 1.35 | 0.002871168 | 0.014539429 | BP |
| GO:0050728 | negative regulation of inflammatory response | 35/2872 | 102/13036 | 1.56 | 0.002900487 | 0.014669984 | BP |
| GO:0046578 | regulation of Ras protein signal transduction | 53/2872 | 169/13036 | 1.42 | 0.00295818 | 0.014943561 | BP |
| GO:0010878 | cholesterol storage | 9/2872 | 16/13036 | 2.55 | 0.003001332 | 0.01503337 | BP |
| GO:0010893 | positive regulation of steroid biosynthetic process | 9/2872 | 16/13036 | 2.55 | 0.003001332 | 0.01503337 | BP |
| GO:0034368 | protein-lipid complex remodeling | 9/2872 | 16/13036 | 2.55 | 0.003001332 | 0.01503337 | BP |
| GO:0034369 | plasma lipoprotein particle remodeling | 9/2872 | 16/13036 | 2.55 | 0.003001332 | 0.01503337 | BP |
| GO:0040037 | negative regulation of fibroblast growth factor receptor signaling pathway | 9/2872 | 16/13036 | 2.55 | 0.003001332 | 0.01503337 | BP |
| GO:0098581 | detection of external biotic stimulus | 9/2872 | 16/13036 | 2.55 | 0.003001332 | 0.01503337 | BP |
| GO:1904706 | negative regulation of vascular associated smooth muscle cell proliferation | 9/2872 | 16/13036 | 2.55 | 0.003001332 | 0.01503337 | BP |
| GO:0031644 | regulation of nervous system process | 33/2872 | 95/13036 | 1.58 | 0.003016458 | 0.01509091 | BP |
| GO:0042475 | odontogenesis of dentin-containing tooth | 23/2872 | 60/13036 | 1.74 | 0.00305635 | 0.015272061 | BP |
| GO:0034754 | cellular hormone metabolic process | 31/2872 | 88/13036 | 1.6 | 0.003110713 | 0.015524997 | BP |
| GO:0032835 | glomerulus development | 20/2872 | 50/13036 | 1.82 | 0.003132606 | 0.015615469 | BP |
| GO:0006040 | amino sugar metabolic process | 16/2872 | 37/13036 | 1.96 | 0.003155258 | 0.015705045 | BP |
| GO:0014032 | neural crest cell development | 25/2872 | 67/13036 | 1.69 | 0.003161936 | 0.015705045 | BP |
| GO:0019915 | lipid storage | 25/2872 | 67/13036 | 1.69 | 0.003161936 | 0.015705045 | BP |
| GO:0001893 | maternal placenta development | 15/2872 | 34/13036 | 2 | 0.003334218 | 0.016521182 | BP |
| GO:0010863 | positive regulation of phospholipase C activity | 15/2872 | 34/13036 | 2 | 0.003334218 | 0.016521182 | BP |
| GO:2000377 | regulation of reactive oxygen species metabolic process | 48/2872 | 151/13036 | 1.44 | 0.003369612 | 0.016564077 | BP |
| GO:0001759 | organ induction | 10/2872 | 19/13036 | 2.39 | 0.003374826 | 0.016564077 | BP |
| GO:0002335 | mature B cell differentiation | 10/2872 | 19/13036 | 2.39 | 0.003374826 | 0.016564077 | BP |
| GO:0010669 | epithelial structure maintenance | 10/2872 | 19/13036 | 2.39 | 0.003374826 | 0.016564077 | BP |
| GO:0014046 | dopamine secretion | 10/2872 | 19/13036 | 2.39 | 0.003374826 | 0.016564077 | BP |
| GO:0014059 | regulation of dopamine secretion | 10/2872 | 19/13036 | 2.39 | 0.003374826 | 0.016564077 | BP |
| GO:0045987 | positive regulation of smooth muscle contraction | 10/2872 | 19/13036 | 2.39 | 0.003374826 | 0.016564077 | BP |
| GO:0060571 | morphogenesis of an epithelial fold | 10/2872 | 19/13036 | 2.39 | 0.003374826 | 0.016564077 | BP |
| GO:1903039 | positive regulation of leukocyte cell-cell adhesion | 53/2872 | 170/13036 | 1.42 | 0.00339533 | 0.016645015 | BP |
| GO:0072678 | T cell migration | 19/2872 | 47/13036 | 1.83 | 0.003420493 | 0.016748577 | BP |
| GO:0051897 | positive regulation of protein kinase B signaling | 41/2872 | 125/13036 | 1.49 | 0.003459264 | 0.016905424 | BP |
| GO:0000302 | response to reactive oxygen species | 60/2872 | 197/13036 | 1.38 | 0.003470817 | 0.016905424 | BP |
| GO:0043271 | negative regulation of ion transport | 35/2872 | 103/13036 | 1.54 | 0.003479731 | 0.016905424 | BP |
| GO:0002548 | monocyte chemotaxis | 14/2872 | 31/13036 | 2.05 | 0.003485135 | 0.016905424 | BP |
| GO:0045601 | regulation of endothelial cell differentiation | 14/2872 | 31/13036 | 2.05 | 0.003485135 | 0.016905424 | BP |
| GO:0045684 | positive regulation of epidermis development | 14/2872 | 31/13036 | 2.05 | 0.003485135 | 0.016905424 | BP |
| GO:0048663 | neuron fate commitment | 14/2872 | 31/13036 | 2.05 | 0.003485135 | 0.016905424 | BP |
| GO:0089718 | amino acid import across plasma membrane | 14/2872 | 31/13036 | 2.05 | 0.003485135 | 0.016905424 | BP |
| GO:0001523 | retinoid metabolic process | 24/2872 | 64/13036 | 1.7 | 0.003512984 | 0.017020607 | BP |
| GO:0032352 | positive regulation of hormone metabolic process | 7/2872 | 11/13036 | 2.89 | 0.003535513 | 0.017069934 | BP |
| GO:0045741 | positive regulation of epidermal growth factor-activated receptor activity | 7/2872 | 11/13036 | 2.89 | 0.003535513 | 0.017069934 | BP |
| GO:0072537 | fibroblast activation | 7/2872 | 11/13036 | 2.89 | 0.003535513 | 0.017069934 | BP |
| GO:0060419 | heart growth | 28/2872 | 78/13036 | 1.63 | 0.003547216 | 0.017106523 | BP |
| GO:0009791 | post-embryonic development | 26/2872 | 71/13036 | 1.66 | 0.003563337 | 0.017118717 | BP |
| GO:0070168 | negative regulation of biomineral tissue development | 11/2872 | 22/13036 | 2.27 | 0.003569553 | 0.017118717 | BP |
| GO:0110150 | negative regulation of biomineralization | 11/2872 | 22/13036 | 2.27 | 0.003569553 | 0.017118717 | BP |
| GO:0003156 | regulation of animal organ formation | 13/2872 | 28/13036 | 2.11 | 0.00359102 | 0.017118717 | BP |
| GO:0015872 | dopamine transport | 13/2872 | 28/13036 | 2.11 | 0.00359102 | 0.017118717 | BP |
| GO:0019359 | nicotinamide nucleotide biosynthetic process | 13/2872 | 28/13036 | 2.11 | 0.00359102 | 0.017118717 | BP |
| GO:0019363 | pyridine nucleotide biosynthetic process | 13/2872 | 28/13036 | 2.11 | 0.00359102 | 0.017118717 | BP |
| GO:0042908 | xenobiotic transport | 13/2872 | 28/13036 | 2.11 | 0.00359102 | 0.017118717 | BP |
| GO:0060603 | mammary gland duct morphogenesis | 13/2872 | 28/13036 | 2.11 | 0.00359102 | 0.017118717 | BP |
| GO:0110111 | negative regulation of animal organ morphogenesis | 13/2872 | 28/13036 | 2.11 | 0.00359102 | 0.017118717 | BP |
| GO:0033198 | response to ATP | 12/2872 | 25/13036 | 2.18 | 0.003629101 | 0.017240803 | BP |
| GO:0060441 | epithelial tube branching involved in lung morphogenesis | 12/2872 | 25/13036 | 2.18 | 0.003629101 | 0.017240803 | BP |
| GO:0070232 | regulation of T cell apoptotic process | 12/2872 | 25/13036 | 2.18 | 0.003629101 | 0.017240803 | BP |
| GO:0030038 | contractile actin filament bundle assembly | 33/2872 | 96/13036 | 1.56 | 0.003642374 | 0.017264303 | BP |
| GO:0043149 | stress fiber assembly | 33/2872 | 96/13036 | 1.56 | 0.003642374 | 0.017264303 | BP |
| GO:0046879 | hormone secretion | 65/2872 | 217/13036 | 1.36 | 0.00374803 | 0.017744819 | BP |
| GO:0007613 | memory | 31/2872 | 89/13036 | 1.58 | 0.003784495 | 0.017897031 | BP |
| GO:0002697 | regulation of immune effector process | 82/2872 | 284/13036 | 1.31 | 0.003816346 | 0.018027097 | BP |
| GO:0090101 | negative regulation of transmembrane receptor protein serine/threonine kinase signaling pathway | 34/2872 | 100/13036 | 1.54 | 0.003901433 | 0.018408054 | BP |
| GO:0070374 | positive regulation of ERK1 and ERK2 cascade | 44/2872 | 137/13036 | 1.46 | 0.003929657 | 0.018520153 | BP |
| GO:0002286 | T cell activation involved in immune response | 25/2872 | 68/13036 | 1.67 | 0.00397208 | 0.01867764 | BP |
| GO:0043502 | regulation of muscle adaptation | 25/2872 | 68/13036 | 1.67 | 0.00397208 | 0.01867764 | BP |
| GO:0050870 | positive regulation of T cell activation | 49/2872 | 156/13036 | 1.43 | 0.004011406 | 0.018841197 | BP |
| GO:0046850 | regulation of bone remodeling | 17/2872 | 41/13036 | 1.88 | 0.004034778 | 0.018929539 | BP |
| GO:0050670 | regulation of lymphocyte proliferation | 45/2872 | 141/13036 | 1.45 | 0.004076306 | 0.019102759 | BP |
| GO:0043900 | regulation of multi-organism process | 20/2872 | 51/13036 | 1.78 | 0.00410342 | 0.019208119 | BP |
| GO:0031348 | negative regulation of defense response | 50/2872 | 160/13036 | 1.42 | 0.004125295 | 0.019267025 | BP |
| GO:0050770 | regulation of axonogenesis | 50/2872 | 160/13036 | 1.42 | 0.004125295 | 0.019267025 | BP |
| GO:0050777 | negative regulation of immune response | 35/2872 | 104/13036 | 1.53 | 0.004154726 | 0.019382653 | BP |
| GO:0043500 | muscle adaptation | 30/2872 | 86/13036 | 1.58 | 0.004240095 | 0.019758694 | BP |
| GO:0032535 | regulation of cellular component size | 93/2872 | 329/13036 | 1.28 | 0.004249261 | 0.019779183 | BP |
| GO:0035249 | synaptic transmission, glutamatergic | 22/2872 | 58/13036 | 1.72 | 0.004327386 | 0.020075237 | BP |
| GO:0045844 | positive regulation of striated muscle tissue development | 22/2872 | 58/13036 | 1.72 | 0.004327386 | 0.020075237 | BP |
| GO:0048636 | positive regulation of muscle organ development | 22/2872 | 58/13036 | 1.72 | 0.004327386 | 0.020075237 | BP |
| GO:0060043 | regulation of cardiac muscle cell proliferation | 16/2872 | 38/13036 | 1.91 | 0.004351298 | 0.020152095 | BP |
| GO:0030324 | lung development | 47/2872 | 149/13036 | 1.43 | 0.004353671 | 0.020152095 | BP |
| GO:1905952 | regulation of lipid localization | 36/2872 | 108/13036 | 1.51 | 0.004401522 | 0.020350872 | BP |
| GO:0019233 | sensory perception of pain | 24/2872 | 65/13036 | 1.68 | 0.004426389 | 0.020434226 | BP |
| GO:0043154 | negative regulation of cysteine-type endopeptidase activity involved in apoptotic process | 26/2872 | 72/13036 | 1.64 | 0.004429404 | 0.020434226 | BP |
| GO:0021537 | telencephalon development | 60/2872 | 199/13036 | 1.37 | 0.004444287 | 0.020480107 | BP |
| GO:0010632 | regulation of epithelial cell migration | 61/2872 | 203/13036 | 1.36 | 0.004508648 | 0.02064146 | BP |
| GO:0046580 | negative regulation of Ras protein signal transduction | 19/2872 | 48/13036 | 1.8 | 0.004510892 | 0.02064146 | BP |
| GO:0010885 | regulation of cholesterol storage | 8/2872 | 14/13036 | 2.59 | 0.004539026 | 0.02064146 | BP |
| GO:0015874 | norepinephrine transport | 8/2872 | 14/13036 | 2.59 | 0.004539026 | 0.02064146 | BP |
| GO:0021783 | preganglionic parasympathetic fiber development | 8/2872 | 14/13036 | 2.59 | 0.004539026 | 0.02064146 | BP |
| GO:0030502 | negative regulation of bone mineralization | 8/2872 | 14/13036 | 2.59 | 0.004539026 | 0.02064146 | BP |
| GO:0032616 | interleukin-13 production | 8/2872 | 14/13036 | 2.59 | 0.004539026 | 0.02064146 | BP |
| GO:0035812 | renal sodium excretion | 8/2872 | 14/13036 | 2.59 | 0.004539026 | 0.02064146 | BP |
| GO:0050961 | detection of temperature stimulus involved in sensory perception | 8/2872 | 14/13036 | 2.59 | 0.004539026 | 0.02064146 | BP |
| GO:0090036 | regulation of protein kinase C signaling | 8/2872 | 14/13036 | 2.59 | 0.004539026 | 0.02064146 | BP |
| GO:0090136 | epithelial cell-cell adhesion | 8/2872 | 14/13036 | 2.59 | 0.004539026 | 0.02064146 | BP |
| GO:0098743 | cell aggregation | 8/2872 | 14/13036 | 2.59 | 0.004539026 | 0.02064146 | BP |
| GO:0007626 | locomotory behavior | 44/2872 | 138/13036 | 1.45 | 0.004563171 | 0.020728536 | BP |
| GO:0048640 | negative regulation of developmental growth | 31/2872 | 90/13036 | 1.56 | 0.00457785 | 0.020772464 | BP |
| GO:0010769 | regulation of cell morphogenesis involved in differentiation | 77/2872 | 266/13036 | 1.31 | 0.004605373 | 0.020874513 | BP |
| GO:1901343 | negative regulation of vasculature development | 37/2872 | 112/13036 | 1.5 | 0.004641233 | 0.021014085 | BP |
| GO:0002279 | mast cell activation involved in immune response | 15/2872 | 35/13036 | 1.95 | 0.00466299 | 0.021043677 | BP |
| GO:0043303 | mast cell degranulation | 15/2872 | 35/13036 | 1.95 | 0.00466299 | 0.021043677 | BP |
| GO:0043370 | regulation of CD4-positive, alpha-beta T cell differentiation | 15/2872 | 35/13036 | 1.95 | 0.00466299 | 0.021043677 | BP |
| GO:0050768 | negative regulation of neurogenesis | 66/2872 | 223/13036 | 1.34 | 0.004777962 | 0.021539096 | BP |
| GO:0010517 | regulation of phospholipase activity | 21/2872 | 55/13036 | 1.73 | 0.004796604 | 0.021599656 | BP |
| GO:0042303 | molting cycle | 27/2872 | 76/13036 | 1.61 | 0.004881464 | 0.02191042 | BP |
| GO:0042633 | hair cycle | 27/2872 | 76/13036 | 1.61 | 0.004881464 | 0.02191042 | BP |
| GO:0097306 | cellular response to alcohol | 27/2872 | 76/13036 | 1.61 | 0.004881464 | 0.02191042 | BP |
| GO:0021953 | central nervous system neuron differentiation | 42/2872 | 131/13036 | 1.46 | 0.004928908 | 0.022080258 | BP |
| GO:0007492 | endoderm development | 23/2872 | 62/13036 | 1.68 | 0.004930877 | 0.022080258 | BP |
| GO:0001755 | neural crest cell migration | 18/2872 | 45/13036 | 1.82 | 0.004946956 | 0.022080258 | BP |
| GO:0007566 | embryo implantation | 18/2872 | 45/13036 | 1.82 | 0.004946956 | 0.022080258 | BP |
| GO:0048864 | stem cell development | 25/2872 | 69/13036 | 1.64 | 0.004949247 | 0.022080258 | BP |
| GO:0001974 | blood vessel remodeling | 14/2872 | 32/13036 | 1.99 | 0.00495657 | 0.022080258 | BP |
| GO:0048048 | embryonic eye morphogenesis | 14/2872 | 32/13036 | 1.99 | 0.00495657 | 0.022080258 | BP |
| GO:0045807 | positive regulation of endocytosis | 30/2872 | 87/13036 | 1.57 | 0.005133935 | 0.022776067 | BP |
| GO:0010818 | T cell chemotaxis | 9/2872 | 17/13036 | 2.4 | 0.005140223 | 0.022776067 | BP |
| GO:0034367 | protein-containing complex remodeling | 9/2872 | 17/13036 | 2.4 | 0.005140223 | 0.022776067 | BP |
| GO:0042044 | fluid transport | 9/2872 | 17/13036 | 2.4 | 0.005140223 | 0.022776067 | BP |
| GO:0071294 | cellular response to zinc ion | 9/2872 | 17/13036 | 2.4 | 0.005140223 | 0.022776067 | BP |
| GO:0045933 | positive regulation of muscle contraction | 13/2872 | 29/13036 | 2.03 | 0.005212919 | 0.023048931 | BP |
| GO:1902742 | apoptotic process involved in development | 13/2872 | 29/13036 | 2.03 | 0.005212919 | 0.023048931 | BP |
| GO:0032675 | regulation of interleukin-6 production | 33/2872 | 98/13036 | 1.53 | 0.005228981 | 0.023070756 | BP |
| GO:0048593 | camera-type eye morphogenesis | 33/2872 | 98/13036 | 1.53 | 0.005228981 | 0.023070756 | BP |
| GO:0060193 | positive regulation of lipase activity | 20/2872 | 52/13036 | 1.75 | 0.005310823 | 0.023370184 | BP |
| GO:0046661 | male sex differentiation | 40/2872 | 124/13036 | 1.46 | 0.005313751 | 0.023370184 | BP |
| GO:0051048 | negative regulation of secretion | 40/2872 | 124/13036 | 1.46 | 0.005313751 | 0.023370184 | BP |
| GO:0001933 | negative regulation of protein phosphorylation | 104/2872 | 376/13036 | 1.26 | 0.005355337 | 0.023528131 | BP |
| GO:0018146 | keratan sulfate biosynthetic process | 12/2872 | 26/13036 | 2.09 | 0.005404916 | 0.023579751 | BP |
| GO:0035886 | vascular associated smooth muscle cell differentiation | 12/2872 | 26/13036 | 2.09 | 0.005404916 | 0.023579751 | BP |
| GO:0040036 | regulation of fibroblast growth factor receptor signaling pathway | 12/2872 | 26/13036 | 2.09 | 0.005404916 | 0.023579751 | BP |
| GO:0060351 | cartilage development involved in endochondral bone morphogenesis | 12/2872 | 26/13036 | 2.09 | 0.005404916 | 0.023579751 | BP |
| GO:0140353 | lipid export from cell | 12/2872 | 26/13036 | 2.09 | 0.005404916 | 0.023579751 | BP |
| GO:1903307 | positive regulation of regulated secretory pathway | 17/2872 | 42/13036 | 1.84 | 0.005409243 | 0.023579751 | BP |
| GO:0021772 | olfactory bulb development | 10/2872 | 20/13036 | 2.27 | 0.005429627 | 0.023579751 | BP |
| GO:0045907 | positive regulation of vasoconstriction | 10/2872 | 20/13036 | 2.27 | 0.005429627 | 0.023579751 | BP |
| GO:0055090 | acylglycerol homeostasis | 10/2872 | 20/13036 | 2.27 | 0.005429627 | 0.023579751 | BP |
| GO:0070328 | triglyceride homeostasis | 10/2872 | 20/13036 | 2.27 | 0.005429627 | 0.023579751 | BP |
| GO:0071280 | cellular response to copper ion | 10/2872 | 20/13036 | 2.27 | 0.005429627 | 0.023579751 | BP |
| GO:0006970 | response to osmotic stress | 26/2872 | 73/13036 | 1.62 | 0.005464884 | 0.023639047 | BP |
| GO:0014033 | neural crest cell differentiation | 26/2872 | 73/13036 | 1.62 | 0.005464884 | 0.023639047 | BP |
| GO:0060420 | regulation of heart growth | 22/2872 | 59/13036 | 1.69 | 0.005490444 | 0.023639047 | BP |
| GO:0008045 | motor neuron axon guidance | 11/2872 | 23/13036 | 2.17 | 0.005494579 | 0.023639047 | BP |
| GO:0032941 | secretion by tissue | 11/2872 | 23/13036 | 2.17 | 0.005494579 | 0.023639047 | BP |
| GO:0051385 | response to mineralocorticoid | 11/2872 | 23/13036 | 2.17 | 0.005494579 | 0.023639047 | BP |
| GO:0060055 | angiogenesis involved in wound healing | 11/2872 | 23/13036 | 2.17 | 0.005494579 | 0.023639047 | BP |
| GO:0060292 | long-term synaptic depression | 11/2872 | 23/13036 | 2.17 | 0.005494579 | 0.023639047 | BP |
| GO:1901071 | glucosamine-containing compound metabolic process | 11/2872 | 23/13036 | 2.17 | 0.005494579 | 0.023639047 | BP |
| GO:0002526 | acute inflammatory response | 24/2872 | 66/13036 | 1.65 | 0.005529613 | 0.02376512 | BP |
| GO:0071407 | cellular response to organic cyclic compound | 122/2872 | 450/13036 | 1.23 | 0.005589689 | 0.023998447 | BP |
| GO:0071241 | cellular response to inorganic substance | 52/2872 | 170/13036 | 1.39 | 0.005639129 | 0.024185672 | BP |
| GO:0032956 | regulation of actin cytoskeleton organization | 87/2872 | 308/13036 | 1.28 | 0.005668337 | 0.02428583 | BP |
| GO:0032635 | interleukin-6 production | 35/2872 | 106/13036 | 1.5 | 0.0058413 | 0.025001058 | BP |
| GO:0043030 | regulation of macrophage activation | 16/2872 | 39/13036 | 1.86 | 0.00589317 | 0.025171111 | BP |
| GO:0060443 | mammary gland morphogenesis | 16/2872 | 39/13036 | 1.86 | 0.00589317 | 0.025171111 | BP |
| GO:0007219 | Notch signaling pathway | 48/2872 | 155/13036 | 1.41 | 0.005899826 | 0.025173613 | BP |
| GO:0031668 | cellular response to extracellular stimulus | 65/2872 | 221/13036 | 1.33 | 0.005927421 | 0.025265365 | BP |
| GO:0006897 | endocytosis | 124/2872 | 459/13036 | 1.23 | 0.005935914 | 0.025275588 | BP |
| GO:0050803 | regulation of synapse structure or activity | 55/2872 | 182/13036 | 1.37 | 0.005943031 | 0.025279936 | BP |
| GO:0033138 | positive regulation of peptidyl-serine phosphorylation | 27/2872 | 77/13036 | 1.59 | 0.00596997 | 0.025368509 | BP |
| GO:0035282 | segmentation | 25/2872 | 70/13036 | 1.62 | 0.006118603 | 0.02597349 | BP |
| GO:2000177 | regulation of neural precursor cell proliferation | 23/2872 | 63/13036 | 1.66 | 0.006177277 | 0.026195749 | BP |
| GO:2000116 | regulation of cysteine-type endopeptidase activity | 59/2872 | 198/13036 | 1.35 | 0.006271258 | 0.026567125 | BP |
| GO:0003197 | endocardial cushion development | 15/2872 | 36/13036 | 1.89 | 0.006390944 | 0.026909236 | BP |
| GO:0006911 | phagocytosis, engulfment | 15/2872 | 36/13036 | 1.89 | 0.006390944 | 0.026909236 | BP |
| GO:0014075 | response to amine | 15/2872 | 36/13036 | 1.89 | 0.006390944 | 0.026909236 | BP |
| GO:0031670 | cellular response to nutrient | 15/2872 | 36/13036 | 1.89 | 0.006390944 | 0.026909236 | BP |
| GO:0043277 | apoptotic cell clearance | 15/2872 | 36/13036 | 1.89 | 0.006390944 | 0.026909236 | BP |
| GO:1900274 | regulation of phospholipase C activity | 15/2872 | 36/13036 | 1.89 | 0.006390944 | 0.026909236 | BP |
| GO:0045639 | positive regulation of myeloid cell differentiation | 28/2872 | 81/13036 | 1.57 | 0.00646189 | 0.027152826 | BP |
| GO:0050772 | positive regulation of axonogenesis | 28/2872 | 81/13036 | 1.57 | 0.00646189 | 0.027152826 | BP |
| GO:0010518 | positive regulation of phospholipase activity | 18/2872 | 46/13036 | 1.78 | 0.006482507 | 0.027184375 | BP |
| GO:2000401 | regulation of lymphocyte migration | 18/2872 | 46/13036 | 1.78 | 0.006482507 | 0.027184375 | BP |
| GO:0051251 | positive regulation of lymphocyte activation | 63/2872 | 214/13036 | 1.34 | 0.006519689 | 0.02731268 | BP |
| GO:0002700 | regulation of production of molecular mediator of immune response | 34/2872 | 103/13036 | 1.5 | 0.006555737 | 0.027435979 | BP |
| GO:0001659 | temperature homeostasis | 42/2872 | 133/13036 | 1.43 | 0.00661755 | 0.027666753 | BP |
| GO:0006027 | glycosaminoglycan catabolic process | 20/2872 | 53/13036 | 1.71 | 0.006795488 | 0.02815356 | BP |
| GO:0035924 | cellular response to vascular endothelial growth factor stimulus | 20/2872 | 53/13036 | 1.71 | 0.006795488 | 0.02815356 | BP |
| GO:0051148 | negative regulation of muscle cell differentiation | 20/2872 | 53/13036 | 1.71 | 0.006795488 | 0.02815356 | BP |
| GO:0014841 | skeletal muscle satellite cell proliferation | 7/2872 | 12/13036 | 2.65 | 0.006878373 | 0.02815356 | BP |
| GO:0014856 | skeletal muscle cell proliferation | 7/2872 | 12/13036 | 2.65 | 0.006878373 | 0.02815356 | BP |
| GO:0032634 | interleukin-5 production | 7/2872 | 12/13036 | 2.65 | 0.006878373 | 0.02815356 | BP |
| GO:0032656 | regulation of interleukin-13 production | 7/2872 | 12/13036 | 2.65 | 0.006878373 | 0.02815356 | BP |
| GO:0032674 | regulation of interleukin-5 production | 7/2872 | 12/13036 | 2.65 | 0.006878373 | 0.02815356 | BP |
| GO:0034375 | high-density lipoprotein particle remodeling | 7/2872 | 12/13036 | 2.65 | 0.006878373 | 0.02815356 | BP |
| GO:0042362 | fat-soluble vitamin biosynthetic process | 7/2872 | 12/13036 | 2.65 | 0.006878373 | 0.02815356 | BP |
| GO:0043374 | CD8-positive, alpha-beta T cell differentiation | 7/2872 | 12/13036 | 2.65 | 0.006878373 | 0.02815356 | BP |
| GO:0048665 | neuron fate specification | 7/2872 | 12/13036 | 2.65 | 0.006878373 | 0.02815356 | BP |
| GO:0050965 | detection of temperature stimulus involved in sensory perception of pain | 7/2872 | 12/13036 | 2.65 | 0.006878373 | 0.02815356 | BP |
| GO:0050966 | detection of mechanical stimulus involved in sensory perception of pain | 7/2872 | 12/13036 | 2.65 | 0.006878373 | 0.02815356 | BP |
| GO:0051386 | regulation of neurotrophin TRK receptor signaling pathway | 7/2872 | 12/13036 | 2.65 | 0.006878373 | 0.02815356 | BP |
| GO:0061687 | detoxification of inorganic compound | 7/2872 | 12/13036 | 2.65 | 0.006878373 | 0.02815356 | BP |
| GO:0070486 | leukocyte aggregation | 7/2872 | 12/13036 | 2.65 | 0.006878373 | 0.02815356 | BP |
| GO:0097501 | stress response to metal ion | 7/2872 | 12/13036 | 2.65 | 0.006878373 | 0.02815356 | BP |
| GO:1905939 | regulation of gonad development | 7/2872 | 12/13036 | 2.65 | 0.006878373 | 0.02815356 | BP |
| GO:0021879 | forebrain neuron differentiation | 14/2872 | 33/13036 | 1.93 | 0.006890119 | 0.02815356 | BP |
| GO:0046456 | icosanoid biosynthetic process | 14/2872 | 33/13036 | 1.93 | 0.006890119 | 0.02815356 | BP |
| GO:0048846 | axon extension involved in axon guidance | 14/2872 | 33/13036 | 1.93 | 0.006890119 | 0.02815356 | BP |
| GO:1902284 | neuron projection extension involved in neuron projection guidance | 14/2872 | 33/13036 | 1.93 | 0.006890119 | 0.02815356 | BP |
| GO:0001764 | neuron migration | 39/2872 | 122/13036 | 1.45 | 0.006932795 | 0.028296043 | BP |
| GO:0002065 | columnar/cuboidal epithelial cell differentiation | 29/2872 | 85/13036 | 1.55 | 0.006938635 | 0.028296043 | BP |
| GO:0051896 | regulation of protein kinase B signaling | 52/2872 | 172/13036 | 1.37 | 0.007256522 | 0.029563331 | BP |
| GO:0048806 | genitalia development | 13/2872 | 30/13036 | 1.97 | 0.00737154 | 0.030002445 | BP |
| GO:0097305 | response to alcohol | 54/2872 | 180/13036 | 1.36 | 0.007470766 | 0.030376488 | BP |
| GO:0030323 | respiratory tube development | 47/2872 | 153/13036 | 1.39 | 0.00752392 | 0.030562651 | BP |
| GO:0050805 | negative regulation of synaptic transmission | 19/2872 | 50/13036 | 1.72 | 0.00755146 | 0.030644506 | BP |
| GO:0002028 | regulation of sodium ion transport | 23/2872 | 64/13036 | 1.63 | 0.00767188 | 0.031062588 | BP |
| GO:0045682 | regulation of epidermis development | 23/2872 | 64/13036 | 1.63 | 0.00767188 | 0.031062588 | BP |
| GO:0099504 | synaptic vesicle cycle | 48/2872 | 157/13036 | 1.39 | 0.007676953 | 0.031062588 | BP |
| GO:0001958 | endochondral ossification | 12/2872 | 27/13036 | 2.02 | 0.007806338 | 0.031326881 | BP |
| GO:0036075 | replacement ossification | 12/2872 | 27/13036 | 2.02 | 0.007806338 | 0.031326881 | BP |
| GO:1990573 | potassium ion import across plasma membrane | 12/2872 | 27/13036 | 2.02 | 0.007806338 | 0.031326881 | BP |
| GO:0002761 | regulation of myeloid leukocyte differentiation | 31/2872 | 93/13036 | 1.51 | 0.007841001 | 0.031326881 | BP |
| GO:0030534 | adult behavior | 31/2872 | 93/13036 | 1.51 | 0.007841001 | 0.031326881 | BP |
| GO:0050795 | regulation of behavior | 16/2872 | 40/13036 | 1.82 | 0.007848138 | 0.031326881 | BP |
| GO:0140115 | export across plasma membrane | 16/2872 | 40/13036 | 1.82 | 0.007848138 | 0.031326881 | BP |
| GO:0007288 | sperm axoneme assembly | 8/2872 | 15/13036 | 2.42 | 0.007863127 | 0.031326881 | BP |
| GO:0031639 | plasminogen activation | 8/2872 | 15/13036 | 2.42 | 0.007863127 | 0.031326881 | BP |
| GO:0035994 | response to muscle stretch | 8/2872 | 15/13036 | 2.42 | 0.007863127 | 0.031326881 | BP |
| GO:0042481 | regulation of odontogenesis | 8/2872 | 15/13036 | 2.42 | 0.007863127 | 0.031326881 | BP |
| GO:0048791 | calcium ion-regulated exocytosis of neurotransmitter | 8/2872 | 15/13036 | 2.42 | 0.007863127 | 0.031326881 | BP |
| GO:0060080 | inhibitory postsynaptic potential | 8/2872 | 15/13036 | 2.42 | 0.007863127 | 0.031326881 | BP |
| GO:0060644 | mammary gland epithelial cell differentiation | 8/2872 | 15/13036 | 2.42 | 0.007863127 | 0.031326881 | BP |
| GO:0070233 | negative regulation of T cell apoptotic process | 8/2872 | 15/13036 | 2.42 | 0.007863127 | 0.031326881 | BP |
| GO:1903019 | negative regulation of glycoprotein metabolic process | 8/2872 | 15/13036 | 2.42 | 0.007863127 | 0.031326881 | BP |
| GO:0032355 | response to estradiol | 35/2872 | 108/13036 | 1.47 | 0.008068111 | 0.032112697 | BP |
| GO:0043523 | regulation of neuron apoptotic process | 51/2872 | 169/13036 | 1.37 | 0.008083282 | 0.032142234 | BP |
| GO:0043171 | peptide catabolic process | 11/2872 | 24/13036 | 2.08 | 0.008151601 | 0.032351857 | BP |
| GO:0070229 | negative regulation of lymphocyte apoptotic process | 11/2872 | 24/13036 | 2.08 | 0.008151601 | 0.032351857 | BP |
| GO:0002360 | T cell lineage commitment | 9/2872 | 18/13036 | 2.27 | 0.008291888 | 0.032684229 | BP |
| GO:0030728 | ovulation | 9/2872 | 18/13036 | 2.27 | 0.008291888 | 0.032684229 | BP |
| GO:0033561 | regulation of water loss via skin | 9/2872 | 18/13036 | 2.27 | 0.008291888 | 0.032684229 | BP |
| GO:0001990 | regulation of systemic arterial blood pressure by hormone | 10/2872 | 21/13036 | 2.16 | 0.008344145 | 0.032684229 | BP |
| GO:0006706 | steroid catabolic process | 10/2872 | 21/13036 | 2.16 | 0.008344145 | 0.032684229 | BP |
| GO:0021522 | spinal cord motor neuron differentiation | 10/2872 | 21/13036 | 2.16 | 0.008344145 | 0.032684229 | BP |
| GO:0034698 | response to gonadotropin | 10/2872 | 21/13036 | 2.16 | 0.008344145 | 0.032684229 | BP |
| GO:0036037 | CD8-positive, alpha-beta T cell activation | 10/2872 | 21/13036 | 2.16 | 0.008344145 | 0.032684229 | BP |
| GO:0046717 | acid secretion | 10/2872 | 21/13036 | 2.16 | 0.008344145 | 0.032684229 | BP |
| GO:0048745 | smooth muscle tissue development | 10/2872 | 21/13036 | 2.16 | 0.008344145 | 0.032684229 | BP |
| GO:0060445 | branching involved in salivary gland morphogenesis | 10/2872 | 21/13036 | 2.16 | 0.008344145 | 0.032684229 | BP |
| GO:0070633 | transepithelial transport | 10/2872 | 21/13036 | 2.16 | 0.008344145 | 0.032684229 | BP |
| GO:1903010 | regulation of bone development | 10/2872 | 21/13036 | 2.16 | 0.008344145 | 0.032684229 | BP |
| GO:0022412 | cellular process involved in reproduction in multicellular organism | 71/2872 | 248/13036 | 1.3 | 0.008345677 | 0.032684229 | BP |
| GO:0001954 | positive regulation of cell-matrix adhesion | 18/2872 | 47/13036 | 1.74 | 0.008383068 | 0.032707123 | BP |
| GO:0032768 | regulation of monooxygenase activity | 18/2872 | 47/13036 | 1.74 | 0.008383068 | 0.032707123 | BP |
| GO:0046637 | regulation of alpha-beta T cell differentiation | 18/2872 | 47/13036 | 1.74 | 0.008383068 | 0.032707123 | BP |
| GO:0050891 | multicellular organismal water homeostasis | 18/2872 | 47/13036 | 1.74 | 0.008383068 | 0.032707123 | BP |
| GO:0007292 | female gamete generation | 36/2872 | 112/13036 | 1.46 | 0.008398071 | 0.032734862 | BP |
| GO:0072524 | pyridine-containing compound metabolic process | 15/2872 | 37/13036 | 1.84 | 0.008597093 | 0.033464612 | BP |
| GO:0045685 | regulation of glial cell differentiation | 20/2872 | 54/13036 | 1.68 | 0.008601425 | 0.033464612 | BP |
| GO:0031346 | positive regulation of cell projection organization | 93/2872 | 337/13036 | 1.25 | 0.008661431 | 0.03366649 | BP |
| GO:0030307 | positive regulation of cell growth | 43/2872 | 139/13036 | 1.4 | 0.008984338 | 0.034888914 | BP |
| GO:0006633 | fatty acid biosynthetic process | 38/2872 | 120/13036 | 1.44 | 0.00900949 | 0.034953856 | BP |
| GO:0098739 | import across plasma membrane | 34/2872 | 105/13036 | 1.47 | 0.009055302 | 0.035098762 | BP |
| GO:0042326 | negative regulation of phosphorylation | 109/2872 | 403/13036 | 1.23 | 0.009109256 | 0.03527492 | BP |
| GO:0014897 | striated muscle hypertrophy | 25/2872 | 72/13036 | 1.58 | 0.009144531 | 0.035345517 | BP |
| GO:0032609 | interferon-gamma production | 25/2872 | 72/13036 | 1.58 | 0.009144531 | 0.035345517 | BP |
| GO:0009615 | response to virus | 76/2872 | 269/13036 | 1.28 | 0.009197643 | 0.035517704 | BP |
| GO:0099003 | vesicle-mediated transport in synapse | 52/2872 | 174/13036 | 1.36 | 0.009248639 | 0.035681407 | BP |
| GO:0035272 | exocrine system development | 17/2872 | 44/13036 | 1.75 | 0.009294524 | 0.035758643 | BP |
| GO:0060976 | coronary vasculature development | 17/2872 | 44/13036 | 1.75 | 0.009294524 | 0.035758643 | BP |
| GO:2000514 | regulation of CD4-positive, alpha-beta T cell activation | 17/2872 | 44/13036 | 1.75 | 0.009294524 | 0.035758643 | BP |
| GO:0003254 | regulation of membrane depolarization | 14/2872 | 34/13036 | 1.87 | 0.009379251 | 0.035952007 | BP |
| GO:0035850 | epithelial cell differentiation involved in kidney development | 14/2872 | 34/13036 | 1.87 | 0.009379251 | 0.035952007 | BP |
| GO:2000826 | regulation of heart morphogenesis | 14/2872 | 34/13036 | 1.87 | 0.009379251 | 0.035952007 | BP |
| GO:1990138 | neuron projection extension | 45/2872 | 147/13036 | 1.39 | 0.009379459 | 0.035952007 | BP |
| GO:0034109 | homotypic cell-cell adhesion | 23/2872 | 65/13036 | 1.61 | 0.009449172 | 0.036119077 | BP |
| GO:0042116 | macrophage activation | 23/2872 | 65/13036 | 1.61 | 0.009449172 | 0.036119077 | BP |
| GO:0046634 | regulation of alpha-beta T cell activation | 23/2872 | 65/13036 | 1.61 | 0.009449172 | 0.036119077 | BP |
| GO:0000187 | activation of MAPK activity | 40/2872 | 128/13036 | 1.42 | 0.009557427 | 0.036499235 | BP |
| GO:0045669 | positive regulation of osteoblast differentiation | 19/2872 | 51/13036 | 1.69 | 0.009598733 | 0.03662326 | BP |
| GO:0032652 | regulation of interleukin-1 production | 21/2872 | 58/13036 | 1.64 | 0.009621727 | 0.036677247 | BP |
| GO:1902106 | negative regulation of leukocyte differentiation | 26/2872 | 76/13036 | 1.55 | 0.00983826 | 0.037433842 | BP |
| GO:2000117 | negative regulation of cysteine-type endopeptidase activity | 26/2872 | 76/13036 | 1.55 | 0.00983826 | 0.037433842 | BP |
| GO:0051341 | regulation of oxidoreductase activity | 29/2872 | 87/13036 | 1.51 | 0.009900397 | 0.03763574 | BP |
| GO:0048511 | rhythmic process | 69/2872 | 242/13036 | 1.29 | 0.010156079 | 0.038375932 | BP |
| GO:0010976 | positive regulation of neuron projection development | 71/2872 | 250/13036 | 1.29 | 0.010159237 | 0.038375932 | BP |
| GO:1903844 | regulation of cellular response to transforming growth factor beta stimulus | 33/2872 | 102/13036 | 1.47 | 0.010166256 | 0.038375932 | BP |
| GO:0019362 | pyridine nucleotide metabolic process | 13/2872 | 31/13036 | 1.9 | 0.010178388 | 0.038375932 | BP |
| GO:0032731 | positive regulation of interleukin-1 beta production | 13/2872 | 31/13036 | 1.9 | 0.010178388 | 0.038375932 | BP |
| GO:0034405 | response to fluid shear stress | 13/2872 | 31/13036 | 1.9 | 0.010178388 | 0.038375932 | BP |
| GO:0046496 | nicotinamide nucleotide metabolic process | 13/2872 | 31/13036 | 1.9 | 0.010178388 | 0.038375932 | BP |
| GO:0051150 | regulation of smooth muscle cell differentiation | 13/2872 | 31/13036 | 1.9 | 0.010178388 | 0.038375932 | BP |
| GO:0072525 | pyridine-containing compound biosynthetic process | 13/2872 | 31/13036 | 1.9 | 0.010178388 | 0.038375932 | BP |
| GO:0021872 | forebrain generation of neurons | 16/2872 | 41/13036 | 1.77 | 0.010288688 | 0.038594345 | BP |
| GO:0045576 | mast cell activation | 16/2872 | 41/13036 | 1.77 | 0.010288688 | 0.038594345 | BP |
| GO:0071622 | regulation of granulocyte chemotaxis | 16/2872 | 41/13036 | 1.77 | 0.010288688 | 0.038594345 | BP |
| GO:2000351 | regulation of endothelial cell apoptotic process | 16/2872 | 41/13036 | 1.77 | 0.010288688 | 0.038594345 | BP |
| GO:0006883 | cellular sodium ion homeostasis | 6/2872 | 10/13036 | 2.72 | 0.010441044 | 0.038594345 | BP |
| GO:0009404 | toxin metabolic process | 6/2872 | 10/13036 | 2.72 | 0.010441044 | 0.038594345 | BP |
| GO:0010273 | detoxification of copper ion | 6/2872 | 10/13036 | 2.72 | 0.010441044 | 0.038594345 | BP |
| GO:0015803 | branched-chain amino acid transport | 6/2872 | 10/13036 | 2.72 | 0.010441044 | 0.038594345 | BP |
| GO:0016081 | synaptic vesicle docking | 6/2872 | 10/13036 | 2.72 | 0.010441044 | 0.038594345 | BP |
| GO:0019371 | cyclooxygenase pathway | 6/2872 | 10/13036 | 2.72 | 0.010441044 | 0.038594345 | BP |
| GO:0031650 | regulation of heat generation | 6/2872 | 10/13036 | 2.72 | 0.010441044 | 0.038594345 | BP |
| GO:0033690 | positive regulation of osteoblast proliferation | 6/2872 | 10/13036 | 2.72 | 0.010441044 | 0.038594345 | BP |
| GO:0035810 | positive regulation of urine volume | 6/2872 | 10/13036 | 2.72 | 0.010441044 | 0.038594345 | BP |
| GO:0042749 | regulation of circadian sleep/wake cycle | 6/2872 | 10/13036 | 2.72 | 0.010441044 | 0.038594345 | BP |
| GO:0048245 | eosinophil chemotaxis | 6/2872 | 10/13036 | 2.72 | 0.010441044 | 0.038594345 | BP |
| GO:0051124 | synaptic growth at neuromuscular junction | 6/2872 | 10/13036 | 2.72 | 0.010441044 | 0.038594345 | BP |
| GO:0070234 | positive regulation of T cell apoptotic process | 6/2872 | 10/13036 | 2.72 | 0.010441044 | 0.038594345 | BP |
| GO:0090331 | negative regulation of platelet aggregation | 6/2872 | 10/13036 | 2.72 | 0.010441044 | 0.038594345 | BP |
| GO:1903978 | regulation of microglial cell activation | 6/2872 | 10/13036 | 2.72 | 0.010441044 | 0.038594345 | BP |
| GO:1905288 | vascular associated smooth muscle cell apoptotic process | 6/2872 | 10/13036 | 2.72 | 0.010441044 | 0.038594345 | BP |
| GO:1905459 | regulation of vascular associated smooth muscle cell apoptotic process | 6/2872 | 10/13036 | 2.72 | 0.010441044 | 0.038594345 | BP |
| GO:1990169 | stress response to copper ion | 6/2872 | 10/13036 | 2.72 | 0.010441044 | 0.038594345 | BP |
| GO:0001942 | hair follicle development | 22/2872 | 62/13036 | 1.61 | 0.01060484 | 0.03909527 | BP |
| GO:0032637 | interleukin-8 production | 22/2872 | 62/13036 | 1.61 | 0.01060484 | 0.03909527 | BP |
| GO:0051057 | positive regulation of small GTPase mediated signal transduction | 22/2872 | 62/13036 | 1.61 | 0.01060484 | 0.03909527 | BP |
| GO:0050806 | positive regulation of synaptic transmission | 39/2872 | 125/13036 | 1.42 | 0.010699263 | 0.039329741 | BP |
| GO:0042446 | hormone biosynthetic process | 18/2872 | 48/13036 | 1.7 | 0.010706374 | 0.039329741 | BP |
| GO:0060038 | cardiac muscle cell proliferation | 18/2872 | 48/13036 | 1.7 | 0.010706374 | 0.039329741 | BP |
| GO:0071300 | cellular response to retinoic acid | 18/2872 | 48/13036 | 1.7 | 0.010706374 | 0.039329741 | BP |
| GO:0010595 | positive regulation of endothelial cell migration | 31/2872 | 95/13036 | 1.48 | 0.010938795 | 0.040112537 | BP |
| GO:0030449 | regulation of complement activation | 12/2872 | 28/13036 | 1.95 | 0.010967826 | 0.040112537 | BP |
| GO:0044060 | regulation of endocrine process | 12/2872 | 28/13036 | 1.95 | 0.010967826 | 0.040112537 | BP |
| GO:0090022 | regulation of neutrophil chemotaxis | 12/2872 | 28/13036 | 1.95 | 0.010967826 | 0.040112537 | BP |
| GO:2000403 | positive regulation of lymphocyte migration | 12/2872 | 28/13036 | 1.95 | 0.010967826 | 0.040112537 | BP |
| GO:0014896 | muscle hypertrophy | 25/2872 | 73/13036 | 1.55 | 0.011061014 | 0.040382131 | BP |
| GO:0035023 | regulation of Rho protein signal transduction | 25/2872 | 73/13036 | 1.55 | 0.011061014 | 0.040382131 | BP |
| GO:0009409 | response to cold | 15/2872 | 38/13036 | 1.79 | 0.011366089 | 0.041386619 | BP |
| GO:0010043 | response to zinc ion | 15/2872 | 38/13036 | 1.79 | 0.011366089 | 0.041386619 | BP |
| GO:0030514 | negative regulation of BMP signaling pathway | 15/2872 | 38/13036 | 1.79 | 0.011366089 | 0.041386619 | BP |
| GO:0003333 | amino acid transmembrane transport | 23/2872 | 66/13036 | 1.58 | 0.011545681 | 0.041930116 | BP |
| GO:0018958 | phenol-containing compound metabolic process | 23/2872 | 66/13036 | 1.58 | 0.011545681 | 0.041930116 | BP |
| GO:0120193 | tight junction organization | 23/2872 | 66/13036 | 1.58 | 0.011545681 | 0.041930116 | BP |
| GO:0002695 | negative regulation of leukocyte activation | 37/2872 | 118/13036 | 1.42 | 0.011659924 | 0.042307962 | BP |
| GO:0003416 | endochondral bone growth | 11/2872 | 25/13036 | 2 | 0.011704993 | 0.042360311 | BP |
| GO:0060045 | positive regulation of cardiac muscle cell proliferation | 11/2872 | 25/13036 | 2 | 0.011704993 | 0.042360311 | BP |
| GO:1900745 | positive regulation of p38MAPK cascade | 11/2872 | 25/13036 | 2 | 0.011704993 | 0.042360311 | BP |
| GO:0010660 | regulation of muscle cell apoptotic process | 21/2872 | 59/13036 | 1.62 | 0.011904804 | 0.042941521 | BP |
| GO:2000300 | regulation of synaptic vesicle exocytosis | 21/2872 | 59/13036 | 1.62 | 0.011904804 | 0.042941521 | BP |
| GO:0043551 | regulation of phosphatidylinositol 3-kinase activity | 17/2872 | 45/13036 | 1.71 | 0.01193412 | 0.042941521 | BP |
| GO:0090303 | positive regulation of wound healing | 17/2872 | 45/13036 | 1.71 | 0.01193412 | 0.042941521 | BP |
| GO:0099024 | plasma membrane invagination | 17/2872 | 45/13036 | 1.71 | 0.01193412 | 0.042941521 | BP |
| GO:0051606 | detection of stimulus | 55/2872 | 188/13036 | 1.33 | 0.011952325 | 0.042941521 | BP |
| GO:0002066 | columnar/cuboidal epithelial cell development | 19/2872 | 52/13036 | 1.66 | 0.01206753 | 0.042941521 | BP |
| GO:0015800 | acidic amino acid transport | 19/2872 | 52/13036 | 1.66 | 0.01206753 | 0.042941521 | BP |
| GO:0030104 | water homeostasis | 19/2872 | 52/13036 | 1.66 | 0.01206753 | 0.042941521 | BP |
| GO:0050886 | endocrine process | 19/2872 | 52/13036 | 1.66 | 0.01206753 | 0.042941521 | BP |
| GO:0060760 | positive regulation of response to cytokine stimulus | 19/2872 | 52/13036 | 1.66 | 0.01206753 | 0.042941521 | BP |
| GO:0001502 | cartilage condensation | 7/2872 | 13/13036 | 2.44 | 0.012093379 | 0.042941521 | BP |
| GO:0003184 | pulmonary valve morphogenesis | 7/2872 | 13/13036 | 2.44 | 0.012093379 | 0.042941521 | BP |
| GO:0007350 | blastoderm segmentation | 7/2872 | 13/13036 | 2.44 | 0.012093379 | 0.042941521 | BP |
| GO:0010002 | cardioblast differentiation | 7/2872 | 13/13036 | 2.44 | 0.012093379 | 0.042941521 | BP |
| GO:0010561 | negative regulation of glycoprotein biosynthetic process | 7/2872 | 13/13036 | 2.44 | 0.012093379 | 0.042941521 | BP |
| GO:0022410 | circadian sleep/wake cycle process | 7/2872 | 13/13036 | 2.44 | 0.012093379 | 0.042941521 | BP |
| GO:0030213 | hyaluronan biosynthetic process | 7/2872 | 13/13036 | 2.44 | 0.012093379 | 0.042941521 | BP |
| GO:0030540 | female genitalia development | 7/2872 | 13/13036 | 2.44 | 0.012093379 | 0.042941521 | BP |
| GO:0048532 | anatomical structure arrangement | 7/2872 | 13/13036 | 2.44 | 0.012093379 | 0.042941521 | BP |
| GO:0051580 | regulation of neurotransmitter uptake | 7/2872 | 13/13036 | 2.44 | 0.012093379 | 0.042941521 | BP |
| GO:1900221 | regulation of amyloid-beta clearance | 7/2872 | 13/13036 | 2.44 | 0.012093379 | 0.042941521 | BP |
| GO:0001516 | prostaglandin biosynthetic process | 10/2872 | 22/13036 | 2.06 | 0.012322971 | 0.043275106 | BP |
| GO:0003148 | outflow tract septum morphogenesis | 10/2872 | 22/13036 | 2.06 | 0.012322971 | 0.043275106 | BP |
| GO:0016486 | peptide hormone processing | 10/2872 | 22/13036 | 2.06 | 0.012322971 | 0.043275106 | BP |
| GO:0033081 | regulation of T cell differentiation in thymus | 10/2872 | 22/13036 | 2.06 | 0.012322971 | 0.043275106 | BP |
| GO:0034110 | regulation of homotypic cell-cell adhesion | 10/2872 | 22/13036 | 2.06 | 0.012322971 | 0.043275106 | BP |
| GO:0043368 | positive T cell selection | 10/2872 | 22/13036 | 2.06 | 0.012322971 | 0.043275106 | BP |
| GO:0046457 | prostanoid biosynthetic process | 10/2872 | 22/13036 | 2.06 | 0.012322971 | 0.043275106 | BP |
| GO:0051955 | regulation of amino acid transport | 10/2872 | 22/13036 | 2.06 | 0.012322971 | 0.043275106 | BP |
| GO:0060740 | prostate gland epithelium morphogenesis | 10/2872 | 22/13036 | 2.06 | 0.012322971 | 0.043275106 | BP |
| GO:0086005 | ventricular cardiac muscle cell action potential | 10/2872 | 22/13036 | 2.06 | 0.012322971 | 0.043275106 | BP |
| GO:0090075 | relaxation of muscle | 10/2872 | 22/13036 | 2.06 | 0.012322971 | 0.043275106 | BP |
| GO:0099560 | synaptic membrane adhesion | 10/2872 | 22/13036 | 2.06 | 0.012322971 | 0.043275106 | BP |
| GO:0140448 | signaling receptor ligand precursor processing | 10/2872 | 22/13036 | 2.06 | 0.012322971 | 0.043275106 | BP |
| GO:0003300 | cardiac muscle hypertrophy | 24/2872 | 70/13036 | 1.56 | 0.012441037 | 0.043609784 | BP |
| GO:0006835 | dicarboxylic acid transport | 24/2872 | 70/13036 | 1.56 | 0.012441037 | 0.043609784 | BP |
| GO:0032368 | regulation of lipid transport | 27/2872 | 81/13036 | 1.51 | 0.012520329 | 0.043609784 | BP |
| GO:0003229 | ventricular cardiac muscle tissue development | 14/2872 | 35/13036 | 1.82 | 0.012523424 | 0.043609784 | BP |
| GO:0014911 | positive regulation of smooth muscle cell migration | 14/2872 | 35/13036 | 1.82 | 0.012523424 | 0.043609784 | BP |
| GO:0030857 | negative regulation of epithelial cell differentiation | 14/2872 | 35/13036 | 1.82 | 0.012523424 | 0.043609784 | BP |
| GO:0046688 | response to copper ion | 14/2872 | 35/13036 | 1.82 | 0.012523424 | 0.043609784 | BP |
| GO:0071548 | response to dexamethasone | 14/2872 | 35/13036 | 1.82 | 0.012523424 | 0.043609784 | BP |
| GO:0120178 | steroid hormone biosynthetic process | 14/2872 | 35/13036 | 1.82 | 0.012523424 | 0.043609784 | BP |
| GO:1902622 | regulation of neutrophil migration | 14/2872 | 35/13036 | 1.82 | 0.012523424 | 0.043609784 | BP |
| GO:0050680 | negative regulation of epithelial cell proliferation | 35/2872 | 111/13036 | 1.43 | 0.012691978 | 0.043724056 | BP |
| GO:0002675 | positive regulation of acute inflammatory response | 9/2872 | 19/13036 | 2.15 | 0.012717245 | 0.043724056 | BP |
| GO:0045606 | positive regulation of epidermal cell differentiation | 9/2872 | 19/13036 | 2.15 | 0.012717245 | 0.043724056 | BP |
| GO:0045662 | negative regulation of myoblast differentiation | 9/2872 | 19/13036 | 2.15 | 0.012717245 | 0.043724056 | BP |
| GO:0120255 | olefinic compound biosynthetic process | 9/2872 | 19/13036 | 2.15 | 0.012717245 | 0.043724056 | BP |
| GO:1902932 | positive regulation of alcohol biosynthetic process | 9/2872 | 19/13036 | 2.15 | 0.012717245 | 0.043724056 | BP |
| GO:0002021 | response to dietary excess | 8/2872 | 16/13036 | 2.27 | 0.012724921 | 0.043724056 | BP |
| GO:0003159 | morphogenesis of an endothelium | 8/2872 | 16/13036 | 2.27 | 0.012724921 | 0.043724056 | BP |
| GO:0007271 | synaptic transmission, cholinergic | 8/2872 | 16/13036 | 2.27 | 0.012724921 | 0.043724056 | BP |
| GO:0010766 | negative regulation of sodium ion transport | 8/2872 | 16/13036 | 2.27 | 0.012724921 | 0.043724056 | BP |
| GO:0046885 | regulation of hormone biosynthetic process | 8/2872 | 16/13036 | 2.27 | 0.012724921 | 0.043724056 | BP |
| GO:0060749 | mammary gland alveolus development | 8/2872 | 16/13036 | 2.27 | 0.012724921 | 0.043724056 | BP |
| GO:0061154 | endothelial tube morphogenesis | 8/2872 | 16/13036 | 2.27 | 0.012724921 | 0.043724056 | BP |
| GO:0061377 | mammary gland lobule development | 8/2872 | 16/13036 | 2.27 | 0.012724921 | 0.043724056 | BP |
| GO:0061436 | establishment of skin barrier | 8/2872 | 16/13036 | 2.27 | 0.012724921 | 0.043724056 | BP |
| GO:0086012 | membrane depolarization during cardiac muscle cell action potential | 8/2872 | 16/13036 | 2.27 | 0.012724921 | 0.043724056 | BP |
| GO:0060395 | SMAD protein signal transduction | 22/2872 | 63/13036 | 1.59 | 0.012980606 | 0.044528829 | BP |
| GO:0120192 | tight junction assembly | 22/2872 | 63/13036 | 1.59 | 0.012980606 | 0.044528829 | BP |
| GO:0032874 | positive regulation of stress-activated MAPK cascade | 42/2872 | 138/13036 | 1.38 | 0.013059328 | 0.044761855 | BP |
| GO:1903409 | reactive oxygen species biosynthetic process | 28/2872 | 85/13036 | 1.5 | 0.013190731 | 0.045174914 | BP |
| GO:0010883 | regulation of lipid storage | 16/2872 | 42/13036 | 1.73 | 0.013291423 | 0.045332589 | BP |
| GO:0032371 | regulation of sterol transport | 16/2872 | 42/13036 | 1.73 | 0.013291423 | 0.045332589 | BP |
| GO:0046622 | positive regulation of organ growth | 16/2872 | 42/13036 | 1.73 | 0.013291423 | 0.045332589 | BP |
| GO:0048168 | regulation of neuronal synaptic plasticity | 16/2872 | 42/13036 | 1.73 | 0.013291423 | 0.045332589 | BP |
| GO:0055025 | positive regulation of cardiac muscle tissue development | 16/2872 | 42/13036 | 1.73 | 0.013291423 | 0.045332589 | BP |
| GO:0017015 | regulation of transforming growth factor beta receptor signaling pathway | 32/2872 | 100/13036 | 1.45 | 0.013326423 | 0.045414613 | BP |
| GO:0033627 | cell adhesion mediated by integrin | 20/2872 | 56/13036 | 1.62 | 0.013367487 | 0.045479814 | BP |
| GO:0046579 | positive regulation of Ras protein signal transduction | 20/2872 | 56/13036 | 1.62 | 0.013367487 | 0.045479814 | BP |
| GO:0051402 | neuron apoptotic process | 57/2872 | 197/13036 | 1.31 | 0.013447848 | 0.04571572 | BP |
| GO:0007435 | salivary gland morphogenesis | 13/2872 | 32/13036 | 1.84 | 0.013751211 | 0.046594232 | BP |
| GO:0009948 | anterior/posterior axis specification | 13/2872 | 32/13036 | 1.84 | 0.013751211 | 0.046594232 | BP |
| GO:0055023 | positive regulation of cardiac muscle tissue growth | 13/2872 | 32/13036 | 1.84 | 0.013751211 | 0.046594232 | BP |
| GO:0070741 | response to interleukin-6 | 13/2872 | 32/13036 | 1.84 | 0.013751211 | 0.046594232 | BP |
| GO:0007368 | determination of left/right symmetry | 29/2872 | 89/13036 | 1.48 | 0.013821764 | 0.046795059 | BP |
| GO:0003281 | ventricular septum development | 21/2872 | 60/13036 | 1.59 | 0.014599884 | 0.049348898 | BP |
| GO:0071277 | cellular response to calcium ion | 21/2872 | 60/13036 | 1.59 | 0.014599884 | 0.049348898 | BP |
| GO:0030100 | regulation of endocytosis | 52/2872 | 178/13036 | 1.33 | 0.014614335 | 0.049357516 | BP |
| GO:0055067 | monovalent inorganic cation homeostasis | 35/2872 | 112/13036 | 1.42 | 0.014643754 | 0.049416634 | BP |
| GO:0002460 | adaptive immune response based on somatic recombination of immune receptors built from immunoglobulin superfamily domains | 54/2872 | 186/13036 | 1.32 | 0.014770486 | 0.049777387 | BP |
| GO:0051489 | regulation of filopodium assembly | 15/2872 | 39/13036 | 1.75 | 0.014786663 | 0.049777387 | BP |
| GO:0070228 | regulation of lymphocyte apoptotic process | 15/2872 | 39/13036 | 1.75 | 0.014786663 | 0.049777387 | BP |
| GO:0046620 | regulation of organ growth | 27/2872 | 82/13036 | 1.49 | 0.014841331 | 0.049920899 | BP |
| GO:0031012 | extracellular matrix | 197/3005 | 400/13557 | 2.22 | 1.05E-33 | 5.79E-31 | CC |
| GO:0062023 | collagen-containing extracellular matrix | 155/3005 | 317/13557 | 2.21 | 2.89E-26 | 8.00E-24 | CC |
| GO:0005788 | endoplasmic reticulum lumen | 94/3005 | 241/13557 | 1.76 | 2.02E-09 | 3.73E-07 | CC |
| GO:0043235 | receptor complex | 101/3005 | 268/13557 | 1.7 | 4.50E-09 | 6.22E-07 | CC |
| GO:0009897 | external side of plasma membrane | 80/3005 | 199/13557 | 1.81 | 6.43E-09 | 7.11E-07 | CC |
| GO:0098797 | plasma membrane protein complex | 131/3005 | 382/13557 | 1.55 | 2.48E-08 | 2.28E-06 | CC |
| GO:0097060 | synaptic membrane | 96/3005 | 261/13557 | 1.66 | 4.38E-08 | 3.46E-06 | CC |
| GO:0005604 | basement membrane | 40/3005 | 82/13557 | 2.2 | 9.48E-08 | 6.55E-06 | CC |
| GO:1902495 | transmembrane transporter complex | 72/3005 | 190/13557 | 1.71 | 5.66E-07 | 3.47E-05 | CC |
| GO:0045211 | postsynaptic membrane | 69/3005 | 181/13557 | 1.72 | 7.48E-07 | 4.14E-05 | CC |
| GO:0031225 | anchored component of membrane | 42/3005 | 96/13557 | 1.97 | 1.88E-06 | 9.46E-05 | CC |
| GO:1990351 | transporter complex | 72/3005 | 198/13557 | 1.64 | 3.33E-06 | 0.000153382 | CC |
| GO:0034702 | ion channel complex | 66/3005 | 178/13557 | 1.67 | 3.95E-06 | 0.000167767 | CC |
| GO:0005581 | collagen trimer | 29/3005 | 59/13557 | 2.22 | 4.47E-06 | 0.000176391 | CC |
| GO:0034703 | cation channel complex | 53/3005 | 137/13557 | 1.75 | 8.45E-06 | 0.000311442 | CC |
| GO:0031091 | platelet alpha granule | 32/3005 | 71/13557 | 2.03 | 1.47E-05 | 0.000506059 | CC |
| GO:0098552 | side of membrane | 114/3005 | 362/13557 | 1.42 | 2.01E-05 | 0.000654499 | CC |
| GO:0048786 | presynaptic active zone | 29/3005 | 64/13557 | 2.04 | 3.22E-05 | 0.000988868 | CC |
| GO:0099699 | integral component of synaptic membrane | 35/3005 | 83/13557 | 1.9 | 3.45E-05 | 0.001004225 | CC |
| GO:0045177 | apical part of cell | 96/3005 | 299/13557 | 1.45 | 3.87E-05 | 0.001070228 | CC |
| GO:0032432 | actin filament bundle | 30/3005 | 69/13557 | 1.96 | 6.20E-05 | 0.00163114 | CC |
| GO:0016324 | apical plasma membrane | 81/3005 | 247/13557 | 1.48 | 6.79E-05 | 0.00164549 | CC |
| GO:0099240 | intrinsic component of synaptic membrane | 37/3005 | 92/13557 | 1.81 | 7.14E-05 | 0.00164549 | CC |
| GO:0031093 | platelet alpha granule lumen | 24/3005 | 51/13557 | 2.12 | 7.22E-05 | 0.00164549 | CC |
| GO:0098858 | actin-based cell projection | 63/3005 | 182/13557 | 1.56 | 7.44E-05 | 0.00164549 | CC |
| GO:0045121 | membrane raft | 85/3005 | 263/13557 | 1.46 | 8.12E-05 | 0.001725 | CC |
| GO:0005911 | cell-cell junction | 117/3005 | 385/13557 | 1.37 | 8.84E-05 | 0.001799366 | CC |
| GO:0098857 | membrane microdomain | 85/3005 | 264/13557 | 1.45 | 9.41E-05 | 0.001799366 | CC |
| GO:0072562 | blood microparticle | 28/3005 | 64/13557 | 1.97 | 9.44E-05 | 0.001799366 | CC |
| GO:0098636 | protein complex involved in cell adhesion | 15/3005 | 27/13557 | 2.51 | 0.0001632 | 0.002966188 | CC |
| GO:0098802 | plasma membrane signaling receptor complex | 46/3005 | 126/13557 | 1.65 | 0.000166389 | 0.002966188 | CC |
| GO:0099055 | integral component of postsynaptic membrane | 27/3005 | 63/13557 | 1.93 | 0.000190541 | 0.003290588 | CC |
| GO:0005796 | Golgi lumen | 26/3005 | 60/13557 | 1.95 | 0.000200671 | 0.003360518 | CC |
| GO:0008305 | integrin complex | 14/3005 | 25/13557 | 2.53 | 0.000241559 | 0.003926263 | CC |
| GO:0098589 | membrane region | 85/3005 | 272/13557 | 1.41 | 0.000288608 | 0.004556974 | CC |
| GO:0034705 | potassium channel complex | 24/3005 | 55/13557 | 1.97 | 0.000307112 | 0.004714441 | CC |
| GO:0098936 | intrinsic component of postsynaptic membrane | 28/3005 | 68/13557 | 1.86 | 0.000328764 | 0.004910419 | CC |
| GO:0098644 | complex of collagen trimers | 11/3005 | 18/13557 | 2.76 | 0.00041205 | 0.005992423 | CC |
| GO:0016323 | basolateral plasma membrane | 61/3005 | 185/13557 | 1.49 | 0.000432516 | 0.006128763 | CC |
| GO:0030175 | filopodium | 36/3005 | 96/13557 | 1.69 | 0.000454658 | 0.006281461 | CC |
| GO:0005614 | interstitial matrix | 8/3005 | 11/13557 | 3.28 | 0.00049603 | 0.006685904 | CC |
| GO:0042629 | mast cell granule | 10/3005 | 16/13557 | 2.82 | 0.000594405 | 0.007821116 | CC |
| GO:0044853 | plasma membrane raft | 33/3005 | 87/13557 | 1.71 | 0.000609236 | 0.007829835 | CC |
| GO:0044297 | cell body | 129/3005 | 453/13557 | 1.28 | 0.000817386 | 0.01026621 | CC |
| GO:0008076 | voltage-gated potassium channel complex | 22/3005 | 52/13557 | 1.91 | 0.000900612 | 0.011060151 | CC |
| GO:0009925 | basal plasma membrane | 20/3005 | 46/13557 | 1.96 | 0.001009323 | 0.01186774 | CC |
| GO:0098948 | intrinsic component of postsynaptic specialization membrane | 20/3005 | 46/13557 | 1.96 | 0.001009323 | 0.01186774 | CC |
| GO:0098978 | glutamatergic synapse | 87/3005 | 290/13557 | 1.35 | 0.00104326 | 0.011925227 | CC |
| GO:0099060 | integral component of postsynaptic specialization membrane | 19/3005 | 43/13557 | 1.99 | 0.00105737 | 0.011925227 | CC |
| GO:0042383 | sarcolemma | 38/3005 | 107/13557 | 1.6 | 0.001081691 | 0.011955528 | CC |
| GO:0098793 | presynapse | 112/3005 | 390/13557 | 1.3 | 0.001270938 | 0.013546163 | CC |
| GO:0043025 | neuronal cell body | 113/3005 | 394/13557 | 1.29 | 0.001274629 | 0.013546163 | CC |
| GO:0099634 | postsynaptic specialization membrane | 30/3005 | 80/13557 | 1.69 | 0.00130864 | 0.013645204 | CC |
| GO:0005901 | caveola | 27/3005 | 70/13557 | 1.74 | 0.00137758 | 0.014098042 | CC |
| GO:0031674 | I band | 39/3005 | 112/13557 | 1.57 | 0.001433069 | 0.01439926 | CC |
| GO:0042734 | presynaptic membrane | 36/3005 | 102/13557 | 1.59 | 0.001636031 | 0.015995066 | CC |
| GO:0001527 | microfibril | 7/3005 | 10/13557 | 3.16 | 0.001649777 | 0.015995066 | CC |
| GO:0031092 | platelet alpha granule membrane | 9/3005 | 15/13557 | 2.71 | 0.001707427 | 0.016268584 | CC |
| GO:0045178 | basal part of cell | 24/3005 | 61/13557 | 1.78 | 0.00181544 | 0.017004564 | CC |
| GO:0042641 | actomyosin | 27/3005 | 72/13557 | 1.69 | 0.002231001 | 0.02054869 | CC |
| GO:0014704 | intercalated disc | 17/3005 | 39/13557 | 1.97 | 0.002289421 | 0.020603763 | CC |
| GO:0001725 | stress fiber | 24/3005 | 62/13557 | 1.75 | 0.002348829 | 0.020603763 | CC |
| GO:0097517 | contractile actin filament bundle | 24/3005 | 62/13557 | 1.75 | 0.002348829 | 0.020603763 | CC |
| GO:0030018 | Z disc | 36/3005 | 104/13557 | 1.56 | 0.002402283 | 0.0207434 | CC |
| GO:0015629 | actin cytoskeleton | 117/3005 | 419/13557 | 1.26 | 0.00290075 | 0.024662249 | CC |
| GO:0005902 | microvillus | 26/3005 | 70/13557 | 1.68 | 0.003110689 | 0.026046435 | CC |
| GO:0044291 | cell-cell contact zone | 23/3005 | 60/13557 | 1.73 | 0.003319259 | 0.027378016 | CC |
| GO:0005583 | fibrillar collagen trimer | 7/3005 | 11/13557 | 2.87 | 0.003668833 | 0.029384246 | CC |
| GO:0098643 | banded collagen fibril | 7/3005 | 11/13557 | 2.87 | 0.003668833 | 0.029384246 | CC |
| GO:0031528 | microvillus membrane | 11/3005 | 22/13557 | 2.26 | 0.003755324 | 0.029647291 | CC |
| GO:0046658 | anchored component of plasma membrane | 16/3005 | 39/13557 | 1.85 | 0.006272965 | 0.048825892 | CC |
| GO:0048018 | receptor ligand activity | 122/2925 | 247/13293 | 2.24 | 1.12E-21 | 9.17E-19 | MF |
| GO:0030546 | signaling receptor activator activity | 122/2925 | 252/13293 | 2.2 | 9.62E-21 | 3.95E-18 | MF |
| GO:0030545 | receptor regulator activity | 128/2925 | 272/13293 | 2.14 | 2.35E-20 | 6.43E-18 | MF |
| GO:0005125 | cytokine activity | 67/2925 | 121/13293 | 2.52 | 1.14E-15 | 2.30E-13 | MF |
| GO:0004888 | transmembrane signaling receptor activity | 176/2925 | 463/13293 | 1.73 | 1.40E-15 | 2.30E-13 | MF |
| GO:0005201 | extracellular matrix structural constituent | 69/2925 | 130/13293 | 2.41 | 7.70E-15 | 1.05E-12 | MF |
| GO:0005539 | glycosaminoglycan binding | 79/2925 | 159/13293 | 2.26 | 1.10E-14 | 1.30E-12 | MF |
| GO:0008201 | heparin binding | 59/2925 | 116/13293 | 2.31 | 7.31E-12 | 7.50E-10 | MF |
| GO:0008083 | growth factor activity | 53/2925 | 104/13293 | 2.32 | 7.69E-11 | 7.02E-09 | MF |
| GO:0005509 | calcium ion binding | 166/2925 | 483/13293 | 1.56 | 1.48E-10 | 1.21E-08 | MF |
| GO:0004930 | G protein-coupled receptor activity | 79/2925 | 202/13293 | 1.78 | 2.39E-08 | 1.78E-06 | MF |
| GO:0015267 | channel activity | 98/2925 | 269/13293 | 1.66 | 3.72E-08 | 2.35E-06 | MF |
| GO:0022803 | passive transmembrane transporter activity | 98/2925 | 269/13293 | 1.66 | 3.72E-08 | 2.35E-06 | MF |
| GO:0005178 | integrin binding | 52/2925 | 119/13293 | 1.99 | 9.56E-08 | 5.61E-06 | MF |
| GO:0005216 | ion channel activity | 91/2925 | 251/13293 | 1.65 | 1.47E-07 | 7.62E-06 | MF |
| GO:0022836 | gated channel activity | 70/2925 | 179/13293 | 1.78 | 1.48E-07 | 7.62E-06 | MF |
| GO:0030414 | peptidase inhibitor activity | 47/2925 | 105/13293 | 2.03 | 1.62E-07 | 7.85E-06 | MF |
| GO:0005261 | cation channel activity | 73/2925 | 190/13293 | 1.75 | 1.86E-07 | 8.47E-06 | MF |
| GO:0046873 | metal ion transmembrane transporter activity | 92/2925 | 259/13293 | 1.61 | 3.65E-07 | 1.58E-05 | MF |
| GO:0061135 | endopeptidase regulator activity | 48/2925 | 111/13293 | 1.97 | 4.31E-07 | 1.77E-05 | MF |
| GO:1901681 | sulfur compound binding | 73/2925 | 194/13293 | 1.71 | 4.81E-07 | 1.88E-05 | MF |
| GO:0004866 | endopeptidase inhibitor activity | 44/2925 | 102/13293 | 1.96 | 1.40E-06 | 5.21E-05 | MF |
| GO:0005126 | cytokine receptor binding | 63/2925 | 165/13293 | 1.74 | 1.61E-06 | 5.75E-05 | MF |
| GO:0061134 | peptidase regulator activity | 56/2925 | 143/13293 | 1.78 | 2.40E-06 | 8.23E-05 | MF |
| GO:0001228 | DNA-binding transcription activator activity, RNA polymerase II-specific | 119/2925 | 370/13293 | 1.46 | 3.01E-06 | 9.88E-05 | MF |
| GO:0001216 | DNA-binding transcription activator activity | 119/2925 | 373/13293 | 1.45 | 4.66E-06 | 0.000147182 | MF |
| GO:0015276 | ligand-gated ion channel activity | 31/2925 | 66/13293 | 2.13 | 5.98E-06 | 0.000175478 | MF |
| GO:0022834 | ligand-gated channel activity | 31/2925 | 66/13293 | 2.13 | 5.98E-06 | 0.000175478 | MF |
| GO:0004222 | metalloendopeptidase activity | 35/2925 | 80/13293 | 1.99 | 1.12E-05 | 0.000316353 | MF |
| GO:0003779 | actin binding | 113/2925 | 357/13293 | 1.44 | 1.20E-05 | 0.000328283 | MF |
| GO:0099094 | ligand-gated cation channel activity | 27/2925 | 56/13293 | 2.19 | 1.29E-05 | 0.000342395 | MF |
| GO:0005244 | voltage-gated ion channel activity | 44/2925 | 110/13293 | 1.82 | 1.50E-05 | 0.000372093 | MF |
| GO:0022832 | voltage-gated channel activity | 44/2925 | 110/13293 | 1.82 | 1.50E-05 | 0.000372093 | MF |
| GO:0030020 | extracellular matrix structural constituent conferring tensile strength | 19/2925 | 34/13293 | 2.54 | 1.76E-05 | 0.000424716 | MF |
| GO:0008009 | chemokine activity | 15/2925 | 24/13293 | 2.84 | 2.22E-05 | 0.00052146 | MF |
| GO:0042379 | chemokine receptor binding | 18/2925 | 32/13293 | 2.56 | 2.60E-05 | 0.000592456 | MF |
| GO:0022890 | inorganic cation transmembrane transporter activity | 114/2925 | 371/13293 | 1.4 | 4.69E-05 | 0.001040078 | MF |
| GO:0005516 | calmodulin binding | 54/2925 | 150/13293 | 1.64 | 5.93E-05 | 0.001274778 | MF |
| GO:0005267 | potassium channel activity | 27/2925 | 60/13293 | 2.05 | 6.06E-05 | 0.001274778 | MF |
| GO:0004867 | serine-type endopeptidase inhibitor activity | 24/2925 | 51/13293 | 2.14 | 6.37E-05 | 0.001307554 | MF |
| GO:0022843 | voltage-gated cation channel activity | 32/2925 | 76/13293 | 1.91 | 6.57E-05 | 0.001315939 | MF |
| GO:0050840 | extracellular matrix binding | 24/2925 | 52/13293 | 2.1 | 9.38E-05 | 0.001833667 | MF |
| GO:0016709 | oxidoreductase activity, acting on paired donors, with incorporation or reduction of molecular oxygen, NAD(P)H as one donor, and incorporation of one atom of oxygen | 16/2925 | 29/13293 | 2.51 | 0.00010047 | 0.001889311 | MF |
| GO:0070851 | growth factor receptor binding | 40/2925 | 104/13293 | 1.75 | 0.000101248 | 0.001889311 | MF |
| GO:0008324 | cation transmembrane transporter activity | 121/2925 | 407/13293 | 1.35 | 0.000135553 | 0.002426451 | MF |
| GO:0008528 | G protein-coupled peptide receptor activity | 24/2925 | 53/13293 | 2.06 | 0.000135943 | 0.002426451 | MF |
| GO:0019955 | cytokine binding | 38/2925 | 99/13293 | 1.74 | 0.000156862 | 0.002696875 | MF |
| GO:0005518 | collagen binding | 28/2925 | 66/13293 | 1.93 | 0.000157663 | 0.002696875 | MF |
| GO:0001664 | G protein-coupled receptor binding | 62/2925 | 184/13293 | 1.53 | 0.000161668 | 0.002708938 | MF |
| GO:0140375 | immune receptor activity | 30/2925 | 73/13293 | 1.87 | 0.000185601 | 0.003008506 | MF |
| GO:0016638 | oxidoreductase activity, acting on the CH-NH2 group of donors | 11/2925 | 17/13293 | 2.94 | 0.000186875 | 0.003008506 | MF |
| GO:0001653 | peptide receptor activity | 24/2925 | 54/13293 | 2.02 | 0.000194085 | 0.003064496 | MF |
| GO:0008237 | metallopeptidase activity | 50/2925 | 142/13293 | 1.6 | 0.000204783 | 0.003172406 | MF |
| GO:0004620 | phospholipase activity | 30/2925 | 75/13293 | 1.82 | 0.000326241 | 0.004960392 | MF |
| GO:0004896 | cytokine receptor activity | 26/2925 | 62/13293 | 1.91 | 0.000333799 | 0.004983028 | MF |
| GO:0015079 | potassium ion transmembrane transporter activity | 32/2925 | 82/13293 | 1.77 | 0.000355642 | 0.005133246 | MF |
| GO:0016641 | oxidoreductase activity, acting on the CH-NH2 group of donors, oxygen as acceptor | 9/2925 | 13/13293 | 3.15 | 0.000356366 | 0.005133246 | MF |
| GO:0001540 | amyloid-beta binding | 24/2925 | 56/13293 | 1.95 | 0.000379311 | 0.005369563 | MF |
| GO:0045125 | bioactive lipid receptor activity | 8/2925 | 11/13293 | 3.31 | 0.000470235 | 0.00654386 | MF |
| GO:0004629 | phospholipase C activity | 12/2925 | 21/13293 | 2.6 | 0.000492662 | 0.006741688 | MF |
| GO:0004497 | monooxygenase activity | 24/2925 | 57/13293 | 1.91 | 0.000519955 | 0.006998531 | MF |
| GO:0042056 | chemoattractant activity | 12/2925 | 22/13293 | 2.48 | 0.000867663 | 0.011490273 | MF |
| GO:0005249 | voltage-gated potassium channel activity | 20/2925 | 46/13293 | 1.98 | 0.000915315 | 0.011928916 | MF |
| GO:0017046 | peptide hormone binding | 13/2925 | 25/13293 | 2.36 | 0.000961843 | 0.012339438 | MF |
| GO:0042277 | peptide binding | 65/2925 | 207/13293 | 1.43 | 0.001007833 | 0.012468443 | MF |
| GO:0005230 | extracellular ligand-gated ion channel activity | 14/2925 | 28/13293 | 2.27 | 0.001019451 | 0.012468443 | MF |
| GO:0005044 | scavenger receptor activity | 17/2925 | 37/13293 | 2.09 | 0.001032643 | 0.012468443 | MF |
| GO:0030594 | neurotransmitter receptor activity | 17/2925 | 37/13293 | 2.09 | 0.001032643 | 0.012468443 | MF |
| GO:0003953 | NAD+ nucleosidase activity | 8/2925 | 12/13293 | 3.03 | 0.001137917 | 0.012976243 | MF |
| GO:0031005 | filamin binding | 8/2925 | 12/13293 | 3.03 | 0.001137917 | 0.012976243 | MF |
| GO:0050135 | NAD(P)+ nucleosidase activity | 8/2925 | 12/13293 | 3.03 | 0.001137917 | 0.012976243 | MF |
| GO:0061809 | NAD+ nucleotidase, cyclic ADP-ribose generating | 8/2925 | 12/13293 | 3.03 | 0.001137917 | 0.012976243 | MF |
| GO:0008194 | UDP-glycosyltransferase activity | 36/2925 | 101/13293 | 1.62 | 0.001166234 | 0.013055641 | MF |
| GO:0015085 | calcium ion transmembrane transporter activity | 32/2925 | 87/13293 | 1.67 | 0.001176682 | 0.013055641 | MF |
| GO:0043177 | organic acid binding | 45/2925 | 134/13293 | 1.53 | 0.001298961 | 0.014108917 | MF |
| GO:0004435 | phosphatidylinositol phospholipase C activity | 11/2925 | 20/13293 | 2.5 | 0.001305979 | 0.014108917 | MF |
| GO:0042805 | actinin binding | 13/2925 | 26/13293 | 2.27 | 0.001538955 | 0.016225934 | MF |
| GO:0008376 | acetylgalactosaminyltransferase activity | 16/2925 | 35/13293 | 2.08 | 0.001541464 | 0.016225934 | MF |
| GO:0004714 | transmembrane receptor protein tyrosine kinase activity | 21/2925 | 51/13293 | 1.87 | 0.00160193 | 0.016375193 | MF |
| GO:0042562 | hormone binding | 21/2925 | 51/13293 | 1.87 | 0.00160193 | 0.016375193 | MF |
| GO:0030021 | extracellular matrix structural constituent conferring compression resistance | 9/2925 | 15/13293 | 2.73 | 0.001615476 | 0.016375193 | MF |
| GO:0019956 | chemokine binding | 10/2925 | 18/13293 | 2.52 | 0.001969128 | 0.019716561 | MF |
| GO:0051015 | actin filament binding | 55/2925 | 174/13293 | 1.44 | 0.0020025 | 0.01980913 | MF |
| GO:0031406 | carboxylic acid binding | 44/2925 | 133/13293 | 1.5 | 0.002039257 | 0.01993259 | MF |
| GO:0004857 | enzyme inhibitor activity | 79/2925 | 267/13293 | 1.34 | 0.002120026 | 0.020301481 | MF |
| GO:0022824 | transmitter-gated ion channel activity | 11/2925 | 21/13293 | 2.38 | 0.002200628 | 0.020301481 | MF |
| GO:0022835 | transmitter-gated channel activity | 11/2925 | 21/13293 | 2.38 | 0.002200628 | 0.020301481 | MF |
| GO:0071813 | lipoprotein particle binding | 11/2925 | 21/13293 | 2.38 | 0.002200628 | 0.020301481 | MF |
| GO:0071814 | protein-lipid complex binding | 11/2925 | 21/13293 | 2.38 | 0.002200628 | 0.020301481 | MF |
| GO:0017080 | sodium channel regulator activity | 12/2925 | 24/13293 | 2.27 | 0.002328717 | 0.021244435 | MF |
| GO:0043394 | proteoglycan binding | 13/2925 | 27/13293 | 2.19 | 0.002375487 | 0.021315226 | MF |
| GO:0005231 | excitatory extracellular ligand-gated ion channel activity | 8/2925 | 13/13293 | 2.8 | 0.002388398 | 0.021315226 | MF |
| GO:0016247 | channel regulator activity | 33/2925 | 94/13293 | 1.6 | 0.00243449 | 0.021492949 | MF |
| GO:0015081 | sodium ion transmembrane transporter activity | 29/2925 | 80/13293 | 1.65 | 0.002521134 | 0.022021106 | MF |
| GO:0016701 | oxidoreductase activity, acting on single donors with incorporation of molecular oxygen | 9/2925 | 16/13293 | 2.56 | 0.002974634 | 0.025178673 | MF |
| GO:0016702 | oxidoreductase activity, acting on single donors with incorporation of molecular oxygen, incorporation of two atoms of oxygen | 9/2925 | 16/13293 | 2.56 | 0.002974634 | 0.025178673 | MF |
| GO:0016722 | oxidoreductase activity, oxidizing metal ions | 9/2925 | 16/13293 | 2.56 | 0.002974634 | 0.025178673 | MF |
| GO:0015077 | monovalent inorganic cation transmembrane transporter activity | 70/2925 | 235/13293 | 1.35 | 0.003056302 | 0.025605967 | MF |
| GO:0072341 | modified amino acid binding | 25/2925 | 67/13293 | 1.7 | 0.003108469 | 0.025775926 | MF |
| GO:0005262 | calcium channel activity | 27/2925 | 74/13293 | 1.66 | 0.003139376 | 0.025775926 | MF |
| GO:0001968 | fibronectin binding | 12/2925 | 25/13293 | 2.18 | 0.003590446 | 0.029187577 | MF |
| GO:0038024 | cargo receptor activity | 21/2925 | 54/13293 | 1.77 | 0.003669275 | 0.029535956 | MF |
| GO:0001786 | phosphatidylserine binding | 17/2925 | 41/13293 | 1.88 | 0.003981623 | 0.031739048 | MF |
| GO:0030246 | carbohydrate binding | 51/2925 | 164/13293 | 1.41 | 0.004129661 | 0.032602587 | MF |
| GO:0030169 | low-density lipoprotein particle binding | 8/2925 | 14/13293 | 2.6 | 0.004502651 | 0.03487654 | MF |
| GO:0048020 | CCR chemokine receptor binding | 8/2925 | 14/13293 | 2.6 | 0.004502651 | 0.03487654 | MF |
| GO:0016298 | lipase activity | 32/2925 | 94/13293 | 1.55 | 0.004815304 | 0.036949699 | MF |
| GO:0045499 | chemorepellent activity | 11/2925 | 23/13293 | 2.17 | 0.00544077 | 0.041362577 | MF |

| Table S6. Baseline characteristics of the patients for sreum VEGFA analysis | | | | |
| --- | --- | --- | --- | --- |
| Characteristic | Total (n=44) | non-OPLL (n=14) | OPLL (n=30) | p Value |
| Age-yr | 53.93±9.83 | 55.21±11.41 | 53.33±9.16 | 0.56 |
| Female sex-no.(%) | 19(43.18) | 6(42.86) | 13(43.33) | 0.98 |
| Body-mass index | 23.89±2.21 | 23.64±1.12 | 24.00±2.59 | 0.62 |
| Duration of symptoms-mo | 34.05±59.44 | 37.91±94.30 | 32.26±35.28 | 0.77 |
| premenopausal-no.(%) | 7(15.91) | 3(21.43) | 4(13.3) | 0.66 |
| Smoker-no.(%) | 8(18.18) | 1(7.14) | 7(23.33) | 0.40 |
| Alcoholism-no.(%) | 4(9.09) | 1(7.14) | 3(10.00) | 1.00 |
| Hypertension-no.(%) | 15(34.09) | 4(28.57) | 11(36.67) | 0.74 |
| Diabetes-no.(%) | 10(22.72) | 2(14.29) | 8(26.67) | 0.46 |
| NDI score | 0.255±0.01 | 0.218±0.06 | 0.273±0.11 | 0.01 |
| JOA score | 12.36±3.16 | 13.79±1.89 | 11.70±3.43 | 0.03 |

Table S7. Patient and specimen Information for cell culture and staining

| Characteristic | Total (n=18) | non-OPLL (n=8) | OPLL (n=10) | p Value |
| --- | --- | --- | --- | --- |
| Age-yr | 52.22±11.70 | 47.50±15.85 | 56.00±5.25 | 0.18 |
| Female sex-no.(%) | 8(44.44) | 3(37.50) | 5(50.00) | 0.96 |
| Body-mass index | 23.42±1.25 | 23.41±1.32 | 23.42±1.27 | 1.00 |
| Duration of symptoms-mo | 19.92±20.50 | 16.70±21.69 | 22.50±20.28 | 0.95 |
| premenopausal-no.(%) | 7(38.89) | 2(25.00) | 5(50.00) | 0.55 |
| Smoker-no.(%) | 5(27.78) | 2(25.00) | 3(30.00) | 1.00 |
| Alcoholism-no.(%) | 2(11.11) | 0(0.00) | 2(20.00) | 0.48 |
| Hypertension-no.(%) | 6(33.33) | 2(25.00) | 4(40.00) | 0.87 |
| Diabetes-no.(%) | 3(16.67) | 1(12.50) | 2(20.00) | 1.00 |
| NDI score | 0.29±0.12 | 0.23±0.12 | 0.34±0.11 | 0.039 |
| JOA score | 12.72±2.99 | 13.88±2.10 | 11.80±3.36 | 0.15 |

Table S8. Human siRNA sequences

| Name | Sequences |
| --- | --- |
| si-NC-1 | 5ʹ- UUCUCCGAACGUGUCACGUtt-3ʹ |
| si-NC-2 | 5ʹ-ACGUGACACGUUCGGAGAAtt-3ʹ |
| si-LOXL2-1 | 5ʹ-GGGCAGAAGAGGAAGCACAtt-3ʹ |
| si-LOXL2-2 | 5ʹ-UGUGCUUCCUCUUCUGCCCtt-3ʹ |

Table S9. Human real-time PCR primer sequences

| Name | Forward primer | Reverse primer |
| --- | --- | --- |
| GAPDH | 5′-CTGGGCTACACTGAGCACC-3′ | 5′-AAGTGGTCGTTGAGGGCAATG-3′ |
| HIF1A | 5′-CACCACAGGACAGTACAGGAT-3′ | 5′-CGTGCTGAATAATACCACTCACA-3′ |
| LOXL2 | 5′-CTCCTCCTACGGCAAGGGA-3′ | 5′-ATGTCCTCCACCTGGATATTCA -3′ |
| VEGFA | 5′-AGGGCAGAATCATCACGAAGT-3′ | 5′-AGGGTCTCGATTGGATGGCA-3′ |
| VEGFB | 5′-GAGATGTCCCTGGAAGAACACA-3′ | 5′-GAGTGGGATGGGTGATGTCAG-3′ |
| VEGFC | 5′-GAGGAGCAGTTACGGTCTGTG-3′ | 5′-TCCTTTCCTTAGCTGACACTTGT-3′ |
| PDGFB | 5′-CTCGATCCGCTCCTTTGATGA-3′ | 5′-CGTTGGTGCGGTCTATGAG-3′ |
| CD31 | 5′-AACAGTGTTGACATGAAGAGCC-3′ | 5′-TGTAAAACAGCACGTCATCCTT-3′ |
| EMCN | 5′-AGCAACCAGCCGGTCTTATTC-3′ | 5′-AGCACATTCGGTACAAACCCA-3′ |
| SP7 | 5′-GAGGCAACTGGCTAGGTGG-3′ | 5′-CTGGATTAAGGGGAGCAAAGTC-3′ |
| RUNX2 | 5′-TCAACGATCTGAGATTTGTGGG-3′ | 5′-GGGGAGGATTTGTGAAGACGG-3′ |
| COL1A1 | 5′-GTGCGATGACGTGATCTGTGA-3′ | 5′-CGGTGGTTTCTTGGTCGGT-3′ |
| BGLAP | 5′-GGCGCTACCTGTATCAATGG-3′ | 5′-GTGGTCAGCCAACTCGTCA-3′ |

Table S10. Mouse Enpp1 KO and WT primer sequences

| Name | Forward primer | Reverse primer |
| --- | --- | --- |
| Enpp1 KO | 5′-TCCTCCTTTGTCTTCTGACCCTC-3′ | 5′-GATTAGGTAACACAAAAGCCCACC-3′ |
| Enpp1 WT | 5′-GCTGTCTGACGTTTATCACCATCTCC-3′ | 5′-TTGGCGCAGCTTGGTTTCAAC-3′ |
